# Individual and neighborhood socioeconomic inequality and the risk of dementia: A 14‐year follow‐up study

**DOI:** 10.1002/alz.71060

**Published:** 2026-01-04

**Authors:** Xingqi Cao, Shengyi Li, Xueqing Jia, Jingyun Zhang, Guanqun Chao, Weili Liu, Liying Chen, Zuyun Liu

**Affiliations:** ^1^ Department of General Practice, Sir Run Run Shaw Hospital Zhejiang University School of Medicine Hangzhou Zhejiang China; ^2^ Second Affiliated Hospital, Department of Big Data in Health Science School of Public Health, Zhejiang Key Laboratory of Intelligent Preventive Medicine Zhejiang University School of Medicine Hangzhou Zhejiang China; ^3^ Department of Biostatistics Johns Hopkins Bloomberg School of Public Health Baltimore Maryland USA

**Keywords:** cognitive function, dementia, individual socioeconomic status, neighborhood socioeconomic status, white matter hyperintensity

## Abstract

**INTRODUCTION:**

Socioeconomic inequality is a non‐negligible risk factor for dementia. However, complex associations of individual socioeconomic status (ISES) and neighborhood socioeconomic status (NSES) with dementia risk have not been determined.

**METHODS:**

In 327,641 adults aged 40–69 years from the UK Biobank, we estimated the separate, interactive, and joint associations of ISES and NSES with the risk of dementia and explored the role of inflammatory markers and metabolites.

**RESULTS:**

Low ISES and low NSES were associated with higher risks of all‐cause dementia and its subtypes. A stronger association between ISES and dementia was observed in those with low NSES. The subpopulation with disadvantages in both ISES and NSES showed the highest risk of dementia. Inflammatory markers (e.g., lymphocyte percentage) and metabolites (e.g., valine) mediated the associations of socioeconomic status (SES) profiles with all‐cause dementia.

**DISCUSSION:**

These findings underscore the importance of eliminating socioeconomic inequality at both individual and neighborhood levels for preventing dementia.

**Highlights:**

- Disadvantages in both individual socioeconomic status (ISES) and neighborhood socioeconomic status (NSES) are associated with higher risks of dementia.
- There are significant interactions between ISES and NSES on dementia risk.
- The low SES subpopulation with disadvantages in both ISES and NSES has the highest risk of dementia.
- Inflammatory markers and metabolites partially mediate the associations of socioeconomic status (SES) profiles with all‐cause dementia.
- Narrowing socioeconomic inequality at both the individual and neighborhood levels may help prevent dementia.

## 1 BACKGROUND

Dementia is characterized by deterioration in cognitive function (e.g., thinking, reasoning, and remembering)^1^ and has become one of the major causes of dependence and death.^2^ Due to the substantial economic burden for not only the individual with dementia but also their caregivers, families, and society,^3^, ^4^ dementia has been raised as a public health priority. The absence of curative treatment for dementia has prompted researchers to concentrate on identifying its modifiable risk factors to strengthen its early prevention.^5^, ^6^

Educational attainment, a commonly used surrogate to assess individual socioeconomic status (ISES), appears to be protective against dementia risk.^7^ In the latest Lancet Commission on dementia, less education was reported to account for about 5% higher risk of dementia.^8^ Besides, other ISES indicators, such as income level and occupational status, were also found to be associated with dementia.^9^ However, these single indicators cannot reflect overall ISES. Previous studies usually used the additive ISES score to assess the association of overall ISES with adverse health outcomes,^10^, ^11^ ignoring the heterogeneity in these single ISES indicators. Recently, an overall ISES metric constructed through latent class analysis (LCA) has been favored for its outperformance in predicting outcomes compared to the additive ISES score.^12^, ^13^, ^14^ Neighborhood SES (NSES), a proxy that measures multiple aspects, such as access to healthcare resources and critical infrastructure, housing density, and environmental pollutants, also leads to health disparities, but its association with dementia risk has not reached a consensus.^15^, ^16^, ^17^, ^18^ Among studies that have assessed ISES and NSES simultaneously,^15^, ^16^, ^17^ the majority only evaluated their separate associations with dementia.^15^, ^16^ However, in reality, individuals are exposed to complex socioeconomic environments rather than a single exposure. It is imperative to estimate whether ISES was associated with dementia risk, either independently, interactively, or jointly with NSES. Additionally, given that some individuals may have similar socioeconomic status (SES) circumstances (i.e., can be defined as one subpopulation), identifying different subpopulations, that is, distinct SES profiles, is crucial for understanding the extent and how complex socioeconomic inequality contributes to incident dementia.

The mechanisms underlying the associations between socioeconomic inequality and dementia are not fully understood yet. Lower SES is positively associated with inflammation,^19^ which is thought to drive dementia development.^20^ Although the mediating role of inflammation (e.g., C‐reactive protein) on SES‐dementia associations has been revealed, these studies only focus on ISES indicators.^21^, ^22^ Most recently, plasma metabolomics has emerged as a promising tool to help understand the pathophysiology of dementia.^23^, ^24^ SES is linked with metabolism,^25^ but whether socioeconomic inequality‐related metabolic change contributed to SES‐dementia associations remains unclear.

Importantly, as a developed country, the UK has serious and rising socioeconomic inequality,^26^ and such explorations in the UK are warranted. In this study, we leveraged data of middle‐aged and older adults from the UK Biobank (UKB), a prospective cohort study of the UK population. Our study primarily aimed to estimate the complex associations of ISES and NSES with the incidence risk of all‐cause dementia and its subtypes, as well as their associations with cognitive function and brain structure. Second, we explored whether inflammatory markers and metabolites mediated the associations.

## 2 MATERIALS AND METHODS

### 2.1 Study participants

UKB is an ongoing cohort study of more than 500,000 adults recruited from 22 assessment centers across England, Scotland, and Wales between 2006 and 2010 (http://www.ukbiobank.ac.uk/).^27^ UKB was approved by the North West Multi‐Center Research Ethics Committee.^28^ Written informed consent from all participants was obtained.^29^ As shown in Figure S1, there were 499,036 adults aged 40–69 years at baseline. We excluded those with diagnosed cancer, cardiovascular disease, diabetes mellitus, and dementia at baseline (*N* = 94,771) and with missing data on ISES items (i.e., educational attainment, occupational status, and income level; *N* = 60,725), Townsend Deprivation Index (TDI; *N* = 446), and covariates (.e.g., ethnicity and alcohol consumption; *N* = 15,453), leaving an analytic sample of 327,641 adults for primary analyses of dementia incidence. Additionally, due to different numbers of missingness on cognitive test and brain structure assessment, we assembled various analytic samples for analyses of cognitive function and brain structure, respectively, to maximize statistical power.

**RESEARCH IN CONTEXT**

1. **Systematic review**: We systematically searched the literature using databases such as PubMed. Although individual and neighborhood socioeconomic status have been separately reported to be associated with dementia, whether they may independently, interactively, or jointly influence dementia risk remains unclear. Furthermore, the mechanisms underlying the associations are not fully understood.
2. **Interpretation**: In this prospective cohort study of 327,641 UK population, individual and neighborhood socioeconomic inequality were separately, interactively, or jointly associated with the risk of dementia. We identified four subpopulations with distinct socioeconomic status (SES) profiles, and the low SES subpopulation with disadvantages in both ISES and NSES showed the highest risk of dementia. Moreover, inflammation and metabolites partially mediated the associations.
3. **Future directions**: The findings highlight the significance of eliminating socioeconomic inequality at both the individual and neighborhood levels for preventing dementia. Inflammatory and metabolic interventions targeting those vulnerable populations may help promote brain health.

### 2.2 SES

ISES was assessed using educational attainment, occupational status, and income level. Educational attainment was obtained through questionnaires, and participants reported their education qualifications as college or university degree, A levels/Advanced Subsidiary (AS) levels or equivalent, O levels/General Certificate of Secondary Education (GCSEs) or equivalent, Certificate of Secondary Education (CSEs) or equivalent, National Vocational Qualification (NVQ) or Higher National Diploma (HND) or Higher National Certificate (HNC) or equivalent, other professional qualifications, and none of the above (equivalent to less than high school diploma) (Field ID: 6138). The occupational status was classified as employed (i.e., paid employed or self‐employed, retired, doing unpaid or voluntary work, or being a full‐ or part‐time student) and unemployed (Field ID: 6142). The average total household income before tax was classified as greater than £100,000, £52,000–£100,000, £31,000–£51,999, £18,000–£30,999, and less than £18,000 (Field ID: 738). Based on the three ISES items, LCA was used to create an overall ISES variable. According to the Akaike Information Criterion, entropy value, and item‐response probabilities, three latent classes were identified (Table S1), representing high, moderate, and low ISES, respectively (see details in Supporting Information).

NSES was assessed using TDI (Field ID: 22189), which was derived from national census data and calculated based on the information of unemployment, overcrowding, home ownership, and car ownership.^30^ In UKB, each participant was assigned a TDI score corresponding to their postcode of residence before they joined UKB. A lower score of TDI indicates a higher NSES. NSES was classified as high, moderate, and low, corresponding to the lowest, middle, and highest tertile of TDI, respectively.

**FIGURE 1:**
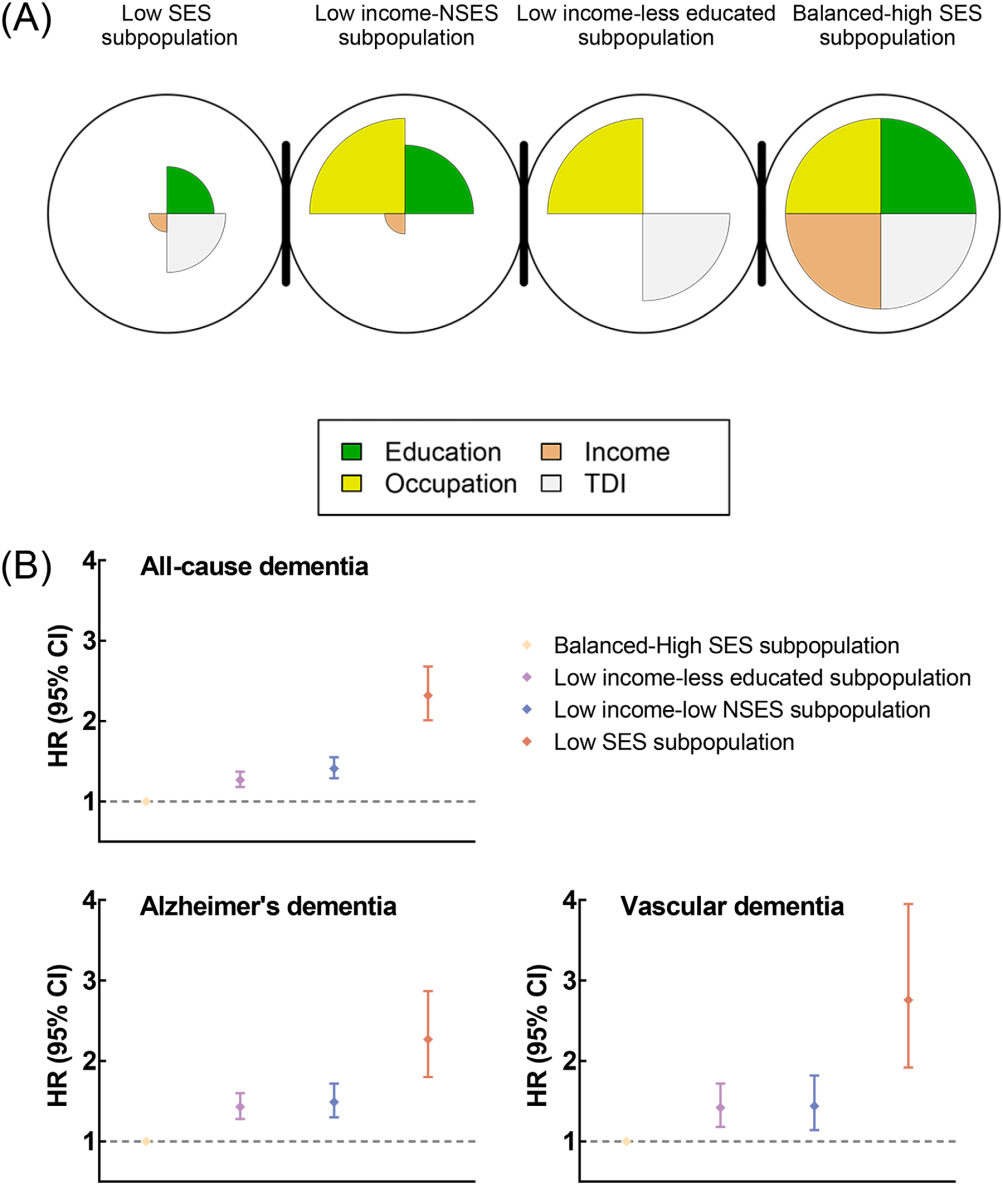
The characteristics of identified SES profiles and the associations of SES profiles with incident dementia. (A) The characteristics of identified SES profiles. (B) Associations of SES profiles with the risks of all‐cause dementia, Alzheimer's dementia, and vascular dementia. The models were adjusted for age, gender, ethnicity, family history of Alzheimer's dementia, apolipoprotein E genotype, smoking status, alcohol consumption, healthy diet, regular exercise, sleep duration, body mass index, waist circumference, systolic blood pressure, and diastolic blood pressure. CI, confidence interval; HR, hazard ratio; NSES, neighborhood socioeconomic status; SES, socioeconomic status; TDI, Townsend deprivation index.

In addition, we performed self‐organizing maps (SOM) on ISES and NSES variables to identify subpopulations with similar SES profiles. SOM, an unsupervised method, helps to identify subpopulations with heterogeneous patterns according to comparable exposure profiles.^31^ The best number of subpopulations was ascertained using the within‐subpopulation sum of squares and between‐subpopulation sum of squares statistics.^31^ In this study, we identified four subpopulations that share similar SES profiles (Figure 1A). We named these subpopulations according to their major SES profiles. Most participants lived in the area of high NSES, had high educational attainment, and were employed with high income (the balanced‐high SES subpopulation; *N* = 155,592, 47.5%). The low income‐less educated subpopulation (*N* = 91,030, 27.8%) had a low income and educational attainment, although they lived in the affluent region and were employed. The lowincome‐ NSES subpopulation (*N* = 61,317, 18.7%) was highly educated and employed but earned less and lived in the area of low NSES. The low SES subpopulation (*N* = 19,702, 6.0%) was employed with low educational attainment and income and lived in the area of moderate NSES.

### 2.3 Primary outcomes

Primary outcomes were incident dementia, cognitive function, and brain structure. All‐cause dementia and its subtypes (including Alzheimer's dementia and vascular dementia) in UKB were ascertained using self‐reported medical history (for the ascertainment of prevalent cases only) and linked hospital admission records using the International Classification of Diseases 9th edition (ICD‐9) (all‐cause dementia: 290.2, 290.3, 290.4, 291.2, 294.1, 331.0, 331.1, 331.2, 331.5; Alzheimer's dementia: 331.0; vascular dementia: 290.4) and 10th edition (ICD‐10) (all‐cause dementia: A81.0, F00, F01, F02, F03, F05.1, F10.6, G30, G31.0, G31.1, G31.8, I67.3; Alzheimer's dementia: F00, G30; vascular dementia: F01, I67.3) codes until October 31, 2022. The time‐to‐event for each adult was defined as the period from the date of recruitment to the date of the first occurrence of interest event (i.e., dementia), death, loss to follow‐up, or end of follow‐up, whichever came first.

At baseline, cognitive functions, including visual memory, processing speed, verbal/numeric reasoning, and prospective memory, were assessed through a touchscreen interface.^32^ Visual memory was assessed by a pair matching test, which was scored as the number of errors when recalling the positions of pairs of matching cards. Processing speed was assessed by reaction time test, which was scored as mean duration (in milliseconds) to correctly match cards with matching symbols. Verbal/numeric reasoning was assessed by a fluid intelligence test, which was scored as the number of correct answers to timed reasoning and logic questions. Prospective memory, which measured individual memory for the future task, was assessed with an on‐screen introduction to be recalled later and was defined as a binary variable (right or wrong). For the test of visual memory and processing speed, higher raw scores indicate worse performance, while for verbal/numeric reasoning, higher raw scores indicate better performance. Visual memory scores were ln(*x* + 1)‐transformed due to skewed distribution and zero inflation, and processing speed scores were log‐transformed due to skewed distribution. Then, visual memory, processing speed, and verbal/numeric reasoning scores were *z*‐standardized to facilitate comparison.

The volume of white matter hyperintensity (WMH) was assessed by magnetic resonance imaging (MRI) with a 3 Tesla Siemens Skyra scanner since 2014. The volume (in mm^3^) of WMH was derived through T2‐weighted imaging and normalized for head size. Because of a skewed distribution, the volume of WMH was naturally logarithmically transformed and then *z*‐standardized.

### 2.4 Covariates

Several covariates were considered in our study, including age, gender, ethnicity, family histories of disease, apolipoprotein E (APOE) genotypes, smoking status, alcohol consumption, healthy diet, regular exercise, sleep duration, body mass index (BMI), waist circumference (WC), systolic blood pressure (SBP), and diastolic blood pressure (DBP). Ethnicity included White, Mixed, Asian, Black, Chinese, and other backgrounds. Family history of Alzheimer's disease was classified as yes and no. APOE genotype was defined based on two single nucleotide polymorphisms (i.e., *rs7412* and *rs429358*) and classified as ε4‐carrier and non ε4‐carrier according to a previous study.^33^ Smoking status was classified as never, previous, and current smoker. Alcohol consumption was classified as never or special occasions only, one to three times per month, one to four times per week, and daily or almost daily. A healthy diet was classified as yes (≥5 portions of a variety of fruit and vegetables per day) and no.^34^ Regular physical activity was classified as yes (150 min of moderate activity, 75 min of vigorous activity, or an equivalent combination) and no.^35^ Average sleep duration (hours) per day was self‐reported. Weight (kg), height (m), WC (cm), SBP (mmHg), and DBP (mmHg) were measured by trained staff. BMI (kg/m^2^) was calculated as the weight (kg) divided by the square of the height (m). Average values of two measures of SBP and DBP were calculated.

### 2.5 Plasma inflammatory markers and metabolites

Data of plasma inflammatory markers and metabolites were obtained from baseline blood tests (https://biobank.ndph.ox.ac.uk/showcase/showcase/docs/haematology.pdf and https://biobank.ndph.ox.ac.uk/showcase/ukb/docs/nmrm_companion_doc.pdf). Inflammatory markers were measured using the Beckman Coulter LH750 Hematology Analyzer. We included C‐reactive protein and the count or percentage of leukocytes, neutrophils, monocytes, lymphocytes, and platelets. To reflect systematic inflammatory status, specific ratios, including Systemic Immune‐Inflammation Index (SII, neutrophil count*platelet/lymphocyte count), neutrophil‐to‐lymphocyte ratio (NLR, neutrophil count/lymphocyte count), platelet‐to‐lymphocyte ratio (PLR, platelet/lymphocyte count), and lymphocyte‐to‐monocyte ratio (LMR, lymphocyte count/monocyte count), were constructed. Metabolites were measured with a high‐throughput nuclear magnetic resonance (NMR) metabolomics platform. We included 168 metabolites available in absolute levels, which comprised lipoprotein lipids, fatty acids, and various low‐molecular‐weight metabolites. All the inflammatory markers and metabolites were transformed using natural logarithmic transformation (ln[*x*] for inflammatory markers and ln[*x* + 1] for metabolites) and then *z*‐standardized for following statistical analyses.

### 2.6 Statistical analysis

Basic characteristics of the analytic sample in total and by SES subgroups were described by median (interquartile ranges [IQRs]) and number (percentage), and differences were compared using the Kruskal–Wallis test and chi‐squared test for continuous and categorical variables, respectively.

First, Cox proportional hazard regression models were conducted to estimate the separate associations of ISES and NSES with the risk of dementia. We used the Schoenfeld residuals test to verify the proportional hazard assumption and found no significant violation. Hazard ratios (HRs) and 95% confidence intervals (CIs) were documented. Three models were conducted: model 1 was adjusted for age, gender, ethnicity, family history of Alzheimer's dementia, and APOE genotypes; model 2 was further adjusted for smoking status, alcohol consumption, healthy diet, regular exercise, sleep duration, BMI, WC, SBP, and DBP based on model 1. We tested the correlation between ISES and NSES (Spearman *r* = 0.169, *p* < 0.001) and examined the multicollinearity issue (variance inflation factor < 2). Thus, to test whether ISES and NSES were independently associated with the risk of dementia, we added ISES and NSES into the model simultaneously based on model 2. In addition, to quantify the separate contributions of ISES and NSES to incident dementia, we calculated partial population‐attributable risk (PAR) percentages of incident dementia for low ISES and NSES using the SAS macro of PAR%, which was a previously well‐established method of multivariable‐adjusted PAR.^36^ Considering that low ISES and low NSES subgroups showed higher incidence risks of dementia, as well as the calculation requirement of PAR, we combined moderate and high subgroups before calculation. Subsequently, we conducted a stratified analysis by NSES to estimate the associations of ISES with the risk of dementia in different NSES subgroups. We documented the estimates from model 2. To quantify the multiplicative and additive interactions, we additionally included a product term between ISES (low, moderate, and high) and NSES (low, moderate, and high) in the model. HR with its 95% CI of the product term was calculated to measure interaction on a multiplicative scale. Relative excess risk due to interaction (RERI) and corresponding 95% CI was calculated to measure interaction on an additive scale.^37^ Third, to estimate the joint associations, we classified participants into nine groups according to ISES (low, moderate, and high) and NSES (low, moderate, and high) and estimated HRs of incident dementia in different groups compared with those with high ISES and high NSES. In addition, we estimated the associations of SES profiles (i.e., four subpopulations) with the risk of dementia using Cox proportional hazards regression models, adjusting for all covariates.

Furthermore, we conducted additional association analyses with dementia as the outcome by (1) further adjusting for the genetic risk of Alzheimer's dementia (Field ID: 26206), given that the incidence risk of dementia was partly determined by the genetic susceptibility, and the genetic risk of dementia differed by SES; (2) setting the end of follow‐up at the occurrence of coronavirus disease 2019 (COVID‐19) (November 30, 2019), to minimize the impact of COVID‐19; (3) excluding those who followed up for less than 2 years, to reduce selection bias; and (4) stratifying by age groups (<60 years and ≥60 years), gender (female and male) and APOE genotypes (non e4‐carrier, and e4‐carrier), to test potential variations of joint associations in different subgroups. To boost explaining the influence of ISES and NSES on brain health, cognitive function, and brain structure were thus explored following the above analysis procedures. General linear regression models and logistic regression models (only for the analysis of prospective memory) were conducted to estimate their cross‐sectional associations. There were three follow‐up assessments for cognitive function and one follow‐up assessment for brain structure in UKB, while only about 20% of the population attended these follow‐up assessments. We further analyzed longitudinal associations of ISES and NSES with changes in cognitive function and brain structure (see details in Supporting Information).

Lastly, we performed exploratory analyses for the mediating role of inflammatory markers and metabolites in the association between SES profiles and incident all‐cause dementia. All models were adjusted for all covariates. False discovery rate (FDR) adjusted *p* < 0.05 indicated statistical significance. First, the associations of SES profiles with inflammatory markers and metabolites were estimated using the general linear regression model. Then, the associations of inflammatory markers and metabolites with the risk of all‐cause dementia were estimated using the Cox proportional hazard regression model. Finally, we performed mediation analyses for those variables significantly and consistently associated with SES profiles and the incidence risk of all‐cause dementia. The R package “mediation” with 1000 simulations was used, and mediation proportions and corresponding 95% CIs were documented.

All statistical analyses were performed using SAS version 9.4 (SAS Institute, Cary, NC, USA) and R version 4.3.1 (2023‐06‐16). Statistical significance was defined as a two‐tailed *p* < 0.05 unless noted otherwise.

## 3 RESULTS

### 3.1 Basic characteristics

The median age of the 327,641 recruited participants was 56.4 (IQR: 49.1, 62.4) years. The majority were female (53.7%) and White (95.6%). Participants with low SES were more likely to be female and non‐White, compared to the balanced‐high SES subpopulation. The detailed baseline characteristics of the total participants and by SES profile subgroups are shown in Table S2. Similar basic characteristics by ISES and NSES subgroups were observed (Tables S3 and S4).

### 3.2 Separate associations of ISES and NSES with the incidence risk of dementia

During a median follow‐up of 14 years, 1.2% (4028/327,641), 0.6% (1845/327,641), and 0.2% (672/327,641) of the participants developed all‐cause dementia, Alzheimer's dementia, and vascular dementia, respectively.

**FIGURE 2:**
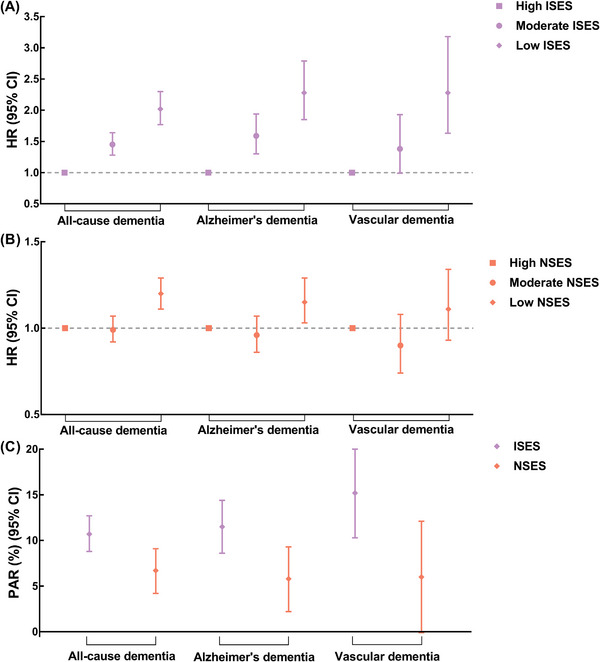
Separate associations of ISES and NSES with incident dementia, and partial PAR percentages for incident dementia associated with lower ISES and NSES. (A) The associations of ISES with the risks of all‐cause dementia, Alzheimer's dementia, and vascular dementia. (B) The associations of NSES with the risks of all‐cause dementia, Alzheimer's dementia, and vascular dementia. (C) Partial PAR percentages for incident all‐cause dementia, Alzheimer's dementia, and vascular dementia associated with low ISES and NSES. The models were adjusted for ISES, NSES, age, gender, ethnicity, family history of Alzheimer's dementia, apolipoprotein E genotypes, smoking status, alcohol consumption, healthy diet, regular exercise, sleep duration, body mass index, waist circumference, systolic blood pressure, and diastolic blood pressure. CI, confidence interval; HR, hazard ratio; ISES, individual socioeconomic status; NSES, neighborhood socioeconomic status; PAR, population‐attributable risk.

After adjusting for all covariates, low ISES and NSES were positively associated with the incidence risks of all‐cause dementia, Alzheimer's dementia, and vascular dementia (Table S5). For participants with low ISES, the multivariable‐adjusted HRs were 2.07 (95% CI 1.82, 2.36) for all‐cause dementia, 2.31 (95% CI 1.89, 2.84) for Alzheimer's dementia, and 2.30 (95% CI 1.65, 3.22) for vascular dementia, compared with their counterparts with high ISES. For participants with low NSES, the multivariable‐adjusted HRs were 1.29 (95% CI 1.20, 1.40) for all‐cause dementia, 1.25 (95% CI 1.12, 1.40) for Alzheimer's dementia, and 1.25 (95% CI 1.04, 1.50) for vascular dementia, compared with their counterparts with high NSES. Both ISES and NSES were independently associated with the incidence risk of dementia. When ISES and NSES were added into the models simultaneously, the results remained almost unchanged (Figure 2A,B and Table S5). When we further adjusted for the genetic risk of Alzheimer's dementia (Table S6), setting the end of follow‐up at the occurrence of COVID‐19 (Table S7), or excluding those who were followed up for less than 2 years (Table S8), the significant results remained.

We found that ISES contributed almost twice as much to the risk of dementia as NSES (Figure 2C and Table S9). Low ISES accounted for a PAR of 10.7% (95% CI 8.8%, 12.7%), 11.5% (95% CI 8.6%, 14.4%), and 15.2% (95% CI 10.3%, 20.0%) for all‐cause dementia, Alzheimer's dementia, and vascular dementia cases, respectively. Nevertheless, low NSES accounted for a PAR of 6.7% (95% CI 4.2%, 9.1%), 5.8% (95% CI 2.2%, 9.3%), and 6.0% (95% CI −0.1%, 12.1%) for all‐cause dementia, Alzheimer's dementia, and vascular dementia cases, respectively.

### 3.3 Interactive associations of ISES and NSES with the incidence risk of dementia

**FIGURE 3:**
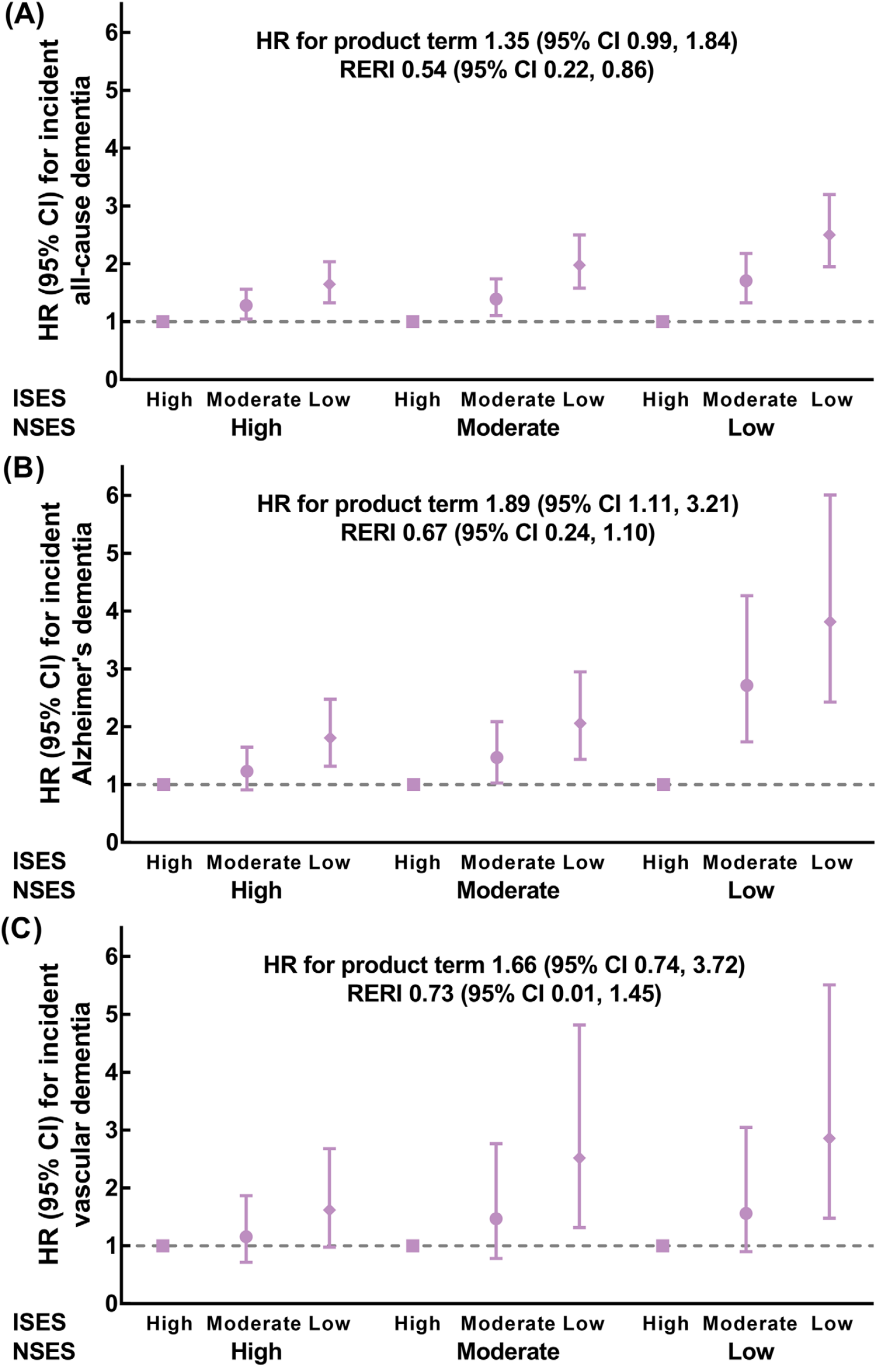
Associations of ISES with the risks of dementia stratified by NSES (*N* = 327,641). (A) Associations of ISES with the risk of all‐cause dementia stratified by NSES. (B) Associations of ISES with the risk of Alzheimer's dementia stratified by NSES. (C) Associations of ISES with the risk of vascular dementia stratified by NSES. The models were adjusted for age, gender, ethnicity, family history of Alzheimer's dementia, apolipoprotein E genotype, smoking status, alcohol consumption, healthy diet, regular exercise, sleep duration, body mass index, waist circumference, systolic blood pressure, and diastolic blood pressure. Multiplicative interaction was evaluated using hazard ratios for the product term between ISES (low vs. high) and NSES (low vs. high), and the multiplicative interaction was statistically significant when its confidence interval did not include 1. Additive interaction was evaluated using RERI between ISES (low vs. high) and NSES (low vs. high), and the additive interaction was statistically significant when its confidence interval did not include 0. CI, confidence interval; HR, hazard ratio; ISES, individual socioeconomic status; NSES, neighborhood socioeconomic status; RERI, relative excess risks due to interaction.

Low ISES was associated with a higher risk of incident dementia among participants of various NSES subgroups (Figure 3A–C and Table S10). HRs for those with low ISES compared with high ISES for all‐cause dementia were 1.65 (95% CI 1.33, 2.04) among participants with high NSES and 2.50 (95% CI 1.95, 3.20) among those with low NSES, with significant additive interaction (RERI 0.54; 95% CI 0.22, 0.86). Similar patterns were observed for Alzheimer's dementia (RERI 0.67; 95% CI 0.24, 1.10) and vascular dementia (RERI 0.73; 95% CI 0.01, 1.45). The results did not change substantially in all additional analyses (Tables S11–S13).

### 3.4 Joint associations of ISES and NSES with the incidence risk of dementia

Figure S2A–C shows the joint associations of ISES and NSES with the risk of dementia (all *p* for trend <0.001). HRs for participants of low ISES and low NSES subgroup compared with those with high ISES and high NSES were 2.27 (95% CI 1.87, 2.76) for all‐cause dementia, 2.19 (95% CI 1.63, 2.93) for Alzheimer's dementia, and 2.26 (95% CI 1.41, 3.64) for vascular dementia (Table S14). Results were not materially changed in all additional analyses, including stratification analyses by age, gender, and APOE genotypes (Tables S15–S20).

Based on ISES and NSES variables, we used SOM to identify four subpopulations that shared similar SES profiles (Figure 1A). Compared to the balanced‐high SES subpopulation, the other three subpopulations all exhibited significantly higher incidence risk of all‐cause dementia, Alzheimer's dementia, and vascular dementia (Figure 1B and Table S21). For instance, HRs for incidence risks of all‐cause dementia were 1.27 (95% CI 1.18, 1.37), 1.41 (95% CI 1.29, 1.55), and 2.32 (95% CI 2.01, 2.68) for the low income‐less educated subpopulation, low income‐NSES subpopulation, and low SES subpopulation, respectively. The results remained similar in all additional analyses (Tables S22–S27).

### 3.5 Associations of ISES and NSES with cognitive function and brain structure

**FIGURE 4:**
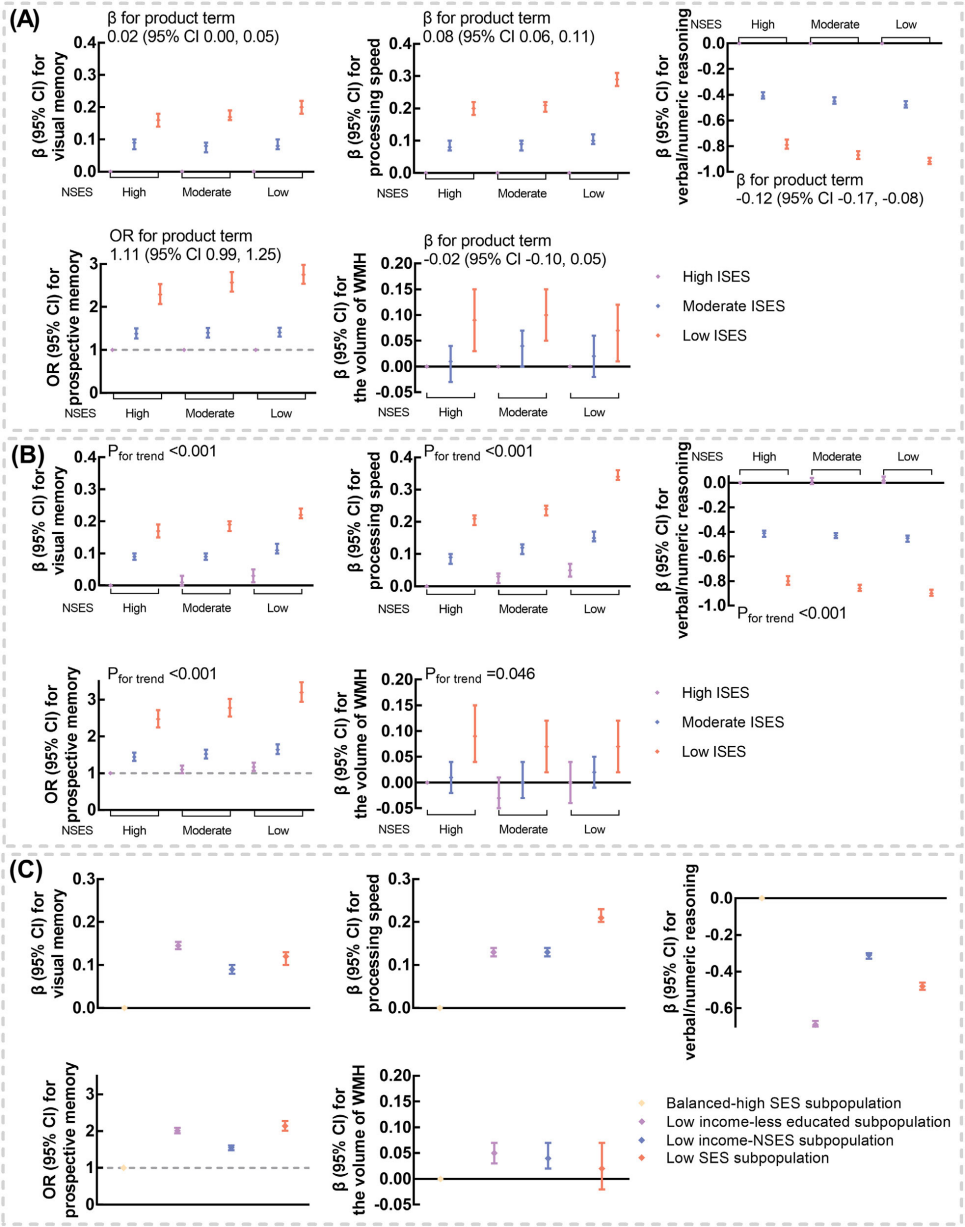
Associations of ISES, NSES, and SES profiles with cognitive function and the volume of WMH. (A) Associations of ISES with cognitive function and the volume of WMH stratified by NSES. The models were adjusted for age, gender, ethnicity, family history of Alzheimer's dementia, apolipoprotein E genotypes, smoking status, alcohol consumption, healthy diet, regular exercise, sleep duration, body mass index, waist circumference, systolic blood pressure, and diastolic blood pressure. Participants with high ISES were set as the reference group. Multiplicative interaction was evaluated using β/OR for the product term between ISES (low vs. high) and NSES (low vs. high), and the multiplicative interaction was statistically significant when its confidence interval did not include 0 for β and 1 for OR. (B) Joint associations of ISES and NSES with cognitive function and the volume of WMH. The models were adjusted for age, gender, ethnicity, family history of Alzheimer's dementia, apolipoprotein E genotypes, smoking status, alcohol consumption, healthy diet, regular exercise, sleep duration, body mass index, waist circumference, systolic blood pressure, and diastolic blood pressure. Participants with high ISES and high NSES were set as the reference group. (C) Associations of SES profiles with cognitive function and the volume of WMH. The models were adjusted for age, gender, ethnicity, family history of Alzheimer's dementia, apolipoprotein E genotypes, smoking status, alcohol consumption, healthy diet, regular exercise, sleep duration, body mass index, waist circumference, systolic blood pressure, and diastolic blood pressure. The balanced‐high SES subpopulation was set as the reference group. CI, confidence interval; ISES, individual socioeconomic status; NSES, neighborhood socioeconomic status; OR, odds ratio; SES, socioeconomic status; WMH, white matter hyperintensity.

Both low ISES and low NSES were noted to be independently associated with a lower level of cognitive function (Table S28). Significant interactions between ISES and NSES were found for visual memory, processing speed, and verbal/numeric reasoning, with more pronounced associations of low ISES with worse cognitive function among those with low NSES (Figure 4A and Table S29). Compared with participants with high ISES and high NSES, *β* for those with low ISES and low NSES was 0.22 (95% CI 0.21, 0.24) for visual memory, 0.34 (95% CI 0.33, 0.36) for processing speed, and −0.90 (95% CI −0.92, −0.87) for verbal/numeric reasoning, and OR was 3.19 (95% CI 2.94, 3.47) for prospective memory (Figure 4B and Table S30). Additionally, compared with the balanced‐high SES subpopulation, the other three subpopulations all exhibited worse cognitive function (Figure 4C and Table S31). For instance, *β* for processing speed was 0.13 (95% CI 0.12, 0.14), 0.13 (95% CI 0.12, 0.14), and 0.21 (95% CI 0.20, 0.23) for the low income‐less educated subpopulation, low ‐income NSES subpopulation, and low‐SES subpopulation, respectively.

Independent association of ISES with the volume of WMH was observed not only in total participants (Table S32), but also among subgroups by NSES (Figure 4A and Table S33). We did not detect a significant interaction between ISES and NSES for the volume of WMH (*p* = 0.687). In joint association analysis, a larger volume of WMH was observed for those with low ISES regardless of the level of NSES (Figure 4B and Table S34). Additionally, compared with the balanced‐high SES subpopulation, the low income‐less educated subpopulation, and the low income‐ NSES subpopulation showed a larger volume of WMH, with *β* of 0.05 (95% CI 0.03, 0.07) and 0.04 (95% CI 0.02, 0.07), respectively (Figure 4C and Table S35).

In addition, we ran additional analyses regarding longitudinal associations of ISES and NSES with changes in cognitive function and the volume of WMH (Tables S36–S43). We found that low ISES was independently associated with cognitive decline. Compared to the balanced‐high SES subpopulation, low income‐less educated subpopulation consistently showed declines in cognitive function. Also, low NSES was positively associated with a higher risk of worse prospective memory. Nevertheless, no significant associations of ISES and NSES with the volume of WMH change were found.

### 3.6 Exploratory analyses of the mediation effect of inflammatory markers and metabolites on the associations of SES profiles with the incidence risk of all‐cause dementia

**FIGURE 5:**
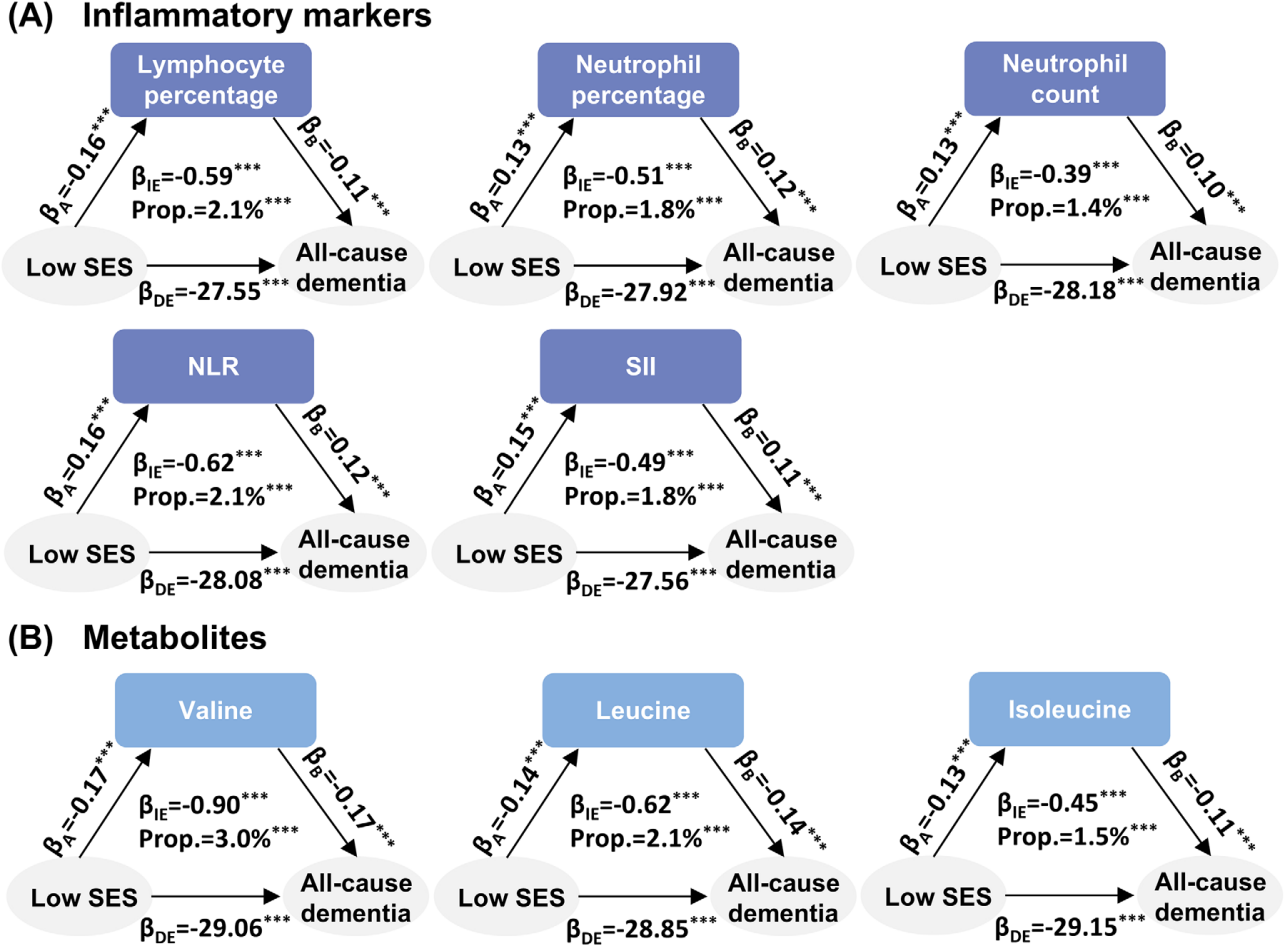
Associations of SES profiles, inflammatory markers, and metabolites, with incident all‐cause dementia. (A) The mediation results between SES profiles, inflammatory markers, and incident all‐cause dementia. (B) The mediation results between SES profiles, metabolites, and incident all‐cause dementia. The models were adjusted for age, gender, ethnicity, family history of Alzheimer's dementia, apolipoprotein E genotypes, smoking status, alcohol consumption, healthy diet, regular exercise, sleep duration, body mass index, waist circumference, systolic blood pressure, and diastolic blood pressure. Only mediators with mediation proportions higher than 1.0% were shown in the plots, and the results for the low SES subpopulation compared to the balanced‐high SES subpopulation were presented. *β*_A_ indicates the effect of the low SES on the mediator. *β*_B_ indicates the effect of the mediator on all‐cause dementia. *β*_IE_ indicates the indirect effect of low SES on all‐cause dementia. *β*_DE_ indicates the direct effect of low SES on all‐cause dementia. Asterisks (^***^) indicate a FDR adjusted *p*‐value < 0.001. DE, direct effect; FDR, false discovery rate; IE, indirect effect; NLR, neutrophil‐to‐lymphocyte ratio; Prop, proportion of mediation; SES, socioeconomic status; SII, systemic immune‐inflammation index.

After correction for multiple testing, SES profiles were significantly associated with all included inflammatory markers and most of the metabolites (Tables S44 and S45). Next, we performed Cox proportional hazard regression models to estimate the associations of inflammatory markers and metabolites with the incidence risk of all‐cause dementia. Ten inflammatory markers and 21 metabolites showed significant associations with both the SES profiles (low SES subpopulation vs. balanced‐high SES subpopulation) and risk of all‐cause dementia with consistent directions (Tables S46 and S47). Finally, we found that the associations of SES profiles (low SES subpopulation vs. balanced‐high SES subpopulation) with the incidence risk of all‐cause dementia were partially mediated by those inflammatory markers and metabolites (Tables S48 and S49), with relatively larger mediation proportions (>1.0%) observed for five inflammatory markers (i.e., lymphocyte percentage, neutrophil percentage, neutrophil count, NLR, and SII) and three metabolites (i.e., valine, leucine, and isoleucine) (Figure 5).

## 4 DISCUSSION

In this large prospective cohort study with up to 14 years of follow‐up, we found that lower ISES and NSES were independently, interactively, and jointly associated with higher risks of dementia. Four distinct subpopulations that shared similar SES profiles were identified, and the subpopulation with the most disadvantaged SES exhibited the highest risk of dementia. Our findings highlight the importance of narrowing socioeconomic inequality at both individual and neighborhood levels in preventing dementia. Moreover, we revealed that several inflammatory markers and metabolites partially mediated the associations of SES profiles with all‐cause dementia, providing clues to plausible biological mechanisms.

Our results that lower ISES was positively associated with the risks of dementia are in line with previous studies,^15^, ^16^, ^38^ supporting the hypothesis that socioeconomic inequality influences health.^39^ Lack of access to healthcare may also increase physiological stress, thus resulting in greater susceptibility to dementia.^40^ By contrast, little attention has been attracted to neighborhood socioeconomic inequality, and the association of NSES with dementia has been inconsistently observed in prior studies,^15^, ^16^, ^17^ with some authors suggesting a null significant association.^15^, ^16^ Another study of 688,507 Chinese adults aged ≥50 years reported that neighborhood socioeconomic advantages increased the risk of dementia by 52%.^17^ The present study extended such exploration to NSES and confirmed the positive associations of lower NSES with the risk of dementia in the UK population. The discrepancy in the study design, sample size, and demographic characteristics may be responsible for the inconclusive findings. Furthermore, we observed that low ISES consistently accounted for almost twice as many cases of dementia as low NSES, suggesting that individual socioeconomic inequality may be a more prominent contributor to dementia. In support of our results, studies from China and the UK found that the association between NSES and mortality was weaker compared with ISES.^12^, ^13^ NSES emphasizes systemic socioeconomic inequality, but it lacks the ability to recognize individual differences and identify social determinants of health disparities.^12^ We caution that the results regarding NSES should be interpreted in context. While validation of our findings is still required, we urge policy‐makers who are developing interventions to tackle brain health disparities to include the elimination of socioeconomic inequality, particularly at the individual level, in those measures.

In our study, we also confirmed that ISES was associated with a higher risk of dementia in the UK population, regardless of NSES. Additive interactions were observed and individuals with low ISES living in disadvantaged areas had a higher risk of dementia than those living in affluent regions. Our results correspond with the “collective resources model,” which highlights the beneficial effect of living in an area with collective material and social resources for individuals with low ISES.^41^ However, a recent study from China has documented a significant interaction between ISES and NSES, showing that individuals with low ISES living in affluent regions had the highest risk of dementia.^17^ The exact reasons for the conflicting findings were unclear but might depend on the definition of ISES and NSES as well as the population characteristics and socio‐cultural background.

Considering that individuals may be exposed to heterogeneous SES profiles, we used SOM to identify four subpopulations across the UK. These subpopulations with distinct SES profiles presented disparities in dementia risk. Compared to the balanced‐high SES subpopulation, the low SES subpopulation, mostly socioeconomically deprived, had the highest risk of all‐cause dementia and its subtypes, indicating the important contribution of socioeconomic inequality at both individual and neighborhood levels to dementia development, which was consistent with the results from the joint association analysis of ISES and NSES. Although the low income‐ NSES subpopulation had higher educational attainment than the low income‐less educated subpopulation, it still exhibited the second‐highest risk of dementia, possibly due to greater neighborhood socioeconomic deprivation. Because the UK was one of the first countries to establish social security, the availability of more community care in affluent regions may help to weaken the negative effect of low educational attainment on dementia. Our findings highlight the need to obtain specific SES profiles to distinguish subpopulations with heterogeneous socioeconomic exposures, helping develop targeted policies to reduce individuals’ socioeconomic inequality and further relieve the dementia burden.

Our study supports previous findings that inflammation partially mediates the association between SES and dementia.^21^, ^22^ Existing evidence has demonstrated that socioeconomic inequality is associated with higher inflammation levels,^19^ which in turn leads to incident dementia.^20^ Moreover, SES was found to be associated with metabolites, including docosahexaenoic acid (DHA) and omega‐3 fatty acids,^25^ and these metabolites contributed to the development of dementia.^42^, ^43^, ^44^ In this study, we further demonstrated that socioeconomic inequality may lead to dementia partially through these metabolites. Intriguingly, relatively larger mediation proportions were found for branched‐chain amino acids (i.e., valine, leucine, and isoleucine), which could be regulated through dietary intervention. Disturbances in branched‐chain amino acids in both the blood and the brain have been previously observed in mouse models of Alzheimer's dementia.^45^ Given the difficulties of addressing socioeconomic inequality, the results suggest that we may mitigate their negative effects on brain health by regulating inflammation and metabolic disturbances. However, considering the low mediation proportion attributable to these inflammation or metabolic changes, the mechanisms underlying SES‐dementia still warrant further research to confirm.

The strengths of this study included the prospective study design, large sample size, and long follow‐up. We simultaneously considered individual and neighborhood socioeconomic inequality in the same population and estimated their separate, interactive, and joint associations with all‐cause dementia and its subtypes, which are scarce in the literature. SOM allows us to identify distinct SES profiles and provides a more comprehensive interpretation of the joint association of ISES and NSES with dementia. In addition, abundant phenotypic data enable us to explore the associations of SES with cognitive function and the volume of WMH, strengthening the evidence that socioeconomic inequality could influence brain health even at the early stage of dementia. Finally, using multiple inflammatory markers and metabolites, we explored the biological mechanisms underlying the associations between SES profiles and dementia and found that inflammation and metabolic disorders play a great role.

Nevertheless, several limitations should also be noted. First, socioeconomic inequality was assessed at a single time point. Thus, we can only speak to the effect of midlife ISES and NSES, rather than that of historical ISES and NSES, on dementia. Future studies should evaluate trajectories of ISES and NSES from childhood to adulthood to better describe the longitudinal associations from a life course perspective. Second, the study participants from UKB had higher levels of SES and tended to be healthier compared to general adults of the same age in the UK.^46^ Due to “healthy volunteer” selection bias, our results may not be generalizable to other populations. Third, future research with repeated assessment of cognitive performance and brain structure could allow us to validate the detrimental effects of socioeconomic inequality on change rates of cognition and brain volume in a prodromal stage. Fourth, despite adjustments for multiple covariates in our study, residual confounding was still inevitable. Finally, the observational study design impeded us to draw the causal inference. Interventional studies are required to validate our findings.

We found that socioeconomic disadvantage, particularly at the individual level, contributed to a higher risk of dementia. Nevertheless, individuals with disadvantaged ISES and NSES had the highest risk of dementia during follow‐up, highlighting the importance of reducing individual socioeconomic inequality, particularly among those living in disadvantaged areas, to relieve the dementia burden. Our study adds to the evidence that systemic inflammation and specific metabolism were plausible pathways linking socioeconomic inequality and dementia.

## CONFLICT OF INTEREST STATEMENT

The authors declare no conflicts of interest. Author disclosures are available in the Supporting Information.

## CONSENT STATEMENT

All participants provided informed consent.

## Supporting information



Supporting Information

Supporting Information
