# Supplementary material for: Individual and neighborhood socioeconomic inequality and the risk of dementia: A 14‐year follow‐up study
Source: Alzheimers Dement. 2026 Jan 4;22(1):e71060. doi: 10.1002/alz.71060 (PMC12765403; doi:10.1002/alz.71060)
Supplement: Supplementary file 1 — Supporting Information [file ALZ-22-e71060-s001.docx]

**Supplementary Materials**

Supplementary Methods

Figure S1. Flow chart of the sample selection.

Figure S2. Joint associations of ISES and NSES with the risk of dementia (N=327,641).

Table S1. Mean posterior probabilities, prevalence of latent classes, and item-related proportion of each latent class.

Table S2. Basic characteristics of the study participants in total and by SES profiles subgroups.

Table S3. Basic characteristics of the study participants in total and by ISES subgroups.

Table S4. Basic characteristics of the study participants in total and by NSES subgroups.

Table S5. Associations of ISES, and NSES with the risk of dementia (N=327,641).

Table S6. Associations of SES with the risk of dementia after further adjusting for genetic risk of Alzheimer’s dementia (N=327,086).

Table S7. Associations of SES with the risk of dementia when setting the end of follow-up at the occurrence of COVID-19 (N=327,641).

Table S8. Associations of SES with the risk of dementia when excluding those who were followed up for less than 2 years.

Table S9. Partial PAR percentages for incident dementia associated with low SES.

Table S10. Associations of ISES with the risk of dementia stratified by NSES (N=327,641).

Table S11. Associations of ISES with the risk of dementia stratified by NSES after further adjusting for genetic risk of Alzheimer’s dementia (N=327,086).

Table S12. Associations of ISES with the risk of dementia stratified by NSES when setting the end of follow-up at the occurrence of COVID-19 (N=327,641).

Table S13. Associations of ISES with the risk of dementia stratified by NSES when excluding those who were followed up for less than 2 years.

Table S14. Joint associations of ISES and NSES with the risk of dementia (N=327,641).

Table S15. Joint associations of ISES and NSES with the risk of dementia after further adjusting for genetic risk of Alzheimer’s dementia (N=327,086).

Table S16. Joint associations of ISES and NSES with the risk of dementia when setting the end of follow-up at the occurrence of COVID-19 (N=327,641).

Table S17. Joint associations of ISES and NSES with the risk of dementia when excluding those who were followed up for less than 2 years.

Table S18. Joint associations of ISES and NSES with the risk of dementia stratified by age (N=327,641).

Table S19. Joint associations of ISES and NSES with the risk of dementia stratified by gender (N=327,641).

Table S20. Joint associations of ISES and NSES with the risk of dementia stratified by APOE genotypes (N=327,641).

Table S21. Associations of SES profiles with the risk of dementia (N=327,641).

Table S22. Associations of SES profiles with the risk of dementia after further adjusting for genetic risk of Alzheimer’s dementia (N=327,086).

Table S23. Associations of SES profiles with the risk of dementia when setting the end of follow-up at the occurrence of COVID-19 (N=327,641).

Table S24. Associations of SES profiles with the risk of dementia when excluding those who were followed up for less than 2 years.

Table S25. Associations of SES profiles with the risk of dementia stratified by age (N=327,641).

Table S26. Associations of SES profiles with the risk of dementia stratified by gender (N=327,641).

Table S27. Associations of SES profiles with the risk of dementia stratified by APOE genotypes (N=327,641).

Table S28. Associations of SES with cognitive function.

Table S29. Associations of ISES with cognitive function stratified by NSES.

Table S30. Joint associations of ISES and NSES with cognitive function.

Table S31. Associations of SES profiles with cognitive function.

Table S32. Associations of SES with the volume of WMH (N=34,591).

Table S33. Associations of ISES with the volume of WMH stratified by NSES (N=34,591).

Table S34. Joint associations of ISES and NSES with the volume of WMH (N=34,591).

Table S35. Associations of SES profiles with the volume of WMH (N=34,591).

Table S36. Associations of SES with cognitive function change.

Table S37. Associations of ISES with cognitive function change stratified by NSES.

Table S38. Joint associations of ISES and NSES with cognitive function change.

Table S39. Associations of SES profiles with cognitive function change.

Table S40. Associations of SES with the volume of WMH change (N=3,709).

Table S41. Associations of ISES with the volume of WMH change stratified by NSES (N=3,709).

Table S42. Joint associations of ISES and NSES with the volume of WMH change (N=3,709).

Table S43. Associations of SES profiles with the volume of WMH change (N=3,709).

Table S44. Associations of SES profiles with blood inflammatory markers (N=303,981).

Table S45. Associations of SES profiles with metabolites (N=174,175).

Table S46. Associations of inflammatory markers with the risk of dementia (N=303,981).

Table S47. Associations of metabolites with the risk of dementia (N=174,175).

Table S48. Mediation proportion of SES profiles in dementia attributed to blood inflammatory markers (N=303,981).

Table S49. Mediation proportion of SES profiles in dementia attributed to metabolites (N=174,175).

Supplementary References

**Supplementary Methods**

**Assessment of ISES**

Individual socioeconomic status (ISES) was assessed using educational attainment, occupational status, and income level. Educational attainment was classified as college or university degree, A levels/AS levels or equivalent, O levels/ General Certificate of Secondary Education (GCSEs) or equivalent, Certificate of Secondary Education (CSEs) or equivalent, National Vocational Qualification (NVQ) or Higher National Diploma (HND) or Higher National Certificate (HNC) or equivalent, other professional qualifications, and none of the above (equivalent to less than high school diploma) (Field ID: 6138). The occupational status was classified as employed (i.e., paid employed or self-employed, retired, doing unpaid or voluntary work, or being a full or part-time student), and unemployed (Field ID: 6142). The average total household income before tax was classified as greater than £100,000, £52,000 to £100,000, £31,000 to £51,999, £18,000 to £30,999, and less than £18,000 (Field ID: 738). Based on the three ISES items, an overall ISES variable was created through latent class analysis (LCA) with the R package “poLCA”. According to the Akaike Information Criterion (AIC) and item-response probabilities, three latent classes were identified, representing high, moderate, and low ISES, respectively. In the three-latent class solution, the G^2^ is 1569, AIC is 2191578, Bayesian Information Criterion (BIC) is 2191952, and the mean posterior probabilities of all latent classes were above 0.70 (**Table S1**). Latent class 1 was characterized by high-level average household income, high education qualifications, and employment, which could be defined as “high ISES”; latent class 2 was characterized by low-level average household income, low education qualifications, and unemployment, which could be defined as “low ISES”; and latent class 3 was characterized by medium-level average household income, high and medium education qualification, and employment, which could be defined as “moderate ISES”.

**Calculation of partial PAR**

To quantify the separate contributions of ISES and neighborhood socioeconomic status (NSES) to incident dementia, we calculated partial population-attributable risk (PAR) percentages of incident dementia for lower ISES and NSES using the previously well-established method of multivariable-adjusted PAR with the SAS macro of PAR%.^1^ In an evaluation of a preventive intervention in a multifactorial disease setting, the interest is in the percentage of cases associated with the exposures to be modified, when other risk factors, possibly non-modifiable, exist but do not change as a result of the intervention. The partial PAR was proposed to estimate this quantity. Under the assumption of no interaction of the index exposure effects with the background risk factors, the PAR is formulated as:

$$PAR=1-\frac{\sum_{s=1}^{T} {p.}_{t}{RR}_{2t}}{\sum_{s=1}^{S} \sum_{t=1}^{T} p_{st}{RR}_{1s}{RR}_{2t}}$$

The details of the equation have been described previously.^1^

**Analyses of longitudinal associations of ISES and NSES with changes in cognitive function and brain structure**

In the UK Biobank, participants were invited to attend follow-up assessments, while about 20% of the population responded. There were three follow-up assessments for cognitive function. The scores of visual memory, processing speed, and verbal/numeric reasoning during follow-up were z-standardized according to the mean and standard deviation of baseline values. For associations of ISES and NSES with changes in visual memory, processing speed, and verbal/numeric reasoning scores, we used linear mixed effects models with follow-up time (in years) as the time scale. The fixed effect included the SES variable, follow-up time, and their interaction. For prospective memory, we used logistic regression models to estimate the associations of ISES and NSES with incident worse prospective memory (defined as “wrong”) during follow-up, among those who had good prospective memory at baseline. For the volume of white matter hyperintensity (WMH), there was one follow-up assessment. The volume of WMH during follow-up was z-standardized according to the mean and standard deviation of baseline values. We calculated the absolute changes in WMH volume between the follow-up and baseline assessment, and then used general linear regression models to estimate the associations of ISES and NSES with the volume of WMH change.


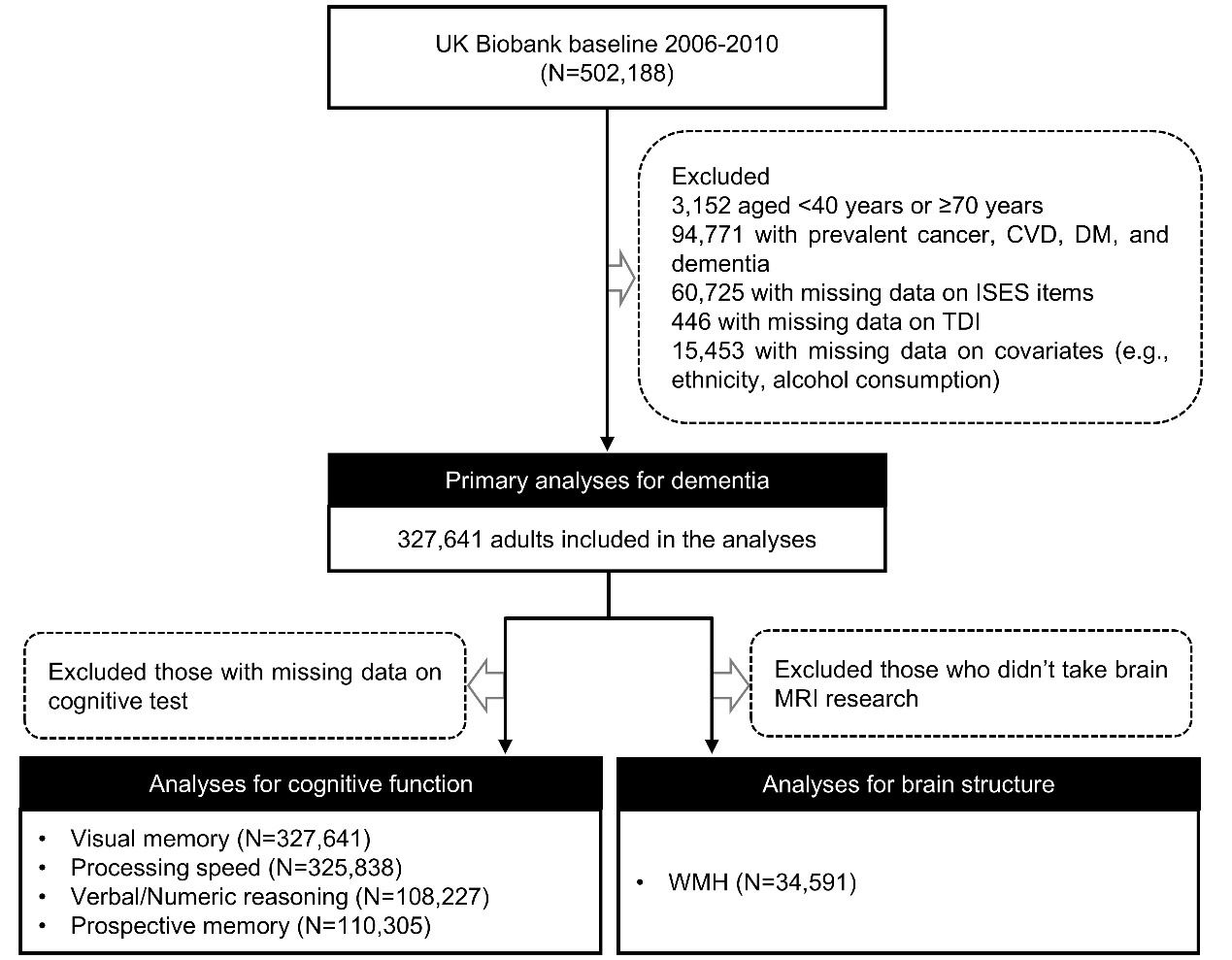


# Figure S1. Flow chart of the sample selection.

Abbreviations: CVD, cardiovascular disease; DM, diabetes mellitus; ISES, individual socioeconomic status; TDI, Townsend deprivation index; MRI, magnetic resonance imaging; WMH, white matter hyperintensity.


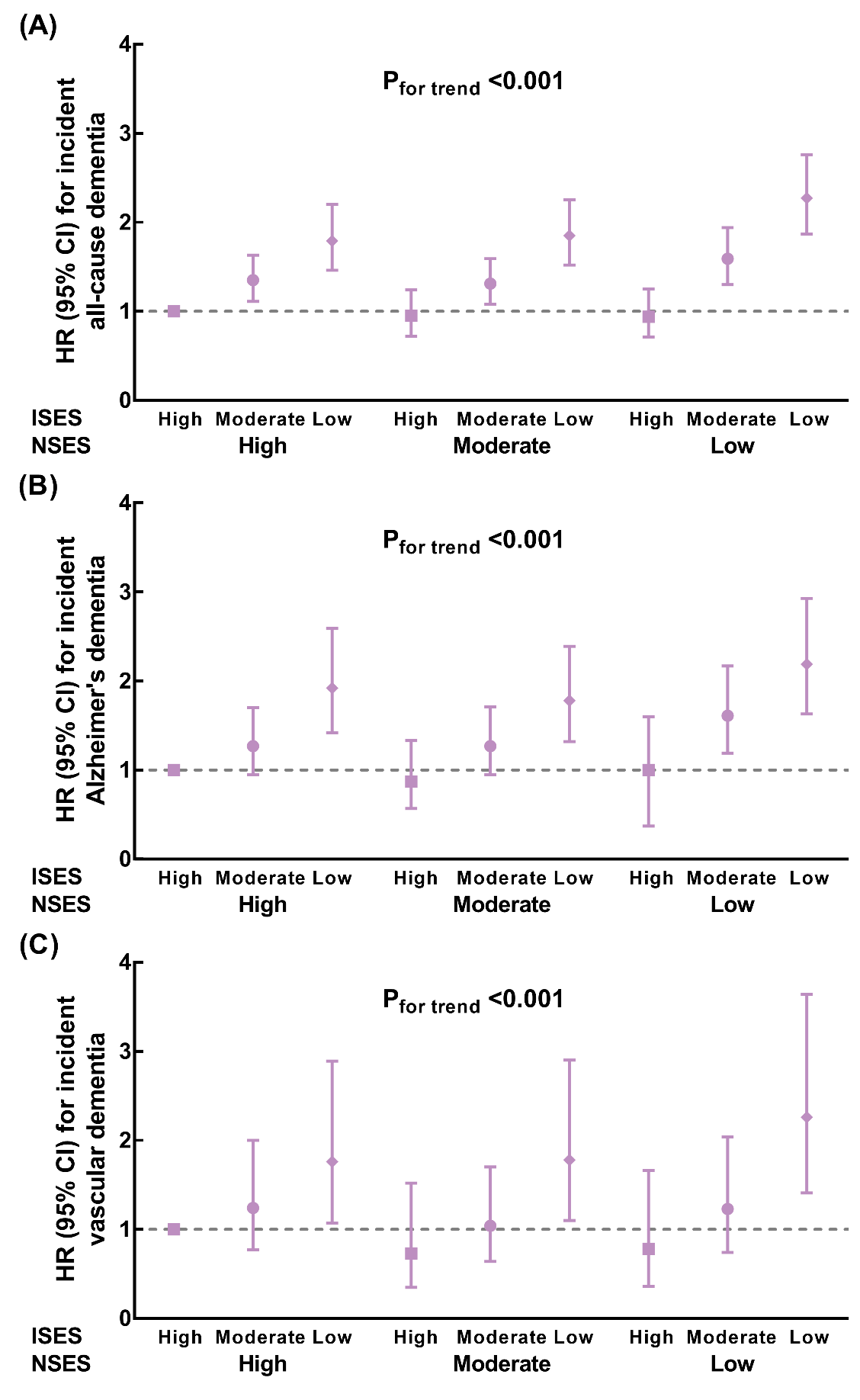


# Figure S2. Joint associations of ISES and NSES with the risk of dementia (N=327,641).

Abbreviations: ISES, individual socioeconomic status; NSES, neighborhood socioeconomic status; HR, hazard ratio; CI, confidence interval. The models were adjusted for age, gender, ethnicity, family history of Alzheimer’s dementia, apolipoprotein E genotypes, smoking status, alcohol consumption, healthy diet, regular exercise, sleep duration, body mass index, waist circumference, systolic blood pressure, and diastolic blood pressure.

**Table S1. Mean posterior probabilities, prevalence of latent classes, and item-related proportion of each latent class.**

| **Item** | **Latent class 1** | **Latent class 2** | **Latent class 3** |
| --- | --- | --- | --- |
| **Mean posterior probability** | 0.80 | 0.93 | 0.76 |
| **Prevalence** | 0.22 | 0.24 | 0.54 |
| **Educational attainment** |  |  |  |
| Equivalent to less than high school diploma | <0.01 | 0.33 | 0.07 |
| Other professional qualifications | 0.03 | 0.05 | 0.06 |
| NVQ or HND or HNC or equivalent | 0.02 | 0.08 | 0.08 |
| CSEs or equivalent | 0.01 | 0.07 | 0.08 |
| O levels/GCSEs or equivalent | 0.08 | 0.22 | 0.28 |
| A levels/AS levels or equivalent | 0.13 | 0.09 | 0.14 |
| College or university degree | 0.74 | 0.16 | 0.30 |
| **Occupational status** |  |  |  |
| Unemployed | 0.04 | 0.14 | 0.05 |
| Employed | 0.96 | 0.86 | 0.98 |
| **Income level** |  |  |  |
| Less than £18,000 | <0.01 | 0.68 | <0.01 |
| £18,000 to £30,999 | 0.02 | 0.25 | 0.38 |
| £31,000 to £51,999 | 0.23 | 0.05 | 0.44 |
| £52,000 to £100,000 | 0.52 | 0.01 | 0.19 |
| Greater than £100,000 | 0.22 | 0.01 | <0.01 |

Abbreviations: NVQ, National Vocational Qualification; HND, Higher National Diploma; HNC, Higher National Certificate; CSE, Certificate of Secondary Education; GCSE, General Certificate of Secondary Education; AS, Advanced Subsidiary.

#

# Table S2. Basic characteristics of the study participants in total and by SES profiles subgroups.^*^

| **Variables** | **Total (N=327,641)** | **Balanced-high SES subpopulation**  **(N=155,592)** | **Low income-less educated subpopulation (N=91,030)** | **Low income-NSES subpopulation (N=61,317)** | **Low SES subpopulation (N=19,702)** | **P value^†^** |
| --- | --- | --- | --- | --- | --- | --- |
| **Age in years** | 56.4 (49.1, 62.4) | 54.5 (48.0, 60.8) | 61.3 (54.9, 65.2) | 54.6 (47.3, 61.7) | 53.3 (47.2, 58.2) | <0.001 |
| **Gender** |  |  |  |  |  | <0.001 |
| Female | 176,024 (53.7) | 79,242 (50.9) | 50,210 (55.2) | 34,444 (56.2) | 12,128 (61.6) |  |
| Male | 151,617 (46.3) | 76,350 (49.1) | 40,820 (44.8) | 26,873 (43.8) | 7,574 (38.4) |  |
| **Ethnicity** |  |  |  |  |  | <0.001 |
| White | 313,315 (95.6) | 150,979 (97.0) | 88,860 (97.6) | 55,230 (90.1) | 18,246 (92.6) |  |
| Mixed | 1,920 (0.6) | 738 (0.5) | 324 (0.4) | 673 (1.1) | 185 (0.9) |  |
| South Asian | 4,630 (1.4) | 1,776 (1.1) | 793 (0.9) | 1,591 (2.6) | 470 (2.4) |  |
| Black | 4,364 (1.3) | 874 (0.6) | 572 (0.6) | 2,505 (4.1) | 413 (2.1) |  |
| Chinese | 980 (0.3) | 440 (0.3) | 172 (0.2) | 280 (0.5) | 88 (0.4) |  |
| Others | 2,432 (0.7) | 785 (0.5) | 309 (0.3) | 1,038 (1.7) | 300 (1.5) |  |
| **APOE** |  |  |  |  |  | 0.600 |
| Non e4-carrier | 234,419 (71.5) | 111,215 (71.5) | 65,165 (71.6) | 43,867 (71.5) | 14,172 (71.9) |  |
| e4-carrier | 93,222 (28.5) | 44,377 (28.5) | 25,865 (28.4) | 17,450 (28.5) | 5,530 (28.1) |  |
| **Family history of Alzheimer’s disease** |  |  |  |  |  | <0.001 |
| No | 289,176 (88.3) | 137,570 (88.4) | 79,474 (87.3) | 54,502 (88.9) | 17,630 (89.5) |  |
| Yes | 38,465 (11.7) | 18,022 (11.6) | 11,556 (12.7) | 6,815 (11.1) | 2,072 (10.5) |  |
| **Smoking status** |  |  |  |  |  | <0.001 |
| Non-smoker | 184,727 (56.4) | 96,245 (61.9) | 47,749 (52.5) | 30,882 (50.4) | 9,851 (50.0) |  |
| Ever-smoker | 109,202 (33.3) | 48,624 (31.3) | 33,938 (37.3) | 20,725 (33.8) | 5,915 (30.0) |  |
| Current smoker | 33,712 (10.3) | 10,723 (6.9) | 9,343 (10.3) | 9,710 (15.8) | 3,936 (20.0) |  |
| **Alcohol consumption** |  |  |  |  |  | <0.001 |
| Never or special occasions only | 53,805 (16.4) | 16,851 (10.8) | 17,555 (19.3) | 13,834 (22.6) | 5,565 (28.2) |  |
| One to three times per month | 36,304 (11.1) | 15,255 (9.8) | 10,774 (11.8) | 7,901 (12.9) | 2,374 (12.0) |  |
| One to four times per week | 166,985 (51.0) | 84,558 (54.3) | 46,204 (50.8) | 27,770 (45.3) | 8,453 (42.9) |  |
| Daily or almost daily | 70,547 (21.5) | 38,928 (25.0) | 16,497 (18.1) | 11,812 (19.3) | 3,310 (16.8) |  |
| **Variables** | **Total (N=327,641)** | **Balanced-high SES subpopulation**  **(N=155,592)** | **Low income-less educated subpopulation (N=91,030)** | **Low income-NSES subpopulation (N=61,317)** | **Low SES subpopulation (N=19,702)** | **P value^†^** |
| **Healthy diet** |  |  |  |  |  | <0.001 |
| No | 66,726 (20.4) | 28,582 (18.4) | 18,862 (20.7) | 14,103 (23.0) | 5,179 (26.3) |  |
| Yes | 260,915 (79.6) | 127,010 (81.6) | 72,168 (79.3) | 47,214 (77.0) | 14,523 (73.7) |  |
| **Regular exercise** |  |  |  |  |  | <0.001 |
| No | 141,760 (43.3) | 67,228 (43.2) | 37,850 (41.6) | 27,148 (44.3) | 9,534 (48.4) |  |
| Yes | 185,881 (56.7) | 88,364 (56.8) | 53,180 (58.4) | 34,169 (55.7) | 10,168 (51.6) |  |
| **Sleep duration in hours** | 7.0 (7.0, 8.0) | 7.0 (7.0, 8.0) | 7.0 (7.0, 8.0) | 7.0 (6.0, 8.0) | 7.0 (6.0, 8.0) | <0.001 |
| **BMI in kg/m^2^** | 26.4 (23.9, 29.4) | 26.1 (23.7, 28.8) | 26.9 (24.4, 29.8) | 26.6 (23.9, 29.9) | 26.5 (23.6, 30.3) | <0.001 |
| **WC in cm** | 89.0 (80.0, 98.0) | 88.0 (79.0, 97.0) | 90.0 (81.0, 99.0) | 89.0 (80.0, 98.0) | 88.2 (78.0, 99.0) | <0.001 |
| **SBP in mmHg** | 135.5 (124.0, 148.5) | 134.0 (123.0, 146.5) | 139.5 (128.0, 153.0) | 133.5 (122.5, 147.0) | 132.5 (121.0, 145.5) | <0.001 |
| **DBP in mmHg** | 82.0 (75.5, 89.0) | 81.5 (75.0, 88.5) | 82.5 (76.0, 89.5) | 82.0 (75.0, 89.0) | 82.0 (75.0, 89.0) | <0.001 |
| **ISES subgroups** |  |  |  |  |  | <0.001 |
| Low | 80,256 (24.5) | 3,948 (2.5) | 43,455 (47.7) | 19,113 (31.2) | 13,740 (69.7) |  |
| Moderate | 176,795 (54.0) | 90,516 (58.2) | 47,575 (52.3) | 36,391 (59.3) | 2,313 (11.7) |  |
| High | 70,590 (21.5) | 61,128 (39.3) | 0 (0.0) | 5,813 (9.5) | 3,649 (18.5) |  |
| **Educational attainment** |  |  |  |  |  | <0.001 |
| Equivalent to less than high school diploma | 41,708 (12.7) | 0 (0.0) | 32,870 (36.1) | 4,592 (7.5) | 4,246 (21.6) |  |
| Other professional qualifications | 16,077 (4.9) | 473 (0.3) | 13,239 (14.5) | 1,660 (2.7) | 705 (3.6) |  |
| NVQ or HND or HNC or equivalent | 21,072 (6.4) | 2,925 (1.9) | 13,093 (14.4) | 3,877 (6.3) | 1,177 (6.0) |  |
| CSEs or equivalent | 18,292 (5.6) | 3,948 (2.5) | 8,030 (8.8) | 4,650 (7.6) | 1,664 (8.4) |  |
| O levels/GCSEs or equivalent | 69,808 (21.3) | 28,457 (18.3) | 21,438 (23.6) | 15,482 (25.2) | 4,431 (22.5) |  |
| A levels/AS levels or equivalent | 39,146 (11.9) | 26,364 (16.9) | 2,360 (2.6) | 8,072 (13.2) | 2,350 (11.9) |  |
| College or university degree | 121,538 (37.1) | 93,425 (60.0) |  | 22,984 (37.5) | 5,129 (26.0) |  |
| **Variables** | **Total (N=327,641)** | **Balanced-high SES subpopulation**  **(N=155,592)** | **Low income-less educated subpopulation (N=91,030)** | **Low income-NSES subpopulation (N=61,317)** | **Low SES subpopulation (N=19,702)** | **P value^†^** |
| **Occupational status** |  |  |  |  |  | <0.001 |
| Unemployed | 19,702 (6.0) | 0 (0.0) | 0 (0.0) | 0 (0.0) | 19,702 (100.0)） |  |
| Employed | 307,939 (94.0) | 155,592 (100) | 91,030 (100) | 61,317 (100) | 0 (0.0) |  |
| **Income level** |  |  |  |  |  | <0.001 |
| Less than £18,000 | 64,043 (19.5) | 3,948 (2.5) | 32,743 (36.0) | 17,871 (29.1) | 9,481 (48.1) |  |
| £18,000 to £30,999 | 81,293 (24.8) | 20,614 (13.2) | 36,765 (40.4) | 20,194 (32.9) | 3,720 (18.9) |  |
| £31,000 to £51,999 | 89,726 (27.4) | 54,613 (35.1) | 16,575 (18.2) | 15,576 (25.4) | 2,962 (15.0) |  |
| £52,000 to £100,000 | 72,929 (22.3) | 59,572 (38.3) | 4,669 (5.1) | 6,391 (10.4) | 2,297 (11.7) |  |
| Greater than £100,000 | 19,650 (6.0) | 16,845 (10.8) | 278 (0.3) | 1,285 (2.1) | 1,242 (6.3) |  |
| **NSES subgroups** |  |  |  |  |  | <0.001 |
| Low | 109,117 (33.3) | 20,693 (13.3) | 18,494 (20.3) | 60,294 (98.3) | 9,636 (48.9) |  |
| Moderate | 109,422 (33.4) | 62,954 (40.5) | 40,067 (44.0) | 1,023 (1.7) | 5,378 (27.3) |  |
| High | 109,102 (33.3) | 71,945 (46.2) | 32,469 (35.7) | 0 (0.0) | 4,688 (23.8) |  |
| **TDI** | -2.3 (-3.7, 0.3) | -3.1 (-4.1, -1.8) | -2.6 (-3.7, -1.2) | 2.7 (1.2, 4.4) | -0.9 (-3.1, 2.6) | <0.001 |

Abbreviations: SES, socioeconomic status; APOE, apolipoprotein E; BMI, body mass index; WC, waist circumference; SBP, systolic blood pressure; DBP, diastolic blood pressure; ISES, individual socioeconomic status; NVQ, National Vocational Qualification; HND, Higher National Diploma; HNC, Higher National Certificate; CSE, Certificate of Secondary Education; GCSE, General Certificate of Secondary Education; AS, Advanced Subsidiary; NSES, neighborhood socioeconomic status; TDI, Townsend deprivation index.

^*^ The data are expressed as medians and inter-quartile range (IQR) for continuous variables and numbers and percentages for categorical variables.

^†^ The P values were generated using the Kruskal-Wallis test and Chi-square test for continuous and categorical variables, respectively.

# Table S3. Basic characteristics of the study participants in total and by ISES subgroups.^*^

| **Variables** | **Total (N=327,641)** | **High ISES (N=70,590)** | **Moderate ISES (N=176,795)** | **Low ISES (N=80,256)** | **P value**^†^ |
| --- | --- | --- | --- | --- | --- |
| **Age in years** | 56.4 (49.1, 62.4) | 52.3 (46.7, 57.9) | 56.1 (48.9, 62.1) | 61.2 (54.2, 65.3) | <0.001 |
| **Gender** |  |  |  |  | <0.001 |
| Female | 176,024 (53.7) | 34,729 (49.2) | 93,921 (53.1) | 47,374 (59.0) |  |
| Male | 151,617 (46.3) | 35,861 (50.8) | 82,874 (46.9) | 32,882 (41.0) |  |
| **Ethnicity** |  |  |  |  | <0.001 |
| White | 313,315 (95.6) | 67,864 (96.1) | 169,731 (96.0) | 75,720 (94.3) |  |
| Mixed | 1,920 (0.6) | 483 (0.7) | 940 (0.5) | 497 (0.6) |  |
| South Asian | 4,630 (1.4) | 1,044 (1.5) | 2,136 (1.2) | 1,450 (1.8) |  |
| Black | 4,364 (1.3) | 479 (0.7) | 2,405 (1.4) | 1,480 (1.8) |  |
| Chinese | 980 (0.3) | 238 (0.3) | 481 (0.3) | 261 (0.3) |  |
| Others | 2,432 (0.7) | 482 (0.7) | 1,102 (0.6) | 848 (1.1) |  |
| **APOE** |  |  |  |  | 0.820 |
| Non e4-carrier | 234,419 (71.5) | 50,533 (71.6) | 126,413 (71.5) | 57,473 (71.6) |  |
| e4-carrier | 93,222 (28.5) | 20,057 (28.4) | 50,382 (28.5) | 22,783 (28.4) |  |
| **Family history of Alzheimer’s disease** |  |  |  |  | <0.001 |
| No | 289,176 (88.3) | 62,883 (89.1) | 156,229 (88.4) | 70,064 (87.3) |  |
| Yes | 38,465 (11.7) | 7,707 (10.9) | 20,566 (11.6) | 10,192 (12.7) |  |
| **Smoking status** |  |  |  |  | <0.001 |
| Non-smoker | 184,727 (56.4) | 44,464 (63.0) | 100,627 (56.9) | 39,636 (49.4) |  |
| Ever-smoker | 109,202 (33.3) | 21,569 (30.6) | 59,188 (33.5) | 28,445 (35.4) |  |
| Current smoker | 33,712 (10.3) | 4,557 (6.5) | 16,980 (9.6) | 12,175 (15.2) |  |
| **Alcohol consumption** |  |  |  |  | <0.001 |
| Never or special occasions only | 53,805 (16.4) | 5,995 (8.5) | 25,819 (14.6) | 21,991 (27.4) |  |
| One to three times per month | 36,304 (11.1) | 5,612 (8.0) | 20,356 (11.5) | 10,336 (12.9) |  |
| One to four times per week | 166,985 (51.0) | 39,145 (55.5) | 92,385 (52.3) | 35,455 (44.2) |  |
| Daily or almost daily | 70,547 (21.5) | 19,838 (28.1) | 38,235 (21.6) | 12,474 (15.5) |  |

| **Variables** | **Total (N=327,641)** | **High ISES (N=70,590)** | **Moderate ISES (N=176,795)** | **Low ISES (N=80,256)** | **P value**^†^ |
| --- | --- | --- | --- | --- | --- |
| **Healthy diet** |  |  |  |  | <0.001 |
| No | 66,726 (20.4) | 12,383 (17.5) | 35,716 (20.2) | 18,627 (23.2) |  |
| Yes | 260,915 (79.6) | 58,207 (82.5) | 141,079 (79.8) | 61,629 (76.8) |  |
| **Regular exercise** |  |  |  |  | <0.001 |
| No | 141,760 (43.3) | 30,257 (42.9) | 75,729 (42.8) | 35,774 (44.6) |  |
| Yes | 185,881 (56.7) | 40,333 (57.1) | 101,066 (57.2) | 44,482 (55.4) |  |
| **Sleep duration in hours** | 7.0 (7.0, 8.0) | 7.0 (7.0, 8.0) | 7.0 (7.0, 8.0) | 7.0 (6.0, 8.0) | <0.001 |
| **BMI in kg/m^2^** | 26.4 (23.9, 29.4) | 25.8 (23.4, 28.5) | 26.5 (24.0, 29.4) | 26.9 (24.3, 30.1) | <0.001 |
| **WC in cm** | 89.0 (80.0, 98.0) | 88.0 (78.0, 96.0) | 89.0 (80.0, 98.0) | 90.0 (81.0, 99.0) | <0.001 |
| **SBP in mmHg** | 135.5 (124.0, 148.5) | 131.5 (121.0, 143.5) | 135.5 (124.0, 148.5) | 139.0 (126.5, 152.5) | <0.001 |
| **DBP in mmHg** | 82.0 (75.5, 89.0) | 81.0 (74.5, 88.0) | 82.0 (75.5, 89.0) | 82.5 (76.0, 89.5) | <0.001 |
| **Educational attainment** |  |  |  |  | <0.001 |
| Equivalent to less than high school diploma | 41,708 (12.7) | 0 (0.0) | 7,701 (4.4) | 34,007 (42.4) |  |
| Other professional qualifications | 16,077 (4.9) | 646 (0.9) | 12,081 (6.8) | 3,350 (4.2) |  |
| NVQ or HND or HNC or equivalent | 21,072 (6.4) | 424 (0.6) | 15,126 (8.6) | 5,522 (6.9) |  |
| CSEs or equivalent | 18,292 (5.6) | 216 (0.3) | 13,486 (7.6) | 4,590 (5.7) |  |
| O levels/GCSEs or equivalent | 69,808 (21.3) | 1,719 (2.4) | 52,793 (29.9) | 15,296 (19.1) |  |
| A levels/AS levels or equivalent | 39,146 (11.9) | 12,023 (17.0) | 21,096 (11.9) | 6,027 (7.5) |  |
| College or university degree | 121,538 (37.1) | 55,562 (78.7) | 54,512 (30.8) | 11,464 (14.3) |  |
| **Occupational status** |  |  |  |  | <0.001 |
| Unemployed | 19,702 (6.0) | 3,649 (5.2) | 2,313 (1.3) | 13,740 (17.1) |  |
| Employed | 307,939 (94.0) | 66,941 (94.8) | 17,4482 (98.7) | 66,516 (82.9) |  |
| **Income level** |  |  |  |  | <0.001 |
| Less than £18,000 | 64,043 (19.5) | 0 (0.0) | 0 (0.0) | 64,043 (79.8) |  |
| £18,000 to £30,999 | 81,293 (24.8) | 0 (0.0) | 65,904 (37.3) | 15,389 (19.2) |  |
| £31,000 to £51,999 | 89,726 (27.4) | 966 (1.4) | 88,440 (50.0) | 320 (0.4) |  |
| £52,000 to £100,000 | 72,929 (22.3) | 50,329 (71.3) | 22,451 (12.7) | 149 (0.2) |  |
| Greater than £100,000 | 19,650 (6.0) | 19,295 (27.3) | 0 (0.0) | 355 (0.4) |  |
| **Variables** | **Total (N=327,641)** | **High ISES (N=70,590)** | **Moderate ISES (N=176,795)** | **Low ISES (N=80,256)** | **P value**^†^ |
| **NSES subgroups** |  |  |  |  | <0.001 |
| Low | 109,117 (33.3) | 18,680 (26.5) | 52,442 (29.7) | 37,995 (47.3) |  |
| Moderate | 109,422 (33.4) | 23,152 (32.8) | 61,680 (34.9) | 24,590 (30.6) |  |
| High | 109,102 (33.3) | 28,758 (40.7) | 62,673 (35.4) | 17,671 (22.0) |  |
| **TDI** | -2.3 (-3.7, 0.3) | -2.7 (-4.0, 0.6) | -2.4 (-3.8, 0.2) | -1.1 (-3.0, 2.1) | <0.001 |

Abbreviations: ISES, individual socioeconomic status; APOE, apolipoprotein E; BMI, body mass index; WC, waist circumference; SBP, systolic blood pressure; DBP, diastolic blood pressure; NVQ, National Vocational Qualification; HND, Higher National Diploma; HNC, Higher National Certificate; CSE, Certificate of Secondary Education; GCSE, General Certificate of Secondary Education; AS, Advanced Subsidiary; NSES, neighborhood socioeconomic status; TDI, Townsend deprivation index.

^*^ The data are expressed as medians and inter-quartile range (IQR) for continuous variables and numbers and percentages for categorical variables.

^†^ The P values were generated using the Kruskal-Wallis test and Chi-square test for continuous and categorical variables, respectively.

# Table S4. Basic characteristics of the study participants in total and by NSES subgroups.^*^

| **Variables** | **Total (N=327,641)** | **High NSES (N=109,102)** | **Moderate NSES (N=109,422)** | **Low NSES (N=109,117)** | **P value**^†^ |
| --- | --- | --- | --- | --- | --- |
| **Age in years** | 56.4 (49.1, 62.4) | 57.4 (50.2, 62.9) | 56.9 (49.5, 62.6) | 54.9 (47.8, 61.7) | <0.001 |
| **Gender** |  |  |  |  | <0.001 |
| Female | 176,024 (53.7) | 57,710 (52.9) | 59,255 (54.2) | 59,059 (54.1) |  |
| Male | 151,617 (46.3) | 51,392 (47.1) | 50,167 (45.8) | 50,058 (45.9) |  |
| **Ethnicity** |  |  |  |  | <0.001 |
| White | 313,315 (95.6) | 107,022 (98.1) | 106,272 (97.1) | 100,021 (91.7) |  |
| Mixed | 1,920 (0.6) | 357 (0.3) | 503 (0.5) | 1,060 (1.0) |  |
| South Asian | 4,630 (1.4) | 821 (0.8) | 1,200 (1.1) | 2,609 (2.4) |  |
| Black | 4,364 (1.3) | 262 (0.2) | 683 (0.6) | 3,419 (3.1) |  |
| Chinese | 980 (0.3) | 261 (0.2) | 277 (0.3) | 442 (0.4) |  |
| Others | 2,432 (0.7) | 379 (0.3) | 487 (0.4) | 1,566 (1.4) |  |
| **APOE** |  |  |  |  | 0.040 |
| Non e4-carrier | 234,419 (71.5) | 77,756 (71.3) | 78,403 (71.7) | 78,260 (71.7) |  |
| e4-carrier | 93,222 (28.5) | 31,346 (28.7) | 31,019 (28.3) | 30,857 (28.3) |  |
| **Family history of Alzheimer’s disease** |  |  |  |  | <0.001 |
| No | 289,176 (88.3) | 95,989 (88.0) | 96,384 (88.1) | 96,803 (88.7) |  |
| Yes | 38,465 (11.7) | 13,113 (12.0) | 13,038 (11.9) | 12,314 (11.3) |  |
| **Smoking status** |  |  |  |  | <0.001 |
| Non-smoker | 184,727 (56.4) | 66,793 (61.2) | 63,115 (57.7) | 54,819 (50.2) |  |
| Ever-smoker | 109,202 (33.3) | 35,138 (32.2) | 36,780 (33.6) | 37,284 (34.2) |  |
| Current smoker | 33,712 (10.3) | 7,171 (6.6) | 9,527 (8.7) | 17,014 (15.6) |  |
| **Alcohol consumption** |  |  |  |  | <0.001 |
| Never or special occasions only | 53,805 (16.4) | 13,691 (12.5) | 16,494 (15.1) | 23,620 (21.6) |  |
| One to three times per month | 36,304 (11.1) | 11,184 (10.3) | 12,177 (11.1) | 12,943 (11.9) |  |
| One to four times per week | 166,985 (51.0) | 59,361 (54.4) | 56,938 (52.0) | 50,686 (46.5) |  |
| Daily or almost daily | 70,547 (21.5) | 24,866 (22.8) | 23,813 (21.8) | 21,868 (20.0) |  |
| **Variables** | **Total (N=327,641)** | **High NSES (N=109,102)** | **Moderate NSES (N=109,422)** | **Low NSES (N=109,117)** | **P value**^†^ |
| **Healthy diet** |  |  |  |  | <0.001 |
| No | 66,726 (20.4) | 19,999 (18.3) | 21,268 (19.4) | 25,459 (23.3) |  |
| Yes | 260,915 (79.6) | 89,103 (81.7) | 88,154 (80.6) | 83,658 (76.7) |  |
| **Regular exercise** |  |  |  |  | <0.001 |
| No | 141,760 (43.3) | 46,211 (42.4) | 46,662 (42.6) | 48,887 (44.8) |  |
| Yes | 185,881 (56.7) | 62,891 (57.6) | 62,760 (57.4) | 60,230 (55.2) |  |
| **Sleep duration in hours** | 7.0 (7.0, 8.0) | 7.0 (7.0, 8.0) | 7.0 (7.0, 8.0) | 7.0 (6.0, 8.0) | <0.001 |
| **BMI in kg/m^2^** | 26.4 (23.9, 29.4) | 26.2 (23.9, 29.0) | 26.4 (24.0, 29.3) | 26.7 (24.0, 29.9) | <0.001 |
| **WC in cm** | 89.0 (80.0, 98.0) | 88.0 (79.0, 97.0) | 89.0 (80.0, 97.0) | 89.0 (80.0, 98.0) | <0.001 |
| **SBP in mmHg** | 135.5 (124.0, 148.5) | 136.0 (124.5, 149.0) | 135.5 (124.0, 148.5) | 134.0 (123.0, 147.0) | <0.001 |
| **DBP in mmHg** | 82.0 (75.5, 89.0) | 82.0 (75.5, 89.0) | 82.0 (75.5, 89.0) | 82.0 (75.0, 89.0) | <0.001 |
| **ISES subgroups** |  |  |  |  | <0.001 |
| Low | 80,256 (24.5) | 17,671 (16.2) | 24,590 (22.5) | 37,995 (34.8) |  |
| Moderate | 176,795 (54.0) | 62,673 (57.4) | 61,680 (56.4) | 52,442 (48.1) |  |
| High | 70,590 (21.5) | 28,758 (26.4) | 23,152 (21.2) | 18,680 (17.1) |  |
| **Educational attainment** |  |  |  |  | <0.001 |
| Equivalent to less than high school diploma | 41,708 (12.7) | 10,118 (9.3) | 13,285 (12.1) | 18,305 (16.8) |  |
| Other professional qualifications | 16,077 (4.9) | 5,760 (5.3) | 5,583 (5.1) | 4,734 (4.3) |  |
| NVQ or HND or HNC or equivalent | 21,072 (6.4) | 6,614 (6.1) | 7,089 (6.5) | 7,369 (6.8) |  |
| CSEs or equivalent | 18,292 (5.6) | 4,969 (4.6) | 6,191 (5.7) | 7,132 (6.5) |  |
| O levels/GCSEs or equivalent | 69,808 (21.3) | 24,469 (22.4) | 24,399 (22.3) | 20,940 (19.2) |  |
| A levels/AS levels or equivalent | 39,146 (11.9) | 14,464 (13.3) | 13,358 (12.2) | 11,324 (10.4) |  |
| College or university degree | 121,538 (37.1) | 42,708 (39.1) | 39,517 (36.1) | 39,313 (36.0) |  |
| **Occupational status** |  |  |  |  | <0.001 |
| Unemployed | 19,702 (6.0) | 4,688 (4.3) | 5,378 (4.9) | 9,636 (8.8) |  |
| Employed | 307,939 (94.0) | 104,414 (95.7) | 104,044 (95.1) | 99,481 (91.2) |  |
| **Income level** |  |  |  |  | <0.001 |
| **Variables** | **Total (N=327,641)** | **High NSES (N=109,102)** | **Moderate NSES (N=109,422)** | **Low NSES (N=109,117)** | **P value**^†^ |
| Less than £18,000 | 64,043 (19.5) | 13,086 (12.0) | 18,968 (17.3) | 31,989 (29.3) |  |
| £18,000 to £30,999 | 81,293 (24.8) | 25,396 (23.3) | 27,790 (25.4) | 28,107 (25.8) |  |
| £31,000 to £51,999 | 89,726 (27.4) | 32,141 (29.5) | 31,508 (28.8) | 26,077 (23.9) |  |
| £52,000 to £100,000 | 72,929 (22.3) | 30,505 (28.0) | 25,036 (22.9) | 17,388 (15.9) |  |
| Greater than £100,000 | 19,650 (6.0) | 7,974 (7.3) | 6,120 (5.6) | 5,556 (5.1) |  |
| **TDI** | -2.3 (-3.7, 0.3) | -4.2 (-4.7, -3.7) | -2.3 (-2.8, -1.6) | 1.6 (0.3, 3.5) | <0.001 |

Abbreviations: NSES, neighborhood socioeconomic status; APOE, apolipoprotein E; BMI, body mass index; WC, waist circumference; SBP, systolic blood pressure; DBP, diastolic blood pressure; ISES, individual socioeconomic status; NVQ, National Vocational Qualification; HND, Higher National Diploma; HNC, Higher National Certificate; CSE, Certificate of Secondary Education; GCSE, General Certificate of Secondary Education; AS, Advanced Subsidiary; TDI, Townsend deprivation index.

^*^ The data are expressed as medians and inter-quartile range (IQR) for continuous variables and numbers and percentages for categorical variables.

^†^ The P values were generated using the Kruskal-Wallis test and Chi-square test for continuous and categorical variables, respectively

# Table S5. Associations of ISES, and NSES with the risk of dementia (N=327,641).

|  | **No. of events /**  **No. of participant** | **Model 1^*^** | **Model 2**^†^ | **Model 3^‡^** |
| --- | --- | --- | --- | --- |
|  |  | **HR (95% CI)** | **HR (95% CI)** | **HR (95% CI)** |
| **All-cause dementia** |  |  |  |  |
| High ISES | 289/70,590 | Ref. | Ref. | Ref. |
| Moderate ISES | 1,822/176,795 | 1.46 (1.29, 1.65) | 1.44 (1.27, 1.63) | 1.45 (1.28, 1.64) |
| Low ISES | 1,917/80,256 | 2.20 (1.93, 2.50) | 2.07 (1.82, 2.36) | 2.02 (1.77, 2.30) |
|  |  |  |  |  |
| High NSES | 1,309/109,102 | Ref. | Ref. | Ref. |
| Moderate NSES | 1,295/109,422 | 1.05 (0.97, 1.13) | 1.03 (0.95, 1.11) | 0.99 (0.92, 1.07) |
| Low NSES | 1,424/109,117 | 1.38 (1.28, 1.49) | 1.29 (1.20, 1.40) | 1.20 (1.11, 1.29) |
| **Alzheimer’s Dementia** |  |  |  |  |
| High ISES | 111/70,590 | Ref. | Ref. | Ref. |
| Moderate ISES | 823/176,795 | 1.61 (1.32, 1.96) | 1.58 (1.29, 1.93) | 1.59 (1.30, 1.94) |
| Low ISES | 911/80,256 | 2.43 (1.99, 2.98) | 2.31 (1.89, 2.84) | 2.28 (1.85, 2.79) |
|  |  |  |  |  |
| High NSES | 620/109,102 | Ref. | Ref. | Ref. |
| Moderate NSES | 594/109,422 | 1.01 (0.90, 1.13) | 1.00 (0.89, 1.12) | 0.96 (0.86, 1.07) |
| Low NSES | 631/109,117 | 1.31 (1.17, 1.46) | 1.25 (1.12, 1.40) | 1.15 (1.03, 1.29) |
| **Vascular Dementia** |  |  |  |  |
| High ISES | 41/70,590 | Ref. | Ref. | Ref. |
| Moderate ISES | 273/176,795 | 1.41 (1.01, 1.96) | 1.37 (0.99, 1.91) | 1.38 (0.99, 1.93) |
| Low ISES | 358/80,256 | 2.52 (1.80, 3.51) | 2.30 (1.65, 3.22) | 2.28 (1.63, 3.18) |
|  |  |  |  |  |
| High NSES | 225/109,102 | Ref. | Ref. | Ref. |
| Moderate NSES | 207/109,422 | 0.98 (0.81, 1.18) | 0.95 (0.78, 1.14) | 0.90 (0.74, 1.08) |
| Low NSES | 240/109,117 | 1.40 (1.16, 1.67) | 1.25 (1.04, 1.50) | 1.11 (0.93, 1.34) |

Abbreviations: ISES, individual socioeconomic status; NSES, neighborhood socioeconomic status; HR, hazard ratio; CI, confidence interval.

^*^ Model 1: adjusted for age, gender, ethnicity, family history of Alzheimer’s dementia, and APOE genotype.

^†^ Model 2: further adjusted for smoking status, alcohol consumption, healthy diet, regular exercise, sleep duration, body mass index, waist circumference, systolic blood pressure, and diastolic blood pressure based on model 1.

^‡^ Model 3: ISES and NSES were added into the model simultaneously based on model 2.

# Table S6. Associations of SES with the risk of dementia after further adjusting for genetic risk of Alzheimer’s dementia (N=327,086).

|  | **No. of events /**  **No. of participant** | **Model 1^*^** | **Model 2**^†^ |
| --- | --- | --- | --- |
|  |  | **HR (95% CI)** | **HR (95% CI)** |
| **All-cause dementia** |  |  |  |
| High ISES | 289/70,541 | Ref. | Ref. |
| Moderate ISES | 1,819/176,517 | 1.44 (1.27, 1.63) | 1.44 (1.27, 1.64) |
| Low ISES | 1,910/80,028 | 2.07 (1.81, 2.35) | 2.01 (1.77, 2.29) |
|  |  |  |  |
| High NSES | 1,306/108,957 | Ref. | Ref. |
| Moderate NSES | 1,295/109,244 | 1.03 (0.95, 1.11) | 0.99 (0.92, 1.07) |
| Low NSES | 1,417/108,885 | 1.29 (1.20, 1.40) | 1.20 (1.11, 1.29) |
| **Alzheimer’s Dementia** |  |  |  |
| High ISES | 111/70,541 | Ref. | Ref. |
| Moderate ISES | 823/176,517 | 1.58 (1.29, 1.93) | 1.59 (1.30, 1.94) |
| Low ISES | 910/80,028 | 2.31 (1.88, 2.83) | 2.27 (1.85, 2.78) |
|  |  |  |  |
| High NSES | 620/108,957 | Ref. | Ref. |
| Moderate NSES | 594/109,244 | 1.00 (0.89, 1.12) | 0.96 (0.86, 0.08) |
| Low NSES | 630/108,885 | 1.25 (1.12, 1.40) | 1.15 (1.03, 1.29) |
| **Vascular Dementia** |  |  |  |
| High ISES | 41/70,541 | Ref. | Ref. |
| Moderate ISES | 273/17,6517 | 1.37 (0.98, 1.91) | 1.38 (0.99, 1.92) |
| Low ISES | 356/80,028 | 2.29 (1.64, 3.20) | 2.26 (1.62, 3.16) |
|  |  |  |  |
| High NSES | 225/108,957 | Ref. | Ref. |
| Moderate NSES | 207/109,244 | 0.95 (0.79, 1.15) | 0.90 (0.75, 1.09) |
| Low NSES | 238/108,885 | 1.25 (1.04, 1.50) | 1.11 (0.92, 1.34) |

Abbreviations: SES, socioeconomic status; HR, hazard ratio; CI, confidence interval; ISES, individual socioeconomic status; NSES, neighborhood socioeconomic status.

**^*^** Model 1: adjusted for age, gender, ethnicity, family history of Alzheimer's disease, apolipoprotein E genotypes, smoking status, alcohol consumption, healthy diet, regular exercise, sleep duration, body mass index, waist circumference, systolic blood pressure, diastolic blood pressure, and genetic risk of Alzheimer’s dementia.

^†^ Model 2: ISES and NSES were added into the model simultaneously based on model 1.

# Table S7. Associations of SES with the risk of dementia when setting the end of follow-up at the occurrence of COVID-19 (N=327,641).

|  | **No. of events /**  **No. of participant** | **Model 1^*^** | **Model 2^†^** | **Model 3^‡^** |
| --- | --- | --- | --- | --- |
|  |  | **HR (95% CI)** | **HR (95% CI)** | **HR (95% CI)** |
| **All-cause dementia** |  |  |  |  |
| High ISES | 153/70,590 | Ref. | Ref. | Ref. |
| Moderate ISES | 910/176,795 | 1.41 (1.18, 1.67) | 1.40 (1.48, 1.67) | 1.41 (1.19, 1.68) |
| Low ISES | 1,013/80,256 | 2.24 (1.88, 2.68) | 2.16 (1.81, 2.59) | 2.11 (1.76, 2.52) |
|  |  |  |  |  |
| High NSES | 672/109,102 | Ref. | Ref. | Ref. |
| Moderate NSES | 656/109,422 | 1.03 (0.93, 1.15) | 1.01 (0.91, 1.13) | 0.97 (0.87, 1.08) |
| Low NSES | 748/109,117 | 1.40 (1.26, 1.55) | 1.31 (1.18, 1.45) | 1.19 (1.07, 1.32) |
| **Alzheimer’s Dementia** |  |  |  |  |
| High ISES | 57/70,590 | Ref. | Ref. | Ref. |
| Moderate ISES | 373/176,795 | 1.45 (1.10, 1.92) | 1.44 (1.09, 1.91) | 1.46 (1.10, 1.92) |
| Low ISES | 459/80,256 | 2.46 (1.85, 3.25) | 2.37 (1.78, 3.16) | 2.32 (1.75, 3.09) |
|  |  |  |  |  |
| High NSES | 296/109,102 | Ref. | Ref. | Ref. |
| Moderate NSES | 273/109,422 | 0.97 (0.83, 1.15) | 0.96 (0.81, 1.13) | 0.91 (0.77, 1.08) |
| Low NSES | 320/109,117 | 1.37 (1.17, 1.61) | 1.31 (1.12, 1.54) | 1.18 (1.00, 1.08) |
| **Vascular Dementia** |  |  |  |  |
| High ISES | 23/70,590 | Ref. | Ref. | Ref. |
| Moderate ISES | 128/176,795 | 1.19 (0.77, 1.85) | 1.19 (0.77, 1.86) | 1.21 (0.77, 1.88) |
| Low ISES | 186/80,256 | 2.36 (1.51, 3.69) | 2.28 (1.46, 3.56) | 2.26 (1.45, 3.53) |
|  |  |  |  |  |
| High NSES | 115/109,102 | Ref. | Ref. | Ref. |
| Moderate NSES | 97/109,422 | 0.90 (0.69, 1.18) | 0.87 (0.67, 1.14) | 0.82 (0.63, 1.07) |
| Low NSES | 125/109,117 | 1.41 (1.09, 1.82) | 1.27 (0.98, 1.63) | 1.10 (0.85, 1.42) |

Abbreviations: SES, socioeconomic status; HR, hazard ratio; CI, confidence interval; ISES, individual socioeconomic status; NSES, neighborhood socioeconomic status.

^*^ Model 1: adjusted for age, gender, ethnicity, family history of Alzheimer's disease, and apolipoprotein E genotypes.

^†^ Model 2: further adjusted for smoking status, alcohol consumption, healthy diet, regular exercise, sleep duration, body mass index, waist circumference, systolic blood pressure, and diastolic blood pressure based on model 1.

^‡^ Model 3: ISES and NSES were added into the model simultaneously based on model 2.

# Table S8. Associations of SES with the risk of dementia when excluding those who were followed up for less than 2 years.

|  | **No. of events /**  **No. of participant** | **Model 1**^*^ | **Model 2**^†^ | **Model 3**^‡^ |
| --- | --- | --- | --- | --- |
|  |  | **HR (95% CI)** | **HR (95% CI)** | **HR (95% CI)** |
| **All-cause dementia (N=326,712)** |  |  |  |  |
| High ISES | 286/70,475 | Ref. | Ref. | Ref. |
| Moderate ISES | 1,803/176,355 | 1.45 (1.28, 1.65) | 1.43 (1.26, 1.63) | 1.44 (1.27, 1.64) |
| Low ISES | 1,902/79,882 | 2.19 (1.93, 2.49) | 2.07 (1.81, 2.35) | 2.02 (1.77, 2.30) |
|  |  |  |  |  |
| High NSES | 1,301/108,855 | Ref. | Ref. | Ref. |
| Moderate NSES | 1,282/109,123 | 1.04 (0.96, 1.13) | 1.02 (0.95, 1.10) | 0.98 (0.91, 1.06) |
| Low NSES | 1,408/108,734 | 1.38 (1.28, 1.49) | 1.29 (1.19, 1.39) | 1.19 (1.10, 1.29) |
| **Alzheimer’s Dementia (N=326,740)** |  |  |  |  |
| High ISES | 111/70,478 | Ref. | Ref. | Ref. |
| Moderate ISES | 819/176,368 | 1.59 (1.31, 1.95) | 1.57 (1.29, 1.92) | 1.58 (1.30, 1.93) |
| Low ISES | 909/79,894 | 2.42 (1.98, 2.96) | 2.30 (1.88, 2.83) | 2.27 (1.85, 2.78) |
|  |  |  |  |  |
| High NSES | 619/108,862 | Ref. | Ref. | Ref. |
| Moderate NSES | 591/109,132 | 1.01 (0.90, 1.13) | 0.99 (0.89, 1.11) | 0.95 (0.85, 1.07) |
| Low NSES | 629/108,746 | 1.30 (1.17, 1.46) | 1.25 (1.12, 1.40) | 1.15 (1.03, 1.29) |
| **Vascular Dementia (N=326,744** |  |  |  |  |
| High ISES | 41/70,478 | Ref. | Ref. | Ref. |
| Moderate ISES | 272/176,372 | 1.40 (1.01, 1.95) | 1.37 (0.98, 1.91) | 1.38 (0.99, 1.92) |
| Low ISES | 356/79,894 | 2.51 (1.80, 3.51) | 2.29 (1.64, 3.21) | 2.27 (1.62, 3.17) |
|  |  |  |  |  |
| High NSES | 225/108,863 | Ref. | Ref. | Ref. |
| Moderate NSES | 205/109,134 | 0.97 (0.80, 1.17) | 0.94 (0.78, 1.13) | 0.89 (0.74, 1.07) |
| Low NSES | 239/108,747 | 1.39 (1.16, 1.67) | 1.24 (1.04, 1.50) | 1.11 (0.92, 1.34) |

Abbreviations: SES, socioeconomic status; HR, hazard ratio; CI, confidence interval; ISES, individual socioeconomic status; NSES, neighborhood socioeconomic status.

^*^ Model 1: adjusted for age, gender, ethnicity, family history of Alzheimer's disease, and apolipoprotein E genotypes.

^†^ Model 2: further adjusted for smoking status, alcohol consumption, healthy diet, regular exercise, sleep duration, body mass index, waist circumference, systolic blood pressure, and diastolic blood pressure based on model 1.

^‡^ Model 3: ISES and NSES were added into the model simultaneously based on model 2.

# **Table S9. Partial PAR percentages for incident dementia associated with low SES.***

|  | **PAR (%) (95% CI)** |
| --- | --- |
| **All-cause dementia** |  |
| ISES | 10.7 (8.8, 12.7) |
| NSES | 6.7 (4.2, 9.1) |
| **Alzheimer’s dementia** |  |
| ISES | 11.5 (8.6, 14.4) |
| NSES | 5.8 (2.2, 9.3) |
| **Vascular dementia** |  |
| ISES | 15.2 (10.3, 20.0) |
| NSES | 6.0 (-0.1, 12.1) |

Abbreviations: PAR, population-attributable risk; SES, socioeconomic status; CI, confidence interval; ISES, individual socioeconomic status; NSES, neighborhood socioeconomic status.

^*^ The models were adjusted for ISES, NSES, age, gender, ethnicity, family histories of Alzheimer’s dementia, apolipoprotein E genotypes, smoking status, alcohol consumption, healthy diet, regular exercise, sleep duration, body mass index, waist circumference, systolic blood pressure, and diastolic blood pressure.

# Table S10. Associations of ISES with the risk of dementia stratified by NSES (N=327,641).^*^

|  | | **No. of events / No. of participant** | **HR (95% CI)** |
| --- | --- | --- | --- |
| **All-cause dementia** | | | |
| High NSES | High ISES | 124/28,758 | Ref. |
|  | Moderate ISES | 741/62,673 | 1.28 (1.05, 1.56) |
|  | Low ISES | 444/17,671 | 1.65 (1.33, 2.04) |
| Moderate NSES | High ISES | 90/23,152 | Ref. |
|  | Moderate ISES | 605/61,680 | 1.39 (1.11, 1.74) |
|  | Low ISES | 600/24,590 | 1.98 (1.58, 2.50) |
| Low NSES | High ISES | 75/18,680 | Ref. |
|  | Moderate ISES | 476/52,442 | 1.71 (1.33, 2.18) |
|  | Low ISES | 873/37,995 | 2.50 (1.95, 3.20) |
| **Alzheimer’s Dementia** | | | |
| High NSES | High ISES | 54/28,758 | Ref. |
|  | Moderate ISES | 331/62,673 | 1.23 (0.91, 1.65) |
|  | Low ISES | 235/17,671 | 1.81 (1.32, 2.48) |
| Moderate NSES | High ISES | 36/23,152 | Ref. |
|  | Moderate ISES | 275/61,680 | 1.47 (1.03, 2.09) |
|  | Low ISES | 283/24,590 | 2.06 (1.44, 2.95) |
| Low NSES | High ISES | 21/18,680 | Ref. |
|  | Moderate ISES | 217/52,442 | 2.72 (1.74, 4.27) |
|  | Low ISES | 393/37,995 | 3.82 (2.43, 6.01) |
| **Vascular Dementia** | | | |
| High NSES | High ISES | 20/28,758 | Ref. |
|  | Moderate ISES | 123/62,673 | 1.16 (0.72, 1.87) |
|  | Low ISES | 82/17,671 | 1.62 (0.98, 2.68) |
| Moderate NSES | High ISES | 11/23,152 | Ref. |
|  | Moderate ISES | 86/61,680 | 1.47 (0.78, 2.77) |
|  | Low ISES | 110/24,590 | 2.52 (1.32, 4.82) |
| Low NSES | High ISES | 10/18,680 | Ref. |
|  | Moderate ISES | 64/52,442 | 1.56 (0.90, 3.05) |
|  | Low ISES | 166/37,995 | 2.86 (1.48, 5.51) |

Abbreviations: ISES, individual socioeconomic status; NSES, neighborhood socioeconomic status; HR, hazard ratio; CI, confidence interval.

^*^ The models were adjusted for age, gender, ethnicity, family history of Alzheimer’s dementia, apolipoprotein E genotypes, smoking status, alcohol consumption, healthy diet, regular exercise, sleep duration, body mass index, waist circumference, systolic blood pressure, and diastolic blood pressure.

# **Table S11. Associations of ISES with the risk of dementia stratified by NSES after further adjusting for genetic risk of Alzheimer’s dementia (N=327,086).^*^**

|  | | **No. of events /**  **No. of participant** | **HR (95% CI)** |
| --- | --- | --- | --- |
| **All-cause dementia** |  |  |  |
| High NSES | High ISES | 124/28,738 | Ref. |
|  | Moderate ISES | 739/62,582 | 1.28 (1.05, 1.56) |
|  | Low ISES | 443/17,637 | 1.65 (1.33, 2.04) |
| Moderate NSES | High ISES | 90/23,131 | Ref. |
|  | Moderate ISES | 605/61,590 | 1.40 (1.12, 1.75) |
|  | Low ISES | 600/24,523 | 2.00 (1.59, 2.52) |
| Low NSES | High ISES | 75/18,672 | Ref. |
|  | Moderate ISES | 475/52,345 | 1.68 (1.31, 2.15) |
|  | Low ISES | 867/37,868 | 2.46 (1.92, 3.15) |
| **Alzheimer’s Dementia** |  |  |  |
| High NSES | High ISES | 54/28,738 | Ref. |
|  | Moderate ISES | 331/62,582 | 1.23 (0.92, 1.66) |
|  | Low ISES | 235/17,637 | 1.82 (1.33, 2.49) |
| Moderate NSES | High ISES | 36/23,131 | Ref. |
|  | Moderate ISES | 275/61,590 | 1.48 (1.04, 2.10) |
|  | Low ISES | 283/24,523 | 2.07 (1.45, 2.97) |
| Low NSES | High ISES | 21/18,672 | Ref. |
|  | Moderate ISES | 217/52,345 | 2.66 (1.69, 4.17) |
|  | Low ISES | 392/37,868 | 3.72 (2.37, 5.85) |
| **Vascular Dementia** |  |  |  |
| High NSES | High ISES | 20/28,738 | Ref. |
|  | Moderate ISES | 123/62,582 | 1.62 (0.98, 2.69) |
|  | Low ISES | 82/17,637 | 1.17 (0.73, 1.88) |
| Moderate NSES | High ISES | 11/23,131 | Ref. |
|  | Moderate ISES | 86/61,590 | 1.48 (0.78, 4.83) |
|  | Low ISES | 110/24,523 | 2.52 (1.32, 4.83) |
| Low NSES | High ISES | 10/18,672 | Ref. |
|  | Moderate ISES | 64/52,345 | 1.54 (0.79, 3.02) |
|  | Low ISES | 164/37,868 | 2.81 (1.46, 5.43) |

Abbreviations: ISES, individual socioeconomic status; NSES, neighborhood socioeconomic status; HR, hazard ratio; CI, confidence interval.

^*^ The models were adjusted for age, gender, ethnicity, family history of Alzheimer's disease, apolipoprotein E genotypes, smoking status, alcohol consumption, healthy diet, regular exercise, sleep duration, body mass index, waist circumference, systolic blood pressure, diastolic blood pressure, and genetic risk of Alzheimer’s dementia. HRs for product term between ISES (low vs high) and NSES (low vs high) were 1.33 (95% CI 0.97, 1.81; P =0.073), 1.83 (95% CI 1.31, 3.12; P =0.025), and 1.63 (95% CI 0.73, 3.64; P =0.236), for the risks of all-cause dementia, Alzheimer’s dementia, and vascular dementia, respectively. The relative excess risks due to interaction (RERI) etween ISES (low vs high) and NSES (low vs high) were 0.52 (95% CI 0.20, 0.84), 0.64 (95% CI 0.212, 1.08), and 0.71 (95% CI -0.02, 1.43), for the risks of all-cause dementia, Alzheimer’s dementia, and vascular dementia, respectively.

# Table S12. Associations of ISES with the risk of dementia stratified by NSES when setting the end of follow-up at the occurrence of COVID-19 (N=327,641).^*^

|  | | **No. of events /**  **No. of participant** | **HR (95% CI)** |
| --- | --- | --- | --- |
| **All-cause dementia** |  |  |  |
| High NSES | High ISES | 74/28,758 | Ref. |
|  | Moderate ISES | 362/62,673 | 1.10 (0.85, 1.42) |
|  | Low ISES | 236/17,671 | 1.57 (1.19, 2.08) |
| Moderate NSES | High ISES | 41/23,152 | Ref. |
|  | Moderate ISES | 328/61,680 | 1.68 (1.51, 3.00) |
|  | Low ISES | 287/24,590 | 2.13 (1.51, 3.00) |
| Low NSES | High ISES | 38/18,680 | Ref. |
|  | Moderate ISES | 220/52,442 | 1.61 (1.14, 2.27) |
|  | Low ISES | 490/37,995 | 2.92 (2.06, 4.12) |
| **Alzheimer’s Dementia** |  |  |  |
| High NSES | High ISES | 31/28,758 | Ref. |
|  | Moderate ISES | 146/62,673 | 1.02 (0.69, 1.51) |
|  | Low ISES | 119/17,671 | 1.83 (1.20, 2.78) |
| Moderate NSES | High ISES | 16/23,152 | Ref. |
|  | Moderate ISES | 132/61,680 | 1.56 (0.92, 2.63) |
|  | Low ISES | 125/24,590 | 1.96 (1.14, 3.35) |
| Low NSES | High ISES | 10/18,680 | Ref. |
|  | Moderate ISES | 95/52,442 | 2.59 (1.35, 4.98) |
|  | Low ISES | 215/37,995 | 4.55 (2.37, 8.72) |
| **Vascular Dementia** |  |  |  |
| High NSES | High ISES | 11/28,758 | Ref. |
|  | Moderate ISES | 61/62,673 | 0.94 (0.50, 1.78) |
|  | Low ISES | 43/17,671 | 1.33 (0.67, 2.62) |
| Moderate NSES | High ISES | 5/23,152 | Ref. |
|  | Moderate ISES | 42/61,680 | 1.74 (0.70, 4.31) |
|  | Low ISES | 50/24,590 | 3.09 (1.24, 7.70) |
| Low NSES | High ISES | 7/18,680 | Ref. |
|  | Moderate ISES | 25/52,442 | 0.93 (0.40, 2.15) |
|  | Low ISES | 93/37,995 | 2.55 (1.15, 5.65) |

Abbreviations: ISES, individual socioeconomic status; NSES, neighborhood socioeconomic status; HR, hazard ratio; CI, confidence interval.

^*^ The models were adjusted for age, gender, ethnicity, family history of Alzheimer's disease, apolipoprotein E genotypes, smoking status, alcohol consumption, healthy diet, regular exercise, sleep duration, body mass index, waist circumference, systolic blood pressure, and diastolic blood pressure. HRs for product term between ISES (low vs high) and NSES (low vs high) were 1.61 (95% CI 1.06, 2.45; P =0.027), 2.36 (95% CI 1.11, 4.98; P =0.025), and 1.37 (95% CI 0.50, 3.80; P =0.603) for the risks of all-cause dementia, Alzheimer’s dementia, and vascular dementia, respectively. The relative excess risks due to interaction (RERI) between ISES (low vs high) and NSES (low vs high) were 0.71 (95% CI 0.31, 1.11), 0.85 (95% CI 0.30, 1.39), and 0.65 (95% CI -0.41, 1.72) for the risks of all-cause dementia, Alzheimer’s dementia, and vascular dementia, respectively.

# Table S13. Associations of ISES with the risk of dementia stratified by NSES when excluding those who were followed up for less than 2 years.^*^

|  | | **No. of events /**  **No. of participant** | **HR (95% CI)** |
| --- | --- | --- | --- |
| **All-cause dementia** |  |  |  |
| High NSES | High ISES | 123/28,716 | Ref. |
|  | Moderate ISES | 735/62,540 | 1.28 (1.05, 1.56) |
|  | Low ISES | 443/17,599 | 1.66 (1.34, 2.05) |
| Moderate NSES | High ISES | 89/23,110 | Ref. |
|  | Moderate ISES | 597/61,532 | 1.38 (1.11, 1.73) |
|  | Low ISES | 596/24,481 | 1.98 (1.57, 2.50) |
| Low NSES | High ISES | 74/18,649 | Ref. |
|  | Moderate ISES | 471/52,283 | 1.71 (1.33, 2.18) |
|  | Low ISES | 863/37,802 | 2.49 (1.94, 3.19) |
| **Alzheimer’s Dementia** |  |  |  |
| High NSES | High ISES | 54/28,717 | Ref. |
|  | Moderate ISES | 330/62,545 | 1.23 (0.91, 1.64) |
|  | Low ISES | 235/17,600 | 1.82 (1.33, 2.49) |
| Moderate NSES | High ISES | 36/23,111 | Ref. |
|  | Moderate ISES | 272/61,536 | 1.44 (1.02, 2.05) |
|  | Low ISES | 283/24,485 | 2.04 (1.42, 2.92) |
| Low NSES | High ISES | 21/18,650 | Ref. |
|  | Moderate ISES | 217/52,287 | 2.72 (1.74, 4.27) |
|  | Low ISES | 391/37,809 | 3.80 (2.42, 5.97) |
| **Vascular Dementia** |  |  |  |
| High NSES | High ISES | 20/28,717 | Ref. |
|  | Moderate ISES | 123/62,546 | 1.16 (0.72, 1.87) |
|  | Low ISES | 82/17,600 | 1.62 (0.98, 2.68) |
| Moderate NSES | High ISES | 11/23,111 | Ref. |
|  | Moderate ISES | 85/61,539 | 1.45 (0.77, 2.74) |
|  | Low ISES | 109/24,484 | 2.50 (1.30, 4.78) |
| Low NSES | High ISES | 10/18,650 | Ref. |
|  | Moderate ISES | 64/52,287 | 1.56 (0.80, 3.05) |
|  | Low ISES | 165/37,810 | 2.85 (1.48, 5.50) |

Abbreviations: ISES, individual socioeconomic status; NSES, neighborhood socioeconomic status; HR, hazard ratio; CI, confidence interval.

^*^ The models were adjusted for age, gender, ethnicity, family history of Alzheimer's disease, apolipoprotein E genotypes, smoking status, alcohol consumption, healthy diet, regular exercise, sleep duration, body mass index, waist circumference, systolic blood pressure, and diastolic blood pressure. HRs for product term between ISES (low vs high) and NSES (low vs high) were 1.35 (95% CI 0.998, 1.84; P =0.060), 1.88 (95% CI 1.11, 3.20; P =0.019), and 1.65 (95% CI 0.74, 3.68; P =0.225) for the risks of all-cause dementia, Alzheimer’s dementia, and vascular dementia, respectively. The relative excess risks due to interaction (RERI) between ISES (low vs high) and NSES (low vs high) were 0.53 (95% CI 0.21, 0.85), 0.66 (95% CI 0.23, 1.09), and 0.71 (95% CI -0.01, 1.44) for the risks of all-cause dementia, Alzheimer’s dementia, and vascular dementia, respectively.

# Table S14. Joint associations of ISES and NSES with the risk of dementia (N=327,641).^*^

|  | **No. of events / No. of participant** | **HR (95% CI)** |
| --- | --- | --- |
| **All-cause dementia** |  |  |
| High ISES & High NSES | 124/28,758 | Ref. |
| Moderate ISES & High NSES | 741/62,673 | 1.35 (1.11, 1.63) |
| Low ISES & High NSES | 444/17,671 | 1.79 (1.46, 2.20) |
| High ISES & Moderate NSES | 90/23,152 | 0.95 (0.72, 1.24) |
| Moderate ISES & Moderate NSES | 605/61,680 | 1.31 (1.08, 1.59) |
| Low ISES & Moderate NSES | 600/24,590 | 1.85 (1.52, 2.25) |
| High ISES & Low NSES | 75/18,680 | 0.94 (0.71, 1.25) |
| Moderate ISES & Low NSES | 476/52,442 | 1.59 (1.30, 1.94) |
| Low ISES & Low NSES | 873/37,995 | 2.27 (1.87, 2.76) |
| **Alzheimer’s Dementia** |  |  |
| High ISES & High NSES | 54/28,758 | Ref. |
| Moderate ISES & High NSES | 331/62,673 | 1.27 (0.95, 1.70) |
| Low ISES & High NSES | 235/17,671 | 1.92 (1.42, 2.59) |
| High ISES & Moderate NSES | 36/23,152 | 0.87 (0.57, 1.33) |
| Moderate ISES & Moderate NSES | 275/61,680 | 1.27 (0.95, 1.71) |
| Low ISES & Moderate NSES | 283/24,590 | 1.78 (1.32, 2.39) |
| High ISES & Low NSES | 21/18,680 | 0.60 (0.37, 1.00) |
| Moderate ISES & Low NSES | 217/52,442 | 1.61 (1.19, 2.17) |
| Low ISES & Low NSES | 393/37,995 | 2.19 (1.63, 2.93) |
| **Vascular Dementia** |  |  |
| High ISES & High NSES | 20/28,758 | Ref. |
| Moderate ISES & High NSES | 123/62,673 | 1.24 (0.77, 2.00) |
| Low ISES & High NSES | 82/17,671 | 1.76 (1.07, 2.89) |
| High ISES & Moderate NSES | 11/23,152 | 0.73 (0.35, 1.52) |
| Moderate ISES & Moderate NSES | 86/61,680 | 1.04 (0.64, 1.70) |
| Low ISES & Moderate NSES | 110/24,590 | 1.78 (1.10, 2.90) |
| High ISES & Low NSES | 10/18,680 | 0.78 (0.36, 1.66) |
| Moderate ISES & Low NSES | 64/52,442 | 1.23 (0.74, 2.04) |
| Low ISES & Low NSES | 166/37,995 | 2.26 (1.41, 3.64) |

Abbreviations: ISES, individual socioeconomic status; NSES, neighborhood socioeconomic status; HR, hazard ratio; CI, confidence interval.

^*^ The models were adjusted for age, gender, ethnicity, family histories of Alzheimer’s dementia, apolipoprotein E genotypes, smoking status, alcohol consumption, healthy diet, regular exercise, sleep duration, body mass index, waist circumference, systolic blood pressure, and diastolic blood pressure.

# Table S15. Joint associations of ISES and NSES with the risk of dementia after further adjusting for genetic risk of Alzheimer’s dementia (N=327,086).^*^

|  | **No. of events /**  **No. of participant** | **HR (95% CI)** |
| --- | --- | --- |
| **All-cause dementia** |  |  |
| High ISES & High NSES | 124/28,738 | Ref. |
| Moderate ISES & High NSES | 739/62,582 | 1.35 (1.11, 1.63) |
| Low ISES & High NSES | 443/17,637 | 1.79 (1.46, 2.19) |
| High ISES & Moderate NSES | 90/23,131 | 0.95 (0.72, 1.24) |
| Moderate ISES & Moderate NSES | 605/61,590 | 1.31 (1.08, 1.60) |
| Low ISES & Moderate NSES | 600/24,523 | 1.86 (1.52, 2.27) |
| High ISES & Low NSES | 75/18,672 | 0.95 (0.72, 1.27) |
| Moderate ISES & Low NSES | 475/52,345 | 1.58 (1.30, 1.93) |
| Low ISES & Low NSES | 867/37,868 | 2.27 (1.87, 2.75) |
| **Alzheimer’s Dementia** |  |  |
| High ISES & High NSES | 54/28,738 | Ref. |
| Moderate ISES & High NSES | 331/62,582 | 1.28 (0.96, 1.71) |
| Low ISES & High NSES | 235/17,637 | 1.92 (1.42, 2.60) |
| High ISES & Moderate NSES | 36/23,131 | 0.88 (0.58, 1.34) |
| Moderate ISES & Moderate NSES | 275/61,590 | 1.29 (0.96, 1.73) |
| Low ISES & Moderate NSES | 283/24,523 | 1.80 (1.33, 2.42) |
| High ISES & Low NSES | 21/18,672 | 0.62 (0.38, 1.03) |
| Moderate ISES & Low NSES | 217/52,345 | 1.61 (1.19, 2.17) |
| Low ISES & Low NSES | 392/37,868 | 2.19 (1.63, 2.93) |
| **Vascular Dementia** |  |  |
| High ISES & High NSES | 20/28,738 | Ref. |
| Moderate ISES & High NSES | 123/62,582 | 1.25 (0.78, 2.01) |
| Low ISES & High NSES | 82/17,637 | 1.76 (1.07, 2.88) |
| High ISES & Moderate NSES | 11/23,131 | 0.73 (0.35, 1.52) |
| Moderate ISES & Moderate NSES | 86/61,590 | 1.05 (0.64, 1.71) |
| Low ISES & Moderate NSES | 110/24,523 | 1.79 (1.10, 2.91) |
| High ISES & Low NSES | 10/18,672 | 0.79 (0.37, 1.69) |
| Moderate ISES & Low NSES | 64/52,345 | 1.23 (0.74, 2.04) |
| Low ISES & Low NSES | 164/37,868 | 2.25 (1.40, 3.62) |

Abbreviations: ISES, individual socioeconomic status; NSES, neighborhood socioeconomic status; HR, hazard ratio; CI, confidence interval.

^*^ The models were adjusted for age, gender, ethnicity, family history of Alzheimer's disease, apolipoprotein E genotypes, smoking status, alcohol consumption, healthy diet, regular exercise, sleep duration, body mass index, waist circumference, systolic blood pressure, diastolic blood pressure, and genetic risk of Alzheimer’s dementia.

# Table S16. Joint associations of ISES and NSES with the risk of dementia when setting the end of follow-up at the occurrence of COVID-19 (N=327,641).^*^

|  | **No. of events /**  **No. of participant** | **HR (95% CI)** |
| --- | --- | --- |
| **All-cause dementia** |  |  |
| High ISES & High NSES | 74/28,758 | Ref. |
| Moderate ISES & High NSES | 362/62,673 | 1.15 (0.89, 1.48) |
| Low ISES & High NSES | 236/17,671 | 1.70 (1.30, 2.22) |
| High ISES & Moderate NSES | 41/23,152 | 0.72 (0.49, 1.06) |
| Moderate ISES & Moderate NSES | 328/61,680 | 1.24 (0.96, 1.59) |
| Low ISES & Moderate NSES | 287/24,590 | 1.57 (1.21, 2.05) |
| High ISES & Low NSES | 38/18,680 | 0.81 (0.55, 1.20) |
| Moderate ISES & Low NSES | 220/52,442 | 1.27 (0.97, 1.65) |
| Low ISES & Low NSES | 490/37,995 | 2.22 (1.72, 2.85) |
| **Alzheimer’s Dementia** |  |  |
| High ISES & High NSES | 31/28,758 | Ref. |
| Moderate ISES & High NSES | 146/62,673 | 1.02 (0.69, 1.50) |
| Low ISES & High NSES | 119/17,671 | 1.79 (1.20, 2.68) |
| High ISES & Moderate NSES | 16/23,152 | 0.68 (0.37, 1.23) |
| Moderate ISES & Moderate NSES | 132/61,680 | 1.11 (0.75, 1.64) |
| Low ISES & Moderate NSES | 125/24,590 | 1.45 (0.97, 2.16) |
| High ISES & Low NSES | 10/18,680 | 0.51 (0.25, 1.04) |
| Moderate ISES & Low NSES | 95/52,442 | 1.27 (0.84, 1.89) |
| Low ISES & Low NSES | 215/37,995 | 2.15 (1.46, 3.16) |
| **Vascular Dementia** |  |  |
| High ISES & High NSES | 11/28,758 | Ref. |
| Moderate ISES & High NSES | 61/62,673 | 1.17 (0.61, 2.22) |
| Low ISES & High NSES | 43/17,671 | 1.81 (0.92, 3.54) |
| High ISES & Moderate NSES | 5/23,152 | 0.60 (0.21, 1.73) |
| Moderate ISES & Moderate NSES | 42/61,680 | 0.97 (0.50, 1.88) |
| Low ISES & Moderate NSES | 50/24,590 | 1.59 (0.81, 3.12) |
| High ISES & Low NSES | 7/18,680 | 0.98 (0.38, 2.53) |
| Moderate ISES & Low NSES | 25/52,442 | 0.91 (0.45, 1.86) |
| Low ISES & Low NSES | 93/37,995 | 2.45 (1.29, 4.65) |

Abbreviations: ISES, individual socioeconomic status; NSES, neighborhood socioeconomic status; HR, hazard ratio; CI, confidence interval.

^*^ The models were adjusted for age, gender, ethnicity, family history of Alzheimer's disease, apolipoprotein E genotypes, smoking status, alcohol consumption, healthy diet, regular exercise, sleep duration, body mass index, waist circumference, systolic blood pressure, and diastolic blood pressure.

# Table S17. Joint associations of ISES and NSES with the risk of dementia when excluding those who were followed up for less than 2 years.^*^

|  | **No. of events /**  **No. of participant** | **HR (95% CI)** |
| --- | --- | --- |
| **All-cause dementia (N=326,712)** |  |  |
| High ISES & High NSES | 123/28,716 | Ref. |
| Moderate ISES & High NSES | 735/62,540 | 1.34 (1.11, 1.63) |
| Low ISES & High NSES | 443/17,599 | 1.79 (1.46, 2.20) |
| High ISES & Moderate NSES | 89/23,110 | 0.94 (0.72, 1.24) |
| Moderate ISES & Moderate NSES | 597/61,532 | 1.30 (1.07, 1.58) |
| Low ISES & Moderate NSES | 596/24,481 | 1.84 (1.51, 2.24) |
| High ISES & Low NSES | 74/18,649 | 0.94 (0.70, 1.25) |
| Moderate ISES & Low NSES | 471/52,283 | 1.58 (1.29, 1.93) |
| Low ISES & Low NSES | 863/37,802 | 2.25 (1.86, 2.74) |
| **Alzheimer’s Dementia (N=326,740)** |  |  |
| High ISES & High NSES | 54/28,717 | Ref. |
| Moderate ISES & High NSES | 330/62,545 | 1.27 (0.95, 1.69) |
| Low ISES & High NSES | 235/17,600 | 1.91 (1.41, 2.58) |
| High ISES & Moderate NSES | 36/23,111 | 0.87 (0.57, 1.33) |
| Moderate ISES & Moderate NSES | 272/61,536 | 1.26 (0.94, 1.69) |
| Low ISES & Moderate NSES | 283/24,485 | 1.77 (1.32, 2.39) |
| High ISES & Low NSES | 21/18,650 | 0.60 (0.37, 1.00) |
| Moderate ISES & Low NSES | 217/52,287 | 1.60 (1.19, 2.16) |
| Low ISES & Low NSES | 391/37,809 | 2.17 (1.62, 2.91) |
| **Vascular Dementia (N=326,744)** |  |  |
| High ISES & High NSES | 20/28,717 | Ref. |
| Moderate ISES & High NSES | 123/62,546 | 1.25 (0.78, 2.01) |
| Low ISES & High NSES | 82/17,600 | 1.76 (1.07, 2.89) |
| High ISES & Moderate NSES | 11/23,111 | 0..73 (0.35, 1.52) |
| Moderate ISES & Moderate NSES | 85/61,539 | 1.03 (0.63, 1.69) |
| Low ISES & Moderate NSES | 109/24,484 | 1.77 (1.09, 2.88) |
| High ISES & Low NSES | 10/18,650 | 0.78 (0.36, 1.66) |
| Moderate ISES & Low NSES | 64/52,287 | 1.23 (0.74, 2.05) |
| Low ISES & Low NSES | 165/37,810 | 2.25 (1.40, 3.62) |

Abbreviations: ISES, individual socioeconomic status; NSES, neighborhood socioeconomic status; HR, hazard ratio; CI, confidence interval.

^*^ The models were adjusted for age, gender, ethnicity, family history of Alzheimer's disease, apolipoprotein E genotypes, smoking status, alcohol consumption, healthy diet, regular exercise, sleep duration, body mass index, waist circumference, systolic blood pressure, and diastolic blood pressure.

# Table S18. Joint associations of ISES and NSES with the risk of dementia stratified by age (N=327,641).^*^

|  | **No. of events /**  **No. of participant** | **HR (95% CI)** | **Interactive P value**^†^ |
| --- | --- | --- | --- |
| **All-cause dementia** |  |  | 0.110 |
| **<60 years** |  |  |  |
| High ISES & High NSES | 47/23,959 | Ref. |  |
| Moderate ISES & High NSES | 101/36,073 | 1.29 (0.91, 1.83) |  |
| Low ISES & High NSES | 28/5,696 | 1.82 (1.13, 2.92) |  |
| High ISES & Moderate NSES | 38/19,290 | 1.06 (0.69, 1.63) |  |
| Moderate ISES & Moderate NSES | 103/39,426 | 1.29 (0.91, 1.82) |  |
| Low ISES & Moderate NSES | 47/9,024 | 2.06 (1.36, 3.11) |  |
| High ISES & Low NSES | 26/15,306 | 1.00 (0.62, 1.62) |  |
| Moderate ISES & Low NSES | 120/38,575 | 1.73 (1.23, 2.43) |  |
| Low ISES & Low NSES | 138/19,909 | 3.15 (2.24, 4.44) |  |
| **≥60 years** |  |  |  |
| High ISES & High NSES | 77/4,799 | Ref. |  |
| Moderate ISES & High NSES | 640/26,600 | 1.31 (1.03, 1.66) |  |
| Low ISES & High NSES | 416/11,975 | 1.69 (1.32, 2.16) |  |
| High ISES & Moderate NSES | 52/3,862 | 0.88 (0.62, 1.25) |  |
| Moderate ISES & Moderate NSES | 502/22,254 | 1.26 (0.99, 1.61) |  |
| Low ISES & Moderate NSES | 553/15,566 | 1.73 (1.36, 2.20) |  |
| High ISES & Low NSES | 49/3,374 | 0.90 (0.63, 1.28) |  |
| Moderate ISES & Low NSES | 356/13,867 | 1.50 (1.17, 1.92) |  |
| Low ISES & Low NSES | 735/18,086 | 2.05 (1.62, 2.61) |  |
| **Alzheimer’s Dementia** |  |  | 0.330 |
| **<60 years** |  |  |  |
| High ISES & High NSES | 17/23,959 | Ref. |  |
| Moderate ISES & High NSES | 40/36,073 | 1.40 (0.79, 2.47) |  |
| Low ISES & High NSES | 9/5,696 | 1.51 (0.66, 3.43) |  |
| High ISES & Moderate NSES | 14/19,290 | 1.09 (0.54, 2.22) |  |
| Moderate ISES & Moderate NSES | 46/39,426 | 1.62 (0.93, 2.83) |  |
| Low ISES & Moderate NSES | 15/9,024 | 1.78 (0.88, 3.60) |  |
| High ISES & Low NSES | 7/15,306 | 0.77 (0.32, 1.84) |  |
| Moderate ISES & Low NSES | 39/38,575 | 1.71 (0.97, 3.01) |  |
| Low ISES & Low NSES | 52/19,909 | 3.69 (2.09, 6.51) |  |
| **≥60 years** |  |  |  |
| High ISES & High NSES | 37/4,799 | Ref. |  |
| Moderate ISES & High NSES | 291/26,600 | 1.19 (0.85, 1.68) |  |
| Low ISES & High NSES | 226/11,975 | 1.80 (1.27, 2.56) |  |
| High ISES & Moderate NSES | 22/3,862 | 0.77 (0.46, 1.31) |  |
| Moderate ISES & Moderate NSES | 229/22,254 | 1.16 (0.82, 1.65) |  |
| Low ISES & Moderate NSES | 268/15,566 | 1.66 (1.17, 2.35) |  |
| High ISES & Low NSES | 14/3,374 | 0.54 (0.29, 0.99) |  |
| Moderate ISES & Low NSES | 178/13,867 | 1.54 (1.08, 2.20) |  |
| Low ISES & Low NSES | 341/18,086 | 1.93 (1.37, 2.73) |  |
| **Vascular Dementia** |  |  | 0.510 |
| **<60 years** |  |  |  |
| High ISES & High NSES | 4/23,959 | Ref. |  |
| Moderate ISES & High NSES | 6/36,073 | 0.86 (0.24, 3.04) |  |
| Low ISES & High NSES | 2/5,696 | 1.43 (0.26, 7.93) |  |
| High ISES & Moderate NSES | 4/19,290 | 1.31 (0.33, 5.28) |  |
| Moderate ISES & Moderate NSES | 12/39,426 | 1.67 (0.54, 5.22) |  |
|  | **No. of events /**  **No. of participant** | **HR (95% CI)** | **Interactive P value**^†^ |
| Low ISES & Moderate NSES | 7/9,024 | 3.34 (0.97, 11.52) |  |
| High ISES & Low NSES | 3/15,306 | 1.40 (0.31, 6.34) |  |
| Moderate ISES & Low NSES | 14/38,575 | 2.26 (0.73, 6.94) |  |
| Low ISES & Low NSES | 14/19,909 | 3.45 (1.11, 10.70) |  |
| **≥60 years** |  |  |  |
| High ISES & High NSES | 16/4,799 | Ref. |  |
| Moderate ISES & High NSES | 117/26,600 | 1.16 (0.69, 1.96) |  |
| Low ISES & High NSES | 80/11,975 | 1.58 (0.92, 2.71) |  |
| High ISES & Moderate NSES | 7/3,862 | 0.58 (0.24, 1.41) |  |
| Moderate ISES & Moderate NSES | 74/22,254 | 0.91 (0.53, 1.55) |  |
| Low ISES & Moderate NSES | 103/15,566 | 1.55 (0.91, 2.64) |  |
| High ISES & Low NSES | 7/3,374 | 0.63 (0.26, 1.53) |  |
| Moderate ISES & Low NSES | 50/13,867 | 1.02 (0.58, 1.80 |  |
| Low ISES & Low NSES | 152/18,086 | 1.99 (1.18, 3.35) |  |

Abbreviations: SES, socioeconomic status; HR, hazard ratio; CI, confidence interval; PRS, polygenic risk score; ISES, individual socioeconomic status; NSES, neighborhood socioeconomic status.

^*^ The Models were adjusted for age, gender, ethnicity, family history of Alzheimer's disease, apolipoprotein E genotypes, smoking status, alcohol consumption, healthy diet, regular exercise, sleep duration, body mass index, waist circumference, systolic blood pressure, and diastolic blood pressure.

^†^ Interactive P value indicated the modifying effect of age on the associations of ISES and NSES with dementia among total participants.

# Table S19. Joint associations of ISES and NSES with the risk of dementia stratified by gender (N=327,641).^*^

|  | **No. of events /**  **No. of participant** | **HR (95% CI)** | **Interactive P value**^†^ |
| --- | --- | --- | --- |
| **All-cause dementia** |  |  | 0.360 |
| **Female** |  |  |  |
| High ISES & High NSES | 44/13,810 | Ref. |  |
| Moderate ISES & High NSES | 306/32,888 | 1.19 (0.86, 1.63) |  |
| Low ISES & High NSES | 252/11,012 | 1.64 (1.18, 2.27) |  |
| High ISES & Moderate NSES | 34/11,443 | 0.93 (0.59, 1.46) |  |
| Moderate ISES & Moderate NSES | 257/32,892 | 1.12 (0.82, 1.55) |  |
| Low ISES & Moderate NSES | 291/14,920 | 1.45 (1.05, 2.00) |  |
| High ISES & Low NSES | 32/9,476 | 0.99 (0.63, 1.56) |  |
| Moderate ISES & Low NSES | 213/28,141 | 1.39 (1.00, 1.92) |  |
| Low ISES & Low NSES | 448/21,442 | 1.94 (1.41, 2.67) |  |
| **Male** |  |  |  |
| High ISES & High NSES | 80/14,948 | Ref. |  |
| Moderate ISES & High NSES | 435/29,785 | 1.45 (1.14, 1.85) |  |
| Low ISES & High NSES | 192/6,659 | 1.82 (1.39, 2.37) |  |
| High ISES & Moderate NSES | 56/11,709 | 0.95 (0.67, 1.33) |  |
| Moderate ISES & Moderate NSES | 348/28,788 | 1.43 (1.12, 1.83) |  |
| Low ISES & Moderate NSES | 309/9,670 | 2.22 (1.72, 2.85) |  |
| High ISES & Low NSES | 43/9,204 | 0.90 (0.62, 1.30) |  |
| Moderate ISES & Low NSES | 263/24,301 | 1.72 (1.34, 2.21) |  |
| Low ISES & Low NSES | 445/16,553 | 2.49 (1.95, 3.18) |  |
| **Alzheimer’s Dementia** |  |  | 0.670 |
| **Female** |  |  |  |
| High ISES & High NSES | 21/13,810 | Ref. |  |
| Moderate ISES & High NSES | 146/32,888 | 1.08 (0.68, 1.71) |  |
| Low ISES & High NSES | 140/11,012 | 1.69 (1.06, 2.69) |  |
| High ISES & Moderate NSES | 18/11,443 | 1.03 (0.55, 1.93) |  |
| Moderate ISES & Moderate NSES | 122/32,892 | 1.03 (0.65, 1.65) |  |
| Low ISES & Moderate NSES | 150/14,920 | 1.41 (0.88, 2.24) |  |
| High ISES & Low NSES | 10/9,476 | 0.64 (0.30, 1.37) |  |
| Moderate ISES & Low NSES | 106/28,141 | 1.42 (0.88, 2.26) |  |
| Low ISES & Low NSES | 214/21,442 | 1.85 (1.17, 2.93) |  |
| **Male** |  |  |  |
| High ISES & High NSES | 33/14,948 | Ref. |  |
| Moderate ISES & High NSES | 185/29,785 | 1.42 (0.98, 2.07) |  |
| Low ISES & High NSES | 95/6,659 | 2.02 (1.35, 3.02) |  |
| High ISES & Moderate NSES | 18/11,709 | 0.75 (0.42, 1.32) |  |
| Moderate ISES & Moderate NSES | 153/28,788 | 1.47 (1.01, 2.15) |  |
| Low ISES & Moderate NSES | 133/9,670 | 2.15 (1.46, 3.17) |  |
| High ISES & Low NSES | 11/9,204 | 0.56 (0.28, 1.11) |  |
| Moderate ISES & Low NSES | 111/24,301 | 1.73 (1.17, 2.56) |  |
| Low ISES & Low NSES | 179/16,553 | 2.43 (1.66, 3.55) |  |
| **Vascular Dementia** |  |  | 0.490 |
| **Female** |  |  |  |
| High ISES & High NSES | 6/13,810 | Ref. |  |
| Moderate ISES & High NSES | 46/32,888 | 1.04 (0.44, 2.46) |  |
| Low ISES & High NSES | 48/11,012 | 1.61 (0.68, 3.82) |  |
| High ISES & Moderate NSES | 4/11,443 | 0.79 (0.22 2.80) |  |
| Moderate ISES & Moderate NSES | 30/32,892 | 0.77 (0.32, 1.85) |  |
|  | **No. of events /**  **No. of participant** | **HR (95% CI)** | **Interactive P value**^†^ |
| Low ISES & Moderate NSES | 48/14,920 | 1.22 (0.51, 2.90) |  |
| High ISES & Low NSES | 4/9,476 | 0.88 (0.25, 3.16) |  |
| Moderate ISES & Low NSES | 20/28,141 | 0.79 (0.31, 1.98) |  |
| Low ISES & Low NSES | 78/21,442 | 1.71 (0.73, 4.01) |  |
| **Male** |  |  |  |
| High ISES & High NSES | 14/14,948 | Ref. |  |
| Moderate ISES & High NSES | 77/29,785 | 1.35 (0.76, 2.39) |  |
| Low ISES & High NSES | 34/6,659 | 1.62 (0.86, 3.05) |  |
| High ISES & Moderate NSES | 7/11,709 | 0.69 (0.28, 1.70) |  |
| Moderate ISES & Moderate NSES | 56/28,788 | 1.21 (0.67, 2.19) |  |
| Low ISES & Moderate NSES | 62/9,670 | 2.23 (1.23, 4.02) |  |
| High ISES & Low NSES | 6/9,204 | 0.71 (0.27, 1.85) |  |
| Moderate ISES & Low NSES | 44/24,301 | 1.54 (0.84, 2.82) |  |
| Low ISES & Low NSES | 88/16,553 | 2.58 (1.45, 4.59) |  |

Abbreviations: SES, socioeconomic status; HR, hazard ratio; CI, confidence interval; PRS, polygenic risk score; ISES, individual socioeconomic status; NSES, neighborhood socioeconomic status.

^*^ The Models were adjusted for age, ethnicity, family history of Alzheimer's disease, apolipoprotein E genotypes, smoking status, alcohol consumption, healthy diet, regular exercise, sleep duration, body mass index, waist circumference, systolic blood pressure, and diastolic blood pressure.

^†^ Interactive P value indicated the modifying effect of gender on the associations of ISES and NSES with dementia among total participants.

# Table S20. Joint associations of ISES and NSES with the risk of dementia stratified by APOE genotypes (N=327,641).^*^

|  | **No. of events /**  **No. of participant** | **HR (95% CI)** | **Interactive P value**^†^ |
| --- | --- | --- | --- |
| **All-cause dementia** |  |  | 0.210 |
| **Non e4-carrier** |  |  |  |
| High ISES & High NSES | 60/20,461 | Ref. |  |
| Moderate ISES & High NSES | 338/44,732 | 1.39 (1.05, 1.83) |  |
| Low ISES & High NSES | 187/12,563 | 1.82 (1.36, 2.45) |  |
| High ISES & Moderate NSES | 45/16,605 | 0.97 (0.66, 1.43) |  |
| Moderate ISES & Moderate NSES | 259/44,190 | 1.25 (0.94, 1.65) |  |
| Low ISES & Moderate NSES | 272/17,608 | 1.98 (1.49, 2.63 |  |
| High ISES & Low NSES | 43/13,467 | 1.15 (0.77, 1.70) |  |
| Moderate ISES & Low NSES | 220/37,491 | 1.58 (1.19, 2.11) |  |
| Low ISES & Low NSES | 426/27,302 | 2.45 (1.86, 3.24) |  |
| **e4-carrier** |  |  |  |
| High ISES & High NSES | 64/8,297 | Ref. |  |
| Moderate ISES & High NSES | 403/17,941 | 1.31 (1.01, 1.71) |  |
| Low ISES & High NSES | 257/5,108 | 1.75 (1.32, 2.32) |  |
| High ISES & Moderate NSES | 45/6,547 | 0.92 (0.63, 1.35) |  |
| Moderate ISES & Moderate NSES | 346/17,490 | 1.35 (1.03, 1.76) |  |
| Low ISES & Moderate NSES | 328/6,982 | 1.73 (1.31, 2.28) |  |
| High ISES & Low NSES | 32/5,213 | 0.76 (0.50, 1.16) |  |
| Moderate ISES & Low NSES | 256/14,951 | 1.59 (1.21, 2.10) |  |
| Low ISES & Low NSES | 447/10,693 | 2.11 (1.61, 2.77) |  |
| **Alzheimer’s Dementia** |  |  | 0.520 |
| **Non e4-carrier** |  |  |  |
| High ISES & High NSES | 23/20,461 | Ref. |  |
| Moderate ISES & High NSES | 122/44,732 | 1.18 (0.76, 1.85) |  |
| Low ISES & High NSES | 81/12,563 | 1.76 (1.10, 2.83) |  |
| High ISES & Moderate NSES | 10/16,605 | 0.57 (0.27, 1.19) |  |
| Moderate ISES & Moderate NSES | 89/44,190 | 1.03 (0.65, 1.64) |  |
| Low ISES & Moderate NSES | 105/17,608 | 1.72 (1.09, 2.73) |  |
| High ISES & Low NSES | 9/13,467 | 0.63 (0.29, 1.36) |  |
| Moderate ISES & Low NSES | 82/37,491 | 1.47 (0.93, 2.34) |  |
| Low ISES & Low NSES | 162/27,302 | 2.20 (1.41, 3.45) |  |
| **e4-carrier** |  |  |  |
| High ISES & High NSES | 31/8,297 | Ref. |  |
| Moderate ISES & High NSES | 209/17,941 | 1.34 (0.92, 1.96) |  |
| Low ISES & High NSES | 154/5,108 | 2.03 (1.37, 3.00) |  |
| High ISES & Moderate NSES | 26/6,547 | 1.10 (0.66, 1.86) |  |
| Moderate ISES & Moderate NSES | 186/17,490 | 1.44 (0.98, 2.11) |  |
| Low ISES & Moderate NSES | 178/6,982 | 1.83 (1.24, 2.70) |  |
| High ISES & Low NSES | 12/5,213 | 0.59 (0.30, 1.15) |  |
| Moderate ISES & Low NSES | 135/14,951 | 1.71 (1.16, 2.53) |  |
| Low ISES & Low NSES | 231/10,693 | 2.18 (1.48, 3.20) |  |
| **Vascular Dementia** |  |  | 0.850 |
| **Non e4-carrier** |  |  |  |
| High ISES & High NSES | 11/20,461 | Ref. |  |
| Moderate ISES & High NSES | 62/44,732 | 1.22 (0.64, 2.32) |  |
| Low ISES & High NSES | 34/12,563 | 1.49 (0.75, 2.96) |  |
| High ISES & Moderate NSES | 7/16,605 | 0.83 (0.32, 2.14) |  |
| Moderate ISES & Moderate NSES | 45/44,190 | 1.05 (0.54, 2.03) |  |
|  | **No. of events /**  **No. of participant** | **HR (95% CI)** | **Interactive P value**^†^ |
| Low ISES & Moderate NSES | 52/17,608 | 1.69 (0.87, 3.27) |  |
| High ISES & Low NSES | 5/13,467 | 0.73 (0.25, 2.10) |  |
| Moderate ISES & Low NSES | 35/37,491 | 1.26 (0.64, 2.48) |  |
| Low ISES & Low NSES | 88/27,302 | 2.25 (1.19, 4.24) |  |
| **e4-carrier** |  |  |  |
| High ISES & High NSES | 9/8,297 | Ref. |  |
| Moderate ISES & High NSES | 61/17,941 | 1.27 (0.63, 2.57) |  |
| Low ISES & High NSES | 48/5,108 | 2.01 (0.97, 4.17) |  |
| High ISES & Moderate NSES | 4/6,547 | 0.59 (0.18, 1.93) |  |
| Moderate ISES & Moderate NSES | 41/17,490 | 1.04 (0.50, 2.14) |  |
| Low ISES & Moderate NSES | 58/6,982 | 1.87 (0.91, 3.86) |  |
| High ISES & Low NSES | 5/5,213 | 0.82 (0.28, 2.45) |  |
| Moderate ISES & Low NSES | 29/14,951 | 1.21 (0.57, 2.57) |  |
| Low ISES & Low NSES | 78/10,693 | 2.27 (1.11, 4.62) |  |

Abbreviations: ISES, individual socioeconomic status; NSES, neighborhood socioeconomic status; APOE, apolipoprotein E; HR, hazard ratio; CI, confidence interval; PRS, polygenic risk score.

^*^ The Models were adjusted for age, gender, ethnicity, family history of Alzheimer's disease, smoking status, alcohol consumption, healthy diet, regular exercise, sleep duration, body mass index, waist circumference, systolic blood pressure, and diastolic blood pressure.

^†^ Interactive P value indicated the modifying effect of APOE genotypes on the associations of ISES and NSES with dementia among total participants.

Table S21. Associations of SES profiles with the risk of dementia (N=327,641).*

|  | **No. of events / No. of participant** | **HR (95% CI)** |
| --- | --- | --- |
| **All-cause dementia** |  |  |
| Balanced-high SES subpopulation | 1,222/155,592 | Ref. |
| Low income-less educated subpopulation | 1,811/91,030 | 1.27 (1.18, 1.37) |
| Low income-NSES subpopulation | 772/61,317 | 1.41 (1.29, 1.55) |
| Low SES subpopulation | 223/19,702 | 2.32 (2.01, 2.68) |
| **Alzheimer’s Dementia** |  |  |
| Balanced-high SES subpopulation | 518/155,592 | Ref. |
| Low income-less educated subpopulation | 898/91,030 | 1.43 (1.28, 1.60) |
| Low income-NSES subpopulation | 344/61,317 | 1.49 (1.30, 1.72) |
| Low SES subpopulation | 85/19,702 | 2.27 (1.80, 2.87) |
| **Vascular Dementia** |  |  |
| Balanced-high SES subpopulation | 181/155,592 | Ref. |
| Low income-less educated subpopulation | 333/91,030 | 1.42 (1.18, 1.72) |
| Low income-NSES subpopulation | 122/61,317 | 1.44 (1.14, 1.82) |
| Low SES subpopulation | 36/19,702 | 2.76 (1.92, 3.95) |

Abbreviations: SES, socioeconomic status; HR, hazard ratio; CI, confidence interval; NSES, neighborhood socioeconomic status.

^*^ The models were adjusted for age, gender, ethnicity, family history of Alzheimer’s dementia, apolipoprotein E genotypes, smoking status, alcohol consumption, healthy diet, regular exercise, sleep duration, body mass index, waist circumference, systolic blood pressure, and diastolic blood pressure.

# Table S22. Associations of SES profiles with the risk of dementia after further adjusting for genetic risk of Alzheimer’s dementia (N=327,086).^*^

| **SES profiles** | **No. of events /**  **No. of participant** | **HR (95% CI)** |
| --- | --- | --- |
| **All-cause dementia** |  |  |
| Balanced-high SES subpopulation | 1,222/155,430 | Ref. |
| Low income-less educated subpopulation | 1,805/90,813 | 1.27 (1.18, 1.37) |
| Low income-NSES subpopulation | 769/61,188 | 1.42 (1.29, 1.55) |
| Low SES subpopulation | 222/19,655 | 2.31 (2.00, 2.67) |
| **Alzheimer’s Dementia** |  |  |
| Balanced-high SES subpopulation | 518/155,430 | Ref. |
| Low income-less educated subpopulation | 897/90,813 | 1.44 (1.28, 1.61) |
| Low income-NSES subpopulation | 344/61,188 | 1.50 (1.31, 1.73) |
| Low SES subpopulation | 85/19,655 | 2.25 (1.78, 2.84) |
| **Vascular Dementia** |  |  |
| Balanced-high SES subpopulation | 181/155,430 | Ref. |
| Low income-less educated subpopulation | 332/90,813 | 1.43 (1.18, 1.73) |
| Low income-NSES subpopulation | 122/61,188 | 1.45 (1.14, 1.83) |
| Low SES subpopulation | 35/19,655 | 2.68 (1.86, 3.86) |

Abbreviations: SES, socioeconomic status; HR, hazard ratio; CI, confidence interval; NSES, neighborhood socioeconomic status.

^*^ The models were adjusted for age, gender, ethnicity, family history of Alzheimer's disease, apolipoprotein E genotypes, smoking status, alcohol consumption, healthy diet, regular exercise, sleep duration, body mass index, waist circumference, systolic blood pressure, diastolic blood pressure, and genetic risk of Alzheimer’s dementia.

# Table S23. Associations of SES profiles with the risk of dementia when setting the end of follow-up at the occurrence of COVID-19 (N=327,641).^*^

|  | **No. of events /**  **No. of participant** | **HR (95% CI)** |
| --- | --- | --- |
| **All-cause dementia** |  |  |
| Balanced-high SES subpopulation | 622/155,592 | Ref. |
| Low income-less educated subpopulation | 925/91,030 | 1.30 (1.17, 1.45) |
| Low income-NSES subpopulation | 389/61,317 | 1.40 (1.23, 1.60) |
| Low SES subpopulation | 140/19,702 | 2.79 (1.32, 3.37) |
| **Alzheimer’s Dementia** |  |  |
| Balanced-high SES subpopulation | 236/155,592 | Ref. |
| Low income-less educated subpopulation | 444/91,030 | 1.59 (1.35, 1.87) |
| Low income-NSES subpopulation | 159/61,317 | 1.52 (1.24, 1.86) |
| Low SES subpopulation | 50/19,702 | 2.83 (2.07, 3.86) |
| **Vascular Dementia** |  |  |
| Balanced-high SES subpopulation | 89/155,592 | Ref. |
| Low income-less educated subpopulation | 166/91,030 | 1.49 (1.14, 1.94) |
| Low income-NSES subpopulation | 61/61,317 | 1.48 (1.06, 2.06) |
| Low SES subpopulation | 21/19,702 | 3.28 (2.04, 5.27) |

Abbreviations: SES, socioeconomic status; HR, hazard ratio; CI, confidence interval; NSES, neighborhood socioeconomic status.

^*^ The models were adjusted for age, gender, ethnicity, family history of Alzheimer's disease, apolipoprotein E genotypes, smoking status, alcohol consumption, healthy diet, regular exercise, sleep duration, body mass index, waist circumference, systolic blood pressure, and diastolic blood pressure.

# Table S24. Associations of SES profiles with the risk of dementia when excluding those who were followed up for less than 2 years.^*^

|  | **No. of events /**  **No. of participant** | **HR (95% CI)** |
| --- | --- | --- |
| **All-cause dementia** |  |  |
| Balanced-high SES subpopulation | 1,210/155,298 | Ref. |
| Low income-less educated subpopulation | 1,801/90,710 | 1.27 (1.18, 1.37) |
| Low income-NSES subpopulation | 763/61,094 | 1.41 (1.28, 1.55) |
| Low SES subpopulation | 217/19,610 | 2.29 (1.98, 2.65) |
| **Alzheimer’s Dementia** |  |  |
| Balanced-high SES subpopulation | 516/155,307 | Ref. |
| Low income-less educated subpopulation | 896/90,718 | 1.43 (1.28, 1.60) |
| Low income-NSES subpopulation | 343/61,101 | 1.50 (1.30, 1.72) |
| Low SES subpopulation | 84/19,614 | 2.26 (1.79, 2.86) |
| **Vascular Dementia** |  |  |
| Balanced-high SES subpopulation | 180/155,309 | Ref. |
| Low income-less educated subpopulation | 332/90,719 | 1.43 (1.18, 1.73) |
| Low income-NSES subpopulation | 121/61,101 | 1.43 (1.13, 1.81) |
| Low SES subpopulation | 36/19,615 | 2.76 (1.93, 3.96) |

Abbreviations: SES, socioeconomic status; HR, hazard ratio; CI, confidence interval; NSES, neighborhood socioeconomic status.

^*^ The models were adjusted for age, gender, ethnicity, family history of Alzheimer's disease, apolipoprotein E genotypes, smoking status, alcohol consumption, healthy diet, regular exercise, sleep duration, body mass index, waist circumference, systolic blood pressure, and diastolic blood pressure.

# Table S25. Associations of SES profiles with the risk of dementia stratified by age (N=327,641).^*^

| **SES profiles** | **No. of events /**  **No. of participant** | **HR (95% CI)** | **Interactive P value**^†^ |
| --- | --- | --- | --- |
| **All-cause dementia** |  |  | 0.006 |
| **<60 years** |  |  |  |
| Balanced-high SES subpopulation | 244/111,267 | Ref. |  |
| Low income-less educated subpopulation | 115/37,879 | 1.39 (1.12, 1.71) |  |
| Low income-NSES subpopulation | 134/41,399 | 1.59 (1.28, 1.97) |  |
| Low SES subpopulation | 125/16,713 | 3.22 (2.57, 4.03) |  |
| **≥60 years** |  |  |  |
| Balanced-high SES subpopulation | 978/44,325 | Ref. |  |
| Low income-less educated subpopulation | 1,666/53,151 | 1.23 (1.14, 1.34) |  |
| Low income-NSES subpopulation | 638/19,918 | 1.37 (1.23, 1.51) |  |
| Low SES subpopulation | 98/2,989 | 1.89 (1.54, 2.34) |  |
| **Alzheimer’s dementia** |  |  | 0.140 |
| **<60 years** |  |  |  |
| Balanced-high SES subpopulation | 91/111,267 | Ref. |  |
| Low income-less educated subpopulation | 68/37,879 | 1.77 (1.28, 2.44) |  |
| Low income-NSES subpopulation | 38/41,399 | 1.31 (0.89, 1.91) |  |
| Low SES subpopulation | 42/16,713 | 3.04 (2.06, 4.49) |  |
| **≥60 years** |  |  |  |
| Balanced-high SES subpopulation | 427/44,325 | Ref. |  |
| Low income-less educated subpopulation | 830/53,151 | 1.39 (1.23, 1.56) |  |
| Low income-NSES subpopulation | 306/19,918 | 1.51 (1.30, 1.76) |  |
| Low SES subpopulation | 43/2,989 | 1.96 (1.43, 2.69) |  |
| **Vascular dementia** |  |  | 0.490 |
| **<60 years** |  |  |  |
| Balanced-high SES subpopulation | 19/111,267 | Ref. |  |
| Low income-less educated subpopulation | 19/37,879 | 2.13 (1.10, 4.14) |  |
| Low income-NSES subpopulation | 14/41,399 | 2.14 (1.04, 4.40) |  |
| Low SES subpopulation | 14/16,713 | 4.70 (2.34, 9.46) |  |
| **≥60 years** |  |  |  |
| Balanced-high SES subpopulation | 162/44,325 | Ref. |  |
| Low income-less educated subpopulation | 314/53,151 | 1.35 (1.12, 1.64) |  |
| Low income-NSES subpopulation | 108/19,918 | 1.36 (1.06, 1.74) |  |
| Low SES subpopulation | 22/2,989 | 2.48 (1.59, 3.87) |  |

Abbreviations: SES, socioeconomic status; HR, hazard ratio; CI, confidence interval; NSES, neighborhood socioeconomic status.

^*^ The models were adjusted for age, gender, ethnicity, family history of Alzheimer's disease, apolipoprotein E genotypes, smoking status, alcohol consumption, healthy diet, regular exercise, sleep duration, body mass index, waist circumference, systolic blood pressure, and diastolic blood pressure.

^†^ Interactive P value indicated the modifying effect of age on the associations of SES profiles with dementia among total participants.

# Table S26. Associations of SES profiles with the risk of dementia stratified by gender (N=327,641).^*^

| **SES profiles** | **No. of events /**  **No. of participant** | **HR (95% CI)** | **Interactive P value**^†^ |
| --- | --- | --- | --- |
| **All-cause dementia** |  |  | 0.003 |
| **Female** |  |  |  |
| Balanced-high SES subpopulation | 479/79,242 | Ref. |  |
| Low income-less educated subpopulation | 891/50,210 | 1.28 (1.14, 1.43) |  |
| Low income-NSES subpopulation | 423/34,444 | 1.54 (1.35, 1.77) |  |
| Low SES subpopulation | 84/12,128 | 1.92 (1.52, 2.43) |  |
| **Male** |  |  |  |
| Balanced-high SES subpopulation | 743/76,350 | Ref. |  |
| Low income-less educated subpopulation | 920/40,820 | 1.27 (1.15, 1.41) |  |
| Low income-NSES subpopulation | 349/26,873 | 1.29 (1.13, 1.47) |  |
| Low SES subpopulation | 139/7,574 | 2.71 (2.25, 3.27) |  |
| **Alzheimer’s dementia** |  |  | 0.010 |
| **Female** |  |  |  |
| Balanced-high SES subpopulation | 212/79,242 | Ref. |  |
| Low income-less educated subpopulation | 473/50,210 | 1.50 (1.27, 1.78) |  |
| Low income-NSES subpopulation | 207/34,444 | 1.78 (1.46, 2.17) |  |
| Low SES subpopulation | 35/12,128 | 2.05 (1.43, 2.95) |  |
| **Male** |  |  |  |
| Balanced-high SES subpopulation | 306/76,350 | Ref. |  |
| Low income-less educated subpopulation | 425/40,820 | 1.39 (1.19, 1.62) |  |
| Low income-NSES subpopulation | 137/26,873 | 1.23 (1.00, 1.51) |  |
| Low SES subpopulation | 50/7,574 | 2.49 (1.84, 3.37) |  |
| **Vascular dementia** |  |  | 0.540 |
| **Female** |  |  |  |
| Balanced-high SES subpopulation | 59/79,242 | Ref. |  |
| Low income-less educated subpopulation | 158/50,210 | 1.54 (1.14, 2.09) |  |
| Low income-NSES subpopulation | 54/34,444 | 1.43 (0.98, 2.08) |  |
| Low SES subpopulation | 13/12,128 | 2.75 (1.52, 4.98) |  |
| **Male** |  |  |  |
| Balanced-high SES subpopulation | 122/76,350 | Ref. |  |
| Low income-less educated subpopulation | 175/40,820 | 1.36 (1.06, 1.74) |  |
| Low income-NSES subpopulation | 68/26,873 | 1.47 (1.09, 1.99) |  |
| Low SES subpopulation | 23/7,574 | 2.81 (1.78, 4.43) |  |

Abbreviations: SES, socioeconomic status; HR, hazard ratio; CI, confidence interval; NSES, neighborhood socioeconomic status.

^*^ The models were adjusted for age, ethnicity, family history of Alzheimer's disease, apolipoprotein E genotypes, smoking status, alcohol consumption, healthy diet, regular exercise, sleep duration, body mass index, waist circumference, systolic blood pressure, and diastolic blood pressure.

^†^ Interactive P value indicated the modifying effect of gender on the associations of SES profiles with dementia among total participants.

# Table S27. Associations of SES profiles with the risk of dementia stratified by APOE genotypes (N=327,641).^*^

| **SES profiles** | **No. of events /**  **No. of participant** | **HR (95% CI)** | **Interactive P value**^†^ |
| --- | --- | --- | --- |
| **All-cause dementia** |  |  | 0.110 |
| **Non e4-carrier** |  |  |  |
| Balanced-high SES subpopulation | 564/111,215 | Ref. |  |
| Low income-less educated subpopulation | 801/65,165 | 1.27 (1.14, 1.43) |  |
| Low income-NSES subpopulation | 370/43,867 | 1.46 (1.28, 1.67) |  |
| Low SES subpopulation | 115/14,172 | 2.33 (1.90, 2.86) |  |
| **e4-carrier** |  |  |  |
| Balanced-high SES subpopulation | 658/44,377 | Ref. |  |
| Low income-less educated subpopulation | 1,010/25,865 | 1.27 (1.14, 1.40) |  |
| Low income-NSES subpopulation | 402/17,450 | 1.37 (1.21, 1.56) |  |
| Low SES subpopulation | 108/5,530 | 2.30 (1.87, 2.83) |  |
| **Alzheimer’s dementia** |  |  | 0.430 |
| **Non e4-carrier** |  |  |  |
| Balanced-high SES subpopulation | 182/111,215 | Ref. |  |
| Low income-less educated subpopulation | 328/65,165 | 1.52 (1.26, 1.83) |  |
| Low income-NSES subpopulation | 136/43,867 | 1.66 (1.32, 2.08) |  |
| Low SES subpopulation | 37/14,172 | 2.60 (1.81, 3.73) |  |
| **e4-carrier** |  |  |  |
| Balanced-high SES subpopulation | 336/44,377 | Ref. |  |
| Low income-less educated subpopulation | 570/25,865 | 1.38 (1.20, 1.59) |  |
| Low income-NSES subpopulation | 208/17,450 | 1.41 (1.18, 1.68) |  |
| Low SES subpopulation | 48/5,530 | 2.08 (1.53, 2.82) |  |
| **Vascular dementia** |  |  | 0.120 |
| **Non e4-carrier** |  |  |  |
| Balanced-high SES subpopulation | 98/111,215 | Ref. |  |
| Low income-less educated subpopulation | 153/65,165 | 1.25 (0.96, 1.63) |  |
| Low income-NSES subpopulation | 70/43,867 | 1.50 (1.09, 2.05) |  |
| Low SES subpopulation | 18/14,172 | 2.33 (1.40, 3.88) |  |
| **e4-carrier** |  |  |  |
| Balanced-high SES subpopulation | 83/44,377 | Ref. |  |
| Low income-less educated subpopulation | 180/25,865 | 1.63 (1.24, 2.13) |  |
| Low income-NSES subpopulation | 52/17,450 | 1.36 (0.95, 1.94) |  |
| Low SES subpopulation | 18/5,530 | 3.32 (1.99, 5.52) |  |

Abbreviations: SES, socioeconomic status; APOE, apolipoprotein E; HR, hazard ratio; CI, confidence interval; NSES, neighborhood socioeconomic status.

^*^ The models were adjusted for age, gender, ethnicity, family history of Alzheimer's disease, smoking status, alcohol consumption, healthy diet, regular exercise, sleep duration, body mass index, waist circumference, systolic blood pressure, and diastolic blood pressure.

^†^ Interactive P value indicated the modifying effect of APOE genotypes on the associations of SES profiles with dementia among total participants.

# Table S28. Associations of SES with cognitive function.

| **SES** | **No. of participant** | **Model 1**^*^ | **Model 2^†^** | **Model 3^‡^** |
| --- | --- | --- | --- | --- |
|  |  | **β/OR (95% CI)** | **β/OR (95% CI)** | **β/OR (95% CI)** |
| **Visual memory (N=327,641)** | | | | |
| High ISES | 70,590 | Ref. | Ref. | Ref. |
| Moderate ISES | 176,795 | 0.08 (0.07, 0.09) | 0.08 (0.08, 0.09) | 0.08 (0.07, 0.09) |
| Low ISES | 80,256 | 0.18 (0.17, 0.19) | 0.19 (0.18, 0.20) | 0.18 (0.17, 0.19) |
|  |  |  |  |  |
| High NSES | 109,102 | Ref. | Ref. | Ref. |
| Moderate NSES | 109,422 | 0.02 (0.01, 0.02) | 0.02 (0.01, 0.02) | 0.01 (0.00, 0.01) |
| Low NSES | 109,117 | 0.06 (0.05, 0.06) | 0.06 (0.05, 0.07) | 0.03 (0.02, 0.04) |
| **Processing speed (N=325,838)** | | | | |
| High ISES | 70,484 | Ref. | Ref. | Ref. |
| Moderate ISES | 176,173 | 0.10 (0.10, 0.11) | 0.10 (0.09, 0.10) | 0.09 (0.08, 0.10) |
| Low ISES | 79,181 | 0.28 (0.27, 0.29) | 0.26 (0.25, 0.27) | 0.24 (0.23, 0.25) |
|  |  |  |  |  |
| High NSES | 108,785 | Ref. | Ref. | Ref. |
| Moderate NSES | 108,917 | 0.04 (0.03, 0.05) | 0.04 (0.03, 0.05) | 0.03 (0.02, 0.03) |
| Low ISES | 108,136 | 0.13 (0.12, 0.14) | 0.11 (0.11, 0.12) | 0.08 (0.07, 0.09) |
| **Verbal/numeric reasoning (N=108,227)** | | | | |
| High ISES | 24,106 | Ref. | Ref. | Ref. |
| Moderate ISES | 58,689 | -0.47 (-0.48, -0.45) | -0.45 (-0.46, -0.43) | -0.44 (-0.46, -0.43) |
| Low ISES | 25,432 | -0.92 (-0.94, -0.91) | -0.88 (-0.89, -0.86) | -0.87 (-0.89, -0.85) |
|  |  |  |  |  |
| High NSES | 31,398 | Ref. | Ref. | Ref. |
| Moderate NSES | 37,550 | -0.06 (-0.07, -0.04) | -0.05 (-0.06, -0.03) | -0.02 (-0.03, 0.00) |
| Low NSES | 39,279 | -0.15 (-0.16, -0.13) | -0.12 (-0.14, -0.11) | -0.04 (-0.05, -0.02) |
| **Prospective memory (N=110,305)** | | | | |
| High ISES | 3,004/24,230 | Ref. | Ref. | Ref. |
| Moderate ISES | 10,760/59,452 | 1.43 (1.37, 1.50) | 1.41 (1.35, 1.48) | 1.40 (1.34, 1.47) |
| Low NSES | 8,505/26,623 | 2.78 (2.65, 2.92) | 2.67 (2.54, 2.81) | 2.59 (2.46, 2.72) |
|  |  |  |  |  |
| High NSES | 5,368/31,752 | Ref. | Ref. | Ref. |
| Moderate NSES | 7,251/38,081 | 1.13 (1.09, 1.18) | 1.12 (1.08, 1.17) | 1.08 (1.04, 1.12) |
| Low NSES | 9,650/40,472 | 1.38 (1.32, 1.43) | 1.34 (1.28, 1.39) | 1.19 (1.15, 1.24) |

Abbreviations: SES, socioeconomic status; OR, odds ratio; CI, confidence interval; ISES, individual socioeconomic status; NSES, neighborhood socioeconomic status.

^*^ Model 1: adjusted for age, gender, ethnicity, family history of Alzheimer's disease, and apolipoprotein E genotypes.

^†^ Model 2: further adjusted for smoking status, alcohol consumption, healthy diet, regular exercise, sleep duration, body mass index, waist circumference, systolic blood pressure, and diastolic blood pressure based on model 1.

^‡^ Model 3: ISES and NSES were added into the model simultaneously based on model 2.

# Table S29. Associations of ISES with cognitive function stratified by NSES.^*^

|  | | **No. of participant** | **β/OR (95% CI)** |
| --- | --- | --- | --- |
| **Visual memory (N=327,641)** | | | |
| High NSES | High ISES | 28,758 | Ref. |
|  | Moderate ISES | 62,673 | 0.09 (0.07, 0.10) |
|  | Low ISES | 17,671 | 0.16 (0.14, 0.18) |
| Moderate NSES | High ISES | 23,152 | Ref. |
|  | Moderate ISES | 61,680 | 0.08 (0.06, 0.09) |
|  | Low ISES | 24,590 | 0.17 (0.16, 0.19) |
| Low NSES | High ISES | 18,680 | Ref. |
|  | Moderate ISES | 52,442 | 0.08 (0.07, 0.10) |
|  | Low ISES | 37,995 | 0.20 (0.18, 0.22) |
|  |  |  |  |
| **Processing speed (N=325,838)** | | | |
| High NSES | High ISES | 28,732 | Ref. |
|  | Moderate ISES | 62,520 | 0.08 (0.07, 0.10) |
|  | Low ISES | 17,533 | 0.20 (0.18, 0.22) |
| Moderate NSES | High ISES | 23,111 | Ref. |
|  | Moderate ISES | 61,482 | 0.09 (0.07, 0.10) |
|  | Low ISES | 24,324 | 0.21 (0.19, 0.22) |
| Low NSES | High ISES | 18,641 | Ref. |
|  | Moderate ISES | 52,171 | 0.10 (0.09, 0.12) |
|  | Low ISES | 37,324 | 0.29 (0.27, 0.31) |
|  |  |  |  |
| **Verbal/numeric reasoning (N=108,227)** | | | |
| High NSES | High ISES | 7,936 | Ref. |
|  | Moderate ISES | 18,276 | -0.41 (-0.43, -0.38) |
|  | Low ISES | 5,186 | -0.79 (-0.82, -0.75) |
| Moderate NSES | High ISES | 8,366 | Ref. |
|  | Moderate ISES | 21,203 | -0.45 (-0.47, -0.42) |
|  | Low ISES | 7,981 | -0.87 (-0.90, -0.84) |
| Low NSES | High ISES | 7,804 | Ref. |
|  | Moderate ISES | 19,210 | -0.48 (-0.50, -0.45) |
|  | Low ISES | 12,265 | -0.92 (-0.94, -0.89) |
|  |  |  |  |
| **Prospective memory (N=110,305)** | | | |
| High NSES | High ISES | 887/7,967 | Ref. |
|  | Moderate ISES | 3,026/18,439 | 1.38 (1.27, 1.50) |
|  | Low ISES | 1,455/5,346 | 2.29 (2.07, 2.53) |
| Moderate NSES | High ISES | 1,045/8,410 | Ref. |
|  | Moderate ISES | 3,712/21,416 | 1.40 (1.29, 1.51) |
|  | Low ISES | 2,494/8,255 | 2.57 (2.36, 2.81) |
| Low NSES | High ISES | 1,072/7,853 | Ref. |
|  | Moderate ISES | 4,022/19,597 | 1.41 (1.31, 1.52) |
|  | Low ISES | 4,556/13,022 | 2.75 (2.54, 2.98) |

Abbreviations: ISES, individual socioeconomic status; NSES, neighborhood socioeconomic status; OR, odds ratio; CI, confidence interval.

**^*^** The models were adjusted for age, gender, ethnicity, family history of Alzheimer's disease, apolipoprotein E genotypes, smoking status, alcohol consumption, healthy diet, regular exercise, sleep duration, body mass index, waist circumference, systolic blood pressure, and diastolic blood pressure.

# Table S30. Joint associations of ISES and NSES with cognitive function.^*^

| **SES** | **No. of participant** | **β/OR (95% CI)** |
| --- | --- | --- |
| **Visual memory (N=327,641)** |  |  |
| High ISES & High NSES | 28,758 | Ref. |
| Moderate ISES & High NSES | 62,673 | 0.09 (0.08, 0.10) |
| Low ISES & High NSES | 17,671 | 0.17 (0.15, 0.19) |
| High ISES & Moderate NSES | 23,152 | 0.01 (-0.01, 0.03) |
| Moderate ISES & Moderate NSES | 61,680 | 0.09 (0.08, 0.10) |
| Low ISES & Moderate NSES | 24,590 | 0.19 (0.17, 0.20) |
| High ISES & Low NSES | 18,680 | 0.03 (0.01, 0.05) |
| Moderate ISES & Low NSES | 52,442 | 0.11 (0.10, 0.13) |
| Low ISES & Low NSES | 37,995 | 0.22 (0.21, 0.24) |
| **Processing speed (N=325,838)** |  |  |
| High ISES & High NSES | 28,732 | Ref. |
| Moderate ISES & High NSES | 62,520 | 0.09 (0.07, 0.10) |
| Low ISES & High NSES | 17,533 | 0.21 (0.19, 0.22) |
| High ISES & Moderate NSES | 23,111 | 0.03 (0.01, 0.04) |
| Moderate ISES & Moderate NSES | 61,482 | 0.12 (0.10, 0.13) |
| Low ISES & Moderate NSES | 24,324 | 0.24 (0.22, 0.25) |
| High ISES & Low NSES | 18,641 | 0.05 (0.03, 0.07) |
| Moderate ISES & Low NSES | 52,171 | 0.15 (0.14, 0.17) |
| Low ISES & Low NSES | 37,324 | 0.34 (0.33, 0.36) |
| **Verbal/numeric reasoning (N=108,227)** |  |  |
| High ISES & High NSES | 7,936 | Ref. |
| Moderate ISES & High NSES | 18,276 | -0.41 (-0.44, -0.39) |
| Low ISES & High NSES | 5,186 | -0.80 (-0.83, -0.76) |
| High ISES & Moderate NSES | 8,366 | 0.01 (-0.01, 0.04) |
| Moderate ISES & Moderate NSES | 21,203 | -0.43 (-0.45, -0.41) |
| Low ISES & Moderate NSES | 7,981 | -0.86 (-0.88, -0.83) |
| High ISES & Low NSES | 7,804 | 0.02 (0.00, 0.05) |
| Moderate ISES & Low NSES | 19,210 | -0.45 (-0.48, -0.43) |
| Low ISES & Low NSES | 12,265 | -0.90 (-0.92, -0.87) |
| **Prospective memory (N=110,305)** |  |  |
| High ISES & High NSES | 887/7,967 | Ref. |
| Moderate ISES & High NSES | 3,026/18,439 | 1.44 (1.33, 1.56) |
| Low ISES & High NSES | 1,455/5,346 | 2.47 (2.24, 2.71) |
| High ISES & Moderate NSES | 1,045/8,410 | 1.10 (1.00, 1.21) |
| Moderate ISES & Moderate NSES | 3,712/21,416 | 1.52 (1.40, 1.64) |
| Low ISES & Moderate NSES | 2,494/8,255 | 2.77 (2.54, 3.02) |
| High ISES & Low NSES | 1,072/7,853 | 1.17 (1.06, 1.29) |
| Moderate ISES & Low NSES | 4,022/19,597 | 1.64 (1.52, 1.78) |
| Low ISES & Low NSES | 4,556/13,022 | 3.19 (2.94, 3.47) |

Abbreviations: ISES, individual socioeconomic status; NSES, neighborhood socioeconomic status; OR, odds ratio; CI, confidence interval.

^*^ The models were adjusted for age, gender, ethnicity, family history of Alzheimer's disease, apolipoprotein E genotypes, smoking status, alcohol consumption, healthy diet, regular exercise, sleep duration, body mass index, waist circumference, systolic blood pressure, and diastolic blood pressure.

# Table S31. Associations of SES profiles with cognitive function.^*^

|  | **No. of participant** | **β/OR (95% CI)** |
| --- | --- | --- |
| **Visual memory (N=327,641)** |  |  |
| Balanced-high SES subpopulation | 155,592 | Ref. |
| Low income-less educated subpopulation | 91,030 | 0.15 (0.14, 0.15) |
| Low income-NSES subpopulation | 61,317 | 0.09 (0.08, 0.10) |
| Low SES subpopulation | 19,702 | 0.12 (0.10, 0.13) |
| **Processing speed (N=325,838)** |  |  |
| Balanced-high SES subpopulation | 155,280 | Ref. |
| Low income-less educated subpopulation | 90,300 | 0.13 (0.12, 0.14) |
| Low income-NSES subpopulation | 60,801 | 0.13 (0.12, 0.14) |
| Low SES subpopulation | 1,9457 | 0.21 (0.20, 0.23) |
| **Verbal/numeric reasoning (N=108,227)** |  |  |
| Balanced-high SES subpopulation | 51,311 | Ref. |
| Low income-less educated subpopulation | 28,379 | -0.69 (-0.70, -0.67) |
| Low income-NSES subpopulation | 21,568 | -0.31 (-0.33, -0.30) |
| Low SES subpopulation | 6,969 | -0.48 (-0.50, -0.46) |
| **Prospective memory (N=110,305)** |  |  |
| Balanced-high SES subpopulation | 7,132/51,618 | Ref. |
| Low income-less educated subpopulation | 7,959/29,247 | 2.01 (1.94, 2.09) |
| Low income-NSES subpopulation | 5,240/22,202 | 1.55 (1.48, 1.61) |
| Low SES subpopulation | 1,938/7,238 | 2.14 (2.01, 2.27) |

Abbreviations: SES, socioeconomic status; OR, odds ratio; CI, confidence interval; NSES, neighborhood socioeconomic status.

**^*^** The models were adjusted for age, gender, ethnicity, family history of Alzheimer's disease, apolipoprotein E genotypes, smoking status, alcohol consumption, healthy diet, regular exercise, sleep duration, body mass index, waist circumference, systolic blood pressure, and diastolic blood pressure.

# Table S32. Associations of SES with the volume of WMH (N=34,591).

|  | **No. of participant** | **Model 1^*^** | **Model 2^†^** | **Model 3^‡^** |
| --- | --- | --- | --- | --- |
|  |  | **β (95% CI)** | **β (95% CI)** | **β (95% CI)** |
| High ISES | 10,641 | Ref. | Ref. | Ref. |
| Moderate ISES | 19,247 | 0.04 (0.02, 0.06) | 0.02 (0.00, 0.04) | 0.02 (0.00, 0.04) |
| Low ISES | 4,703 | 0.12 (0.09, 0.15) | 0.09 (0.06, 0.12) | 0.09 (0.05, 0.12) |
|  |  |  |  |  |
| High NSES | 13,119 | Ref. | Ref. | Ref. |
| Moderate NSES | 11,778 | 0.00 (-0.03, 0.02) | -0.01 (-0.03, 0.01) | -0.01 (-0.04, 0.01) |
| Low NSES | 9,694 | 0.03 (0.01, 0.05) | 0.01 (-0.01, 0.03) | 0.00 (-0.02, 0.03) |

Abbreviations: SES, socioeconomic status; WMH, white matter hyperintensity; CI, confidence interval; ISES, individual socioeconomic status; NSES, neighborhood socioeconomic status.

^*^ Model 1: adjusted for age, gender, ethnicity, family history of Alzheimer's disease, and apolipoprotein E genotypes.

^†^ Model 2: further adjusted for smoking status, alcohol consumption, healthy diet, regular exercise, sleep duration, body mass index, waist circumference, systolic blood pressure, and diastolic blood pressure based on model 1.

^‡^ Model 3: ISES and NSES were added into the model simultaneously based on model 2.

# Table S33. Associations of ISES with the volume of WMH stratified by NSES (N=34,591).^*^

|  | | **No. of participant** | **β (95% CI)** |
| --- | --- | --- | --- |
| High NSES | High ISES | 4,642 | Ref. |
|  | Moderate ISES | 7,252 | 0.01 (-0.03, 0.04) |
|  | Low ISES | 1,225 | 0.09 (0.03, 0.15) |
| Moderate NSES | High ISES | 3,496 | Ref. |
|  | Moderate ISES | 6,697 | 0.04 (0.00, 0.07) |
|  | Low ISES | 1,585 | 0.10 (0.05, 0.15) |
| Low NSES | High ISES | 2,503 | Ref. |
|  | Moderate ISES | 5,298 | 0.02 (-0.02, 0.06) |
|  | Low ISES | 1,893 | 0.07 (0.01, 0.12) |

Abbreviations: ISES, individual socioeconomic status; WMH, white matter hyperintensity; NSES, neighborhood socioeconomic status; CI, confidence interval.

^*^ The models were adjusted for age, gender, ethnicity, family history of Alzheimer's disease, apolipoprotein E genotypes, smoking status, alcohol consumption, healthy diet, regular exercise, sleep duration, body mass index, waist circumference, systolic blood pressure, and diastolic blood pressure.

# Table S34. Joint associations of ISES and NSES with the volume of WMH (N=34,591).^*^

|  | **No. of participant** | **β (95% CI)** |
| --- | --- | --- |
|  |  |  |
| High ISES & High NSES | 4,642 | Ref. |
| Moderate ISES & High NSES | 7,252 | 0.01 (-0.02, 0.04) |
| Low ISES & High NSES | 1,225 | 0.09 (0.04, 0.15) |
| High ISES & Moderate NSES | 3,496 | -0.03 (-0.07, 0.01) |
| Moderate ISES & Moderate NSES | 6,697 | 0.00 (-0.03, 0.04) |
| Low ISES & Moderate NSES | 1,585 | 0.07 (0.02, 0.12) |
| High ISES & Low NSES | 2,503 | 0.00 (-0.04, 0.04) |
| Moderate ISES & Low NSES | 5,298 | 0.02 (-0.01, 0.05) |
| Low ISES & Low NSES | 1,893 | 0.07 (0.02, 0.12) |

Abbreviations: ISES, individual socioeconomic status; NSES, neighborhood socioeconomic status; WMH, white matter hyperintensity; CI, confidence interval.

^*^ The models were adjusted for age, gender, ethnicity, family history of Alzheimer's disease, apolipoprotein E genotypes, smoking status, alcohol consumption, healthy diet, regular exercise, sleep duration, body mass index, waist circumference, systolic blood pressure, and diastolic blood pressure.

# Table S35. Associations of SES profiles with the volume of WMH (N=34,591).^*^

| **SES** | **No. of participant** | **β (95% CI)** |
| --- | --- | --- |
|  |  |  |
| Balanced-high SES subpopulation | 21,108 | Ref. |
| Low income-less educated subpopulation | 6,624 | 0.05 (0.03, 0.07) |
| Low income-NSES subpopulation | 5,398 | 0.04 (0.02, 0.07) |
| Low SES subpopulation | 1,461 | 0.02 (-0.02, 0.07) |

Abbreviations: SES, socioeconomic status; WMH, white matter hyperintensity; CI, confidence interval; NSES, neighborhood socioeconomic status.

^*^ The models were adjusted for age, gender, ethnicity, family history of Alzheimer's disease, apolipoprotein E genotypes, smoking status, alcohol consumption, healthy diet, regular exercise, sleep duration, body mass index, waist circumference, systolic blood pressure, and diastolic blood pressure.**Table S36. Associations of SES with cognitive function change.**

| **SES** | **No. of participant** | **Model 1**^*^ | **Model 2^†^** |
| --- | --- | --- | --- |
|  |  | **β/OR (95% CI)** | **β/OR (95% CI)** |
| **Visual memory (N=63,607)** |  |  |  |
| High ISES × Time | 18,992 | Ref. | Ref. |
| Moderate ISES × Time | 35,686 | 0.003 (0.001, 0.004) | 0.003 (0.001, 0.004) |
| Low ISES × Time | 8,929 | 0.003 (0.000, 0.006)) | 0.003 (0.000, 0.006) |
|  |  |  |  |
| High NSES × Time | 24,376 | Ref. | Ref. |
| Moderate NSES × Time | 21,575 | -0.001 (-0.003, 0.001) | -0.001 (-0.003, 0.001) |
| Low NSES × Time | 17,656 | -0.001 (-0.003, 0.002) | 0.000 (-0.003, 0.002) |
| **Processing speed (N=62,345)** |  |  |  |
| High ISES × Time | 18,664 | Ref. | Ref. |
| Moderate ISES × Time | 34,971 | 0.002 (0.001, 0.004) | 0.002 (0.001, 0.004) |
| Low ISES × Time | 8,710 | 0.003 (0.001, 0.005) | 0.003 (0.001, 0.005) |
|  |  |  |  |
| High NSES × Time | 23,907 | Ref. | Ref. |
| Moderate NSES × Time | 21,132 | -0.001 (-0.002, 0.001) | -0.001 (-0.002, 0.001) |
| Low ISES × Time | 17,306 | -0.001 (-0.003, 0.000) | -0.001 (-0.003, 0.000) |
| **Verbal/numeric reasoning (N=61,893)** |  |  |  |
| High ISES × Time | 18,634 | Ref. | Ref. |
| Moderate ISES × Time | 34,713 | -0.005 (-0.007, -0.003) | -0.005 (-0.007, -0.003) |
| Low ISES × Time | 8,546 | -0.009 (-0.012, -0.006) | -0.009 (-0.012, -0.006) |
|  |  |  |  |
| High NSES × Time | 23,763 | Ref. | Ref. |
| Moderate NSES × Time | 20,979 | -0.001 (-0.003, 0.002) | 0.000 (-0.002, 0.002) |
| Low NSES × Time | 17,151 | 0.000 (-0.002, 0.002) | 0.000 (-0.002, 0.003) |
| **Prospective memory (N=17,039)** |  |  |  |
| High ISES | 438/5,434 | Ref. | Ref. |
| Moderate ISES | 1,144/9,529 | 1.38 (1.23, 1.56) | 1.38 (1.22, 1.55) |
| Low NSES | 351/2,076 | 1.87 (1.60, 2.20) | 1.82 (1.55, 2.14) |
|  |  |  |  |
| High NSES | 634/6,047 | Ref. | Ref. |
| Moderate NSES | 652/6,021 | 1.04 (0.93, 1.17) | 1.02 (0.91, 1.15) |
| Low NSES | 647/4,971 | 1.27 (1.13, 1.43) | 1.22 (1.08, 1.38) |

Abbreviations: SES, socioeconomic status; OR, odds ratio; CI, confidence interval; ISES, individual socioeconomic status; NSES, neighborhood socioeconomic status.

^*^ Model 1: adjusted for age, gender, ethnicity, family history of Alzheimer's disease, apolipoprotein E genotypes, smoking status, alcohol consumption, healthy diet, regular exercise, sleep duration, body mass index, waist circumference, systolic blood pressure, and diastolic blood pressure.

^†^ Model 2: ISES and NSES were added into the model simultaneously based on model 1.

# Table S37. Associations of ISES with cognitive function change stratified by NSES.^*^

|  | | **No. of participant** | **β/OR (95% CI)** |
| --- | --- | --- | --- |
| **Visual memory (N=63,607)** | | | |
| High NSES | High ISES × Time | 8,331 | Ref. |
|  | Moderate ISES × Time | 13,668 | 0.002 (-0.001, 0.005) |
|  | Low ISES × Time | 2,377 | 0.007 (0.001, 0.012) |
| Moderate NSES | High ISES × Time | 6,150 | Ref. |
|  | Moderate ISES × Time | 12,448 | 0.003 (-0.001, 0.006) |
|  | Low ISES × Time | 2,977 | 0.001 (-0.004, 0.006) |
| Low NSES | High ISES × Time | 4,511 | Ref. |
|  | Moderate ISES × Time | 9,570 | 0.004 (0.000, 0.007) |
|  | Low ISES × Time | 3,575 | 0.003 (-0.002, 0.008) |
|  |  |  |  |
| **Processing speed (N=62,345)** | | | |
| High NSES | High ISES × Time | 8,194 | Ref. |
|  | Moderate ISES × Time | 13,393 | 0.004 (0.001, 0.006) |
|  | Low ISES × Time | 2,320 | 0.007 (0.003, 0.011) |
| Moderate NSES | High ISES × Time | 6,053 | Ref. |
|  | Moderate ISES × Time | 12,185 | 0.001 (-0.001, 0.004) |
|  | Low ISES × Time | 2,894 | 0.004 (0.000, 0.008) |
| Low NSES | High ISES × Time | 4,417 | Ref. |
|  | Moderate ISES × Time | 9,393 | 0.001 (-0.002, 0.004) |
|  | Low ISES × Time | 3,496 | -0.001 (-0.005, 0.003) |
|  |  |  |  |
| **Verbal/numeric reasoning (N=61,893)** | | | |
| High NSES | High ISES × Time | 8,186 | Ref. |
|  | Moderate ISES × Time | 13,300 | -0.006 (-0.009, -0.003) |
|  | Low ISES × Time | 2,277 | -0.013 (-0.019, -0.007) |
| Moderate NSES | High ISES × Time | 6,036 | Ref. |
|  | Moderate ISES × Time | 12,099 | -0.005 (-0.008, -0.001) |
|  | Low ISES × Time | 2,844 | -0.008 (-0.014, -0.003) |
| Low NSES | High ISES × Time | 4,412 | Ref. |
|  | Moderate ISES × Time | 9,314 | -0.004 (-0.008, 0.000) |
|  | Low ISES × Time | 3,425 | -0.007 (-0.013, -0.002) |
|  |  |  |  |
| **Prospective memory (N=17,039)** | | | |
| High NSES | High ISES | 168/2,077 | Ref. |
|  | Moderate ISES | 385/3,450 | 1.21 (0.99, 1.48) |
|  | Low ISES | 81/520 | 1.64 (1.21, 2.21) |
| Moderate NSES | High ISES | 147/1,867 | Ref. |
|  | Moderate ISES | 394/3,438 | 1.34 (1.09, 1.64) |
|  | Low ISES | 111/716 | 1.69 (1.28, 2.24) |
| Low NSES | High ISES | 123/1,490 | Ref. |
|  | Moderate ISES | 365/2,641 | 1.65 (1.32, 2.05) |
|  | Low ISES | 159/840 | 2.17 (1.66, 2.83) |

Abbreviations: ISES, individual socioeconomic status; NSES, neighborhood socioeconomic status; OR, odds ratio; CI, confidence interval.

**^*^** The models were adjusted for age, gender, ethnicity, family history of Alzheimer's disease, apolipoprotein E genotypes, smoking status, alcohol consumption, healthy diet, regular exercise, sleep duration, body mass index, waist circumference, systolic blood pressure, and diastolic blood pressure.

# Table S38. Joint associations of ISES and NSES with cognitive function change.^*^

| **SES** | **No. of participant** | **β/OR (95% CI)** |
| --- | --- | --- |
| **Visual memory (N=63,607)** |  |  |
| High ISES & High NSES × Time | 8,331 | Ref. |
| Moderate ISES & High NSES × Time | 13,668 | 0.002 (-0.001, 0.005) |
| Low ISES & High NSES × Time | 2,377 | 0.007 (0.001, 0.012) |
| High ISES & Moderate NSES × Time | 6,150 | -0.001 (-0.004, 0.003) |
| Moderate ISES & Moderate NSES × Time | 12,448 | 0.002 (-0.001, 0.005) |
| Low ISES & Moderate NSES × Time | 2,977 | 0.000 (-0.004, 0.005) |
| High ISES & Low NSES × Time | 4,511 | -0.001 (-0.005, 0.002) |
| Moderate ISES & Low NSES × Time | 9,570 | 0.002 (-0.001, 0.005) |
| Low ISES & Low NSES × Time | 3,575 | 0.002 (-0.002, 0.006) |
| **Processing speed (N=62,345)** |  |  |
| High ISES & High NSES × Time | 8,194 | Ref. |
| Moderate ISES & High NSES × Time | 13,393 | 0.004 (0.001, 0.006) |
| Low ISES & High NSES × Time | 2,320 | 0.007 (0.003, 0.011) |
| High ISES & Moderate NSES × Time | 6,053 | 0.001 (-0.002, 0.003) |
| Moderate ISES & Moderate NSES × Time | 12,185 | 0.002 (0.000, 0.004) |
| Low ISES & Moderate NSES × Time | 2,894 | 0.005 (0.001, 0.008) |
| High ISES & Low NSES × Time | 4,417 | 0.001 (-0.002, 0.004) |
| Moderate ISES & Low NSES × Time | 9,393 | 0.002 (0.000, 0.004) |
| Low ISES & Low NSES × Time | 3,496 | 0.000 (-0.003, 0.003) |
| **Verbal/numeric reasoning (N=61,893)** |  |  |
| High ISES & High NSES × Time | 8,186 | Ref. |
| Moderate ISES & High NSES × Time | 13,300 | -0.006 (-0.009, -0.003) |
| Low ISES & High NSES × Time | 2,277 | -0.013 (-0.019, -0.007) |
| High ISES & Moderate NSES × Time | 6,036 | -0.001 (-0.005, 0.003) |
| Moderate ISES & Moderate NSES × Time | 12,099 | -0.006 (-0.009, -0.002) |
| Low ISES & Moderate NSES × Time | 2,844 | -0.009 (-0.015, -0.004) |
| High ISES & Low NSES × Time | 4,412 | -0.001 (-0.005, 0.003) |
| Moderate ISES & Low NSES × Time | 9,314 | -0.005 (-0.008, -0.001) |
| Low ISES & Low NSES × Time | 3,425 | -0.009 (-0.013, -0.004) |
| **Prospective memory (N=17,039)** |  |  |
| High ISES & High NSES | 168/2,077 | Ref. |
| Moderate ISES & High NSES | 385/3,450 | 1.21 (1.00, 1.47) |
| Low ISES & High NSES | 81/520 | 1.60 (1.19, 2.14) |
| High ISES & Moderate NSES | 147/1,867 | 0.95 (0.76, 1.20) |
| Moderate ISES & Moderate NSES | 394/3,438 | 1.27 (1.05, 1.55) |
| Low ISES & Moderate NSES | 111/716 | 1.64 (1.26, 2.14) |
| High ISES & Low NSES | 123/1,490 | 0.96 (0.75, 1.23) |
| Moderate ISES & Low NSES | 365/2,641 | 1.59 (1.31, 1.94) |
| Low ISES & Low NSES | 159/840 | 2.11 (1.66, 2.68) |

Abbreviations: ISES, individual socioeconomic status; NSES, neighborhood socioeconomic status; OR, odds ratio; CI, confidence interval.

^*^ The models were adjusted for age, gender, ethnicity, family history of Alzheimer's disease, apolipoprotein E genotypes, smoking status, alcohol consumption, healthy diet, regular exercise, sleep duration, body mass index, waist circumference, systolic blood pressure, and diastolic blood pressure.

# Table S39. Associations of SES profiles with cognitive function change.^*^

|  | **No. of participant** | **β/OR (95% CI)** |
| --- | --- | --- |
| **Visual memory (N=63,607)** |  |  |
| Balanced-high SES subpopulation × Time | 38,377 | Ref. |
| Low income-less educated subpopulation × Time | 12,592 | 0.003 (0.000, 0.005) |
| Low income-NSES subpopulation × Time | 9,919 | 0.001 (-0.002, 0.003) |
| Low SES subpopulation × Time | 2,719 | -0.001 (-0.005, 0.003) |
| **Processing speed (N=62,345)** |  |  |
| Balanced-high SES subpopulation × Time | 37,657 | Ref. |
| Low income-less educated subpopulation × Time | 12,293 | 0.004 (0.002, 0.006) |
| Low income-NSES subpopulation × Time | 9,739 | 0.000 (-0.001, 0.002) |
| Low SES subpopulation × Time | 2,656 | -0.001 (-0.004, 0.002) |
| **Verbal/numeric reasoning (N=61,893)** |  |  |
| Balanced-high SES subpopulation × Time | 37,506 | Ref. |
| Low income-less educated subpopulation × Time | 12,105 | -0.006 (-0.008, -0.003) |
| Low income-NSES subpopulation × Time | 9,660 | 0.000 (-0.003, 0.002) |
| Low SES subpopulation × Time | 2,622 | -0.002 (-0.007, 0.002) |
| **Prospective memory (N=17,039)** |  |  |
| Balanced-high SES subpopulation | 969/10,663 | Ref. |
| Low income-less educated subpopulation | 484/2,881 | 1.71 (1.51, 1.93) |
| Low income-NSES subpopulation | 367/2,683 | 1.49 (1.31, 1.71) |
| Low SES subpopulation | 113/812 | 1.72 (1.39, 2.13) |

Abbreviations: SES, socioeconomic status; OR, odds ratio; CI, confidence interval; NSES, neighborhood socioeconomic status.

**^*^** The models were adjusted for age, gender, ethnicity, family history of Alzheimer's disease, apolipoprotein E genotypes, smoking status, alcohol consumption, healthy diet, regular exercise, sleep duration, body mass index, waist circumference, systolic blood pressure, and diastolic blood pressure.

# Table S40. Associations of SES with the volume of WMH change (N=3,709).

|  | **No. of participant** | **Model 1^*^** | **Model 2^†^** |
| --- | --- | --- | --- |
|  |  | **β (95% CI)** | **β (95% CI)** |
| High ISES | 1,210 | Ref. | Ref. |
| Moderate ISES | 3,074 | -0.013 (-0.053, 0.027) | -0.016 (-0.056, 0.024) |
| Low ISES | 425 | 0.005 (-0.058, 0.068) | -0.001 (-0.064, 0.063) |
|  |  |  |  |
| High NSES | 1,412 | Ref. | Ref. |
| Moderate NSES | 1,272 | 0.022 (-0.020, 0.064) | 0.023 (-0.019, 0.064) |
| Low NSES | 1,025 | 0.025 (-0.020, 0.071) | 0.026 (-0.019, 0.072) |

Abbreviations: SES, socioeconomic status; WMH, white matter hyperintensity; CI, confidence interval; ISES, individual socioeconomic status; NSES, neighborhood socioeconomic status.

^*^ Model 1: adjusted for age, gender, ethnicity, family history of Alzheimer's disease, apolipoprotein E genotypes, smoking status, alcohol consumption, healthy diet, regular exercise, sleep duration, body mass index, waist circumference, systolic blood pressure, diastolic blood pressure, the volume of WMH at baseline, and the period between two assessments.

^†^ Model 2: ISES and NSES were added into the model simultaneously based on model 1.

# Table S41. Associations of ISES with the volume of WMH change stratified by NSES (N=3,709).^*^

|  | | **No. of participant** | **β (95% CI)** |
| --- | --- | --- | --- |
| High NSES | High ISES | 553 | Ref. |
|  | Moderate ISES | 748 | -0.022 (-0.086, 0.042) |
|  | Low ISES | 111 | -0.018 (-0.138, 0.103) |
| Moderate NSES | High ISES | 401 | Ref. |
|  | Moderate ISES | 730 | -0.017 (-0.086, 0.052) |
|  | Low ISES | 141 | 0.018 (-0.093, 0.129) |
| Low NSES | High ISES | 256 | Ref. |
|  | Moderate ISES | 596 | -0.002 (-0.083, 0.078) |
|  | Low ISES | 173 | -0.009 (-0.118, 0.100) |

Abbreviations: ISES, individual socioeconomic status; WMH, white matter hyperintensity; NSES, neighborhood socioeconomic status; CI, confidence interval.

^*^ The models were adjusted for age, gender, ethnicity, family history of Alzheimer's disease, apolipoprotein E genotypes, smoking status, alcohol consumption, healthy diet, regular exercise, sleep duration, body mass index, waist circumference, systolic blood pressure, diastolic blood pressure, the volume of WMH at baseline, and the period between two assessments.

# Table S42. Joint associations of ISES and NSES with the volume of WMH change (N=3,709).^*^

|  | **No. of participant** | **β (95% CI)** |
| --- | --- | --- |
|  |  |  |
| High ISES & High NSES | 553 | Ref. |
| Moderate ISES & High NSES | 748 | -0.022 (-0.084, 0.039) |
| Low ISES & High NSES | 111 | -0.016 (-0.013, 0.098) |
| High ISES & Moderate NSES | 401 | 0.017 (-0.054, 0.088) |
| Moderate ISES & Moderate NSES | 730 | -0.001 (-0.062, 0.061) |
| Low ISES & Moderate NSES | 141 | 0.036 (-0.068, 0.140) |
| High ISES & Low NSES | 256 | 0.013 (-0.069, 0.095) |
| Moderate ISES & Low NSES | 596 | 0.012 (-0.052, 0.076) |
| Low ISES & Low NSES | 173 | 0.013 (-0.083, 0.108) |

Abbreviations: ISES, individual socioeconomic status; NSES, neighborhood socioeconomic status; WMH, white matter hyperintensity; CI, confidence interval.

^*^ The models were adjusted for age, gender, ethnicity, family history of Alzheimer's disease, apolipoprotein E genotypes, smoking status, alcohol consumption, healthy diet, regular exercise, sleep duration, body mass index, waist circumference, systolic blood pressure, diastolic blood pressure, the volume of WMH at baseline, and the period between two assessments.

# Table S43. Associations of SES profiles with the volume of WMH change (N=3,709).^*^

| **SES profiles** | **No. of participant** | **β (95% CI)** |
| --- | --- | --- |
|  |  |  |
| Balanced-high SES subpopulation | 2,379 | Ref. |
| Low income-less educated subpopulation | 613 | -0.010 (-0.060, 0.040) |
| Low income-NSES subpopulation | 563 | -0.016 (-0.067, 0.035) |
| Low SES subpopulation | 154 | -0.018 (-0.108, 0.072) |

Abbreviations: SES, socioeconomic status; WMH, white matter hyperintensity; CI, confidence interval; NSES, neighborhood socioeconomic status.

^*^ The models were adjusted for age, gender, ethnicity, family history of Alzheimer's disease, apolipoprotein E genotypes, smoking status, alcohol consumption, healthy diet, regular exercise, sleep duration, body mass index, waist circumference, systolic blood pressure, diastolic blood pressure, the volume of WMH at baseline, and the period between two assessments.

# Table S44. Associations of SES profiles with blood inflammatory markers (N=303,981).^*^

|  | **Balanced-high SES subpopulation** | | **Low income-less educated subpopulation** | | **Low income-NSES subpopulation** | | **Low SES subpopulation** | |
| --- | --- | --- | --- | --- | --- | --- | --- | --- |
|  | **β (95% CI)** | **FDR** | **β (95% CI)** | **FDR** | **β (95% CI)** | **FDR** | **β (95% CI)** | **FDR** |
| Leukocyte count | Ref. | — | 0.06 (0.06, 0.07) | <0.001 | 0.04 (0.03, 0.05) | <0.001 | 0.09 (0.08, 0.10) | <0.001 |
| Neutrophil count | Ref. | — | 0.08 (0.07, 0.09) | <0.001 | 0.06 (0.05, 0.07) | <0.001 | 0.13 (0.11, 0.14) | <0.001 |
| Neutrophil percentage | Ref. | — | 0.07 (0.06, 0.08) | <0.001 | 0.06 (0.05, 0.07) | <0.001 | 0.13 (0.11, 0.14) | <0.001 |
| Monocyte count | Ref. | — | 0.03 (0.02, 0.04) | <0.001 | 0.02 (0.01, 0.02) | 0.002 | -0.01 (-0.03, 0.00) | 0.073 |
| Monocyte percentage | Ref. | — | -0.02 (-0.03, -0.01) | <0.001 | -0.01 (-0.02, -0.01) | 0.002 | -0.08 (-0.09, -0.06) | <0.001 |
| Lymphocyte count | Ref. | — | -0.02 (-0.02, -0.01) | <0.001 | -0.03 (-0.04, -0.02) | <0.001 | -0.07 (-0.08, -0.05) | <0.001 |
| Lymphocyte percentage | Ref. | — | -0.08 (-0.09, -0.07) | <0.001 | -0.08 (-0.09, -0.07) | <0.001 | -0.16 (-0.18, -0.15) | <0.001 |
| C-reactive protein | Ref. | — | 0.08 (0.08, 0.09) | <0.001 | 0.05 (0.04, 0.06) | <0.001 | 0.16 (0.15, 0.18) | <0.001 |
| Platelet count | Ref. | — | 0.04 (0.03, 0.05) | <0.001 | 0.01 (0.00, 0.02) | 0.003 | 0.02 (0.01, 0.04) | 0.005 |
| SII | Ref. | — | 0.09 (0.08, 0.09) | <0.001 | 0.07 (0.06, 0.08) | <0.001 | 0.15 (0.13, 0.16) | <0.001 |
| NLR | Ref. | — | 0.08 (0.07, 0.09) | <0.001 | 0.08 (0.07, 0.09) | <0.001 | 0.16 (0.14, 0.17) | <0.001 |
| PLR | Ref. | — | 0.04 (0.03, 0.05) | <0.001 | 0.04 (0.03, 0.05) | <0.001 | 0.07 (0.06, 0.09) | <0.001 |
| LMR | Ref. | — | -0.04 (-0.04, -0.03) | <0.001 | -0.04 (-0.05, -0.03) | <0.001 | -0.04 (-0.05, -0.02) | <0.001 |

Abbreviations: SES, socioeconomic status; CI, confidence interval; FDR, false discovery rate; ISES, individual socioeconomic status; SII, systemic immune-inflammation index; NLR, neutrophil-to-lymphocyte ratio; PLR, platelet-to-lymphocyte ratio; LMR, lymphocyte-to-monocyte ratio; NSES, neighborhood socioeconomic status.

^*^ The models were adjusted for age, gender, ethnicity, family history of Alzheimer's disease, apolipoprotein E genotypes, smoking status, alcohol consumption, healthy diet, regular exercise, sleep duration, body mass index, waist circumference, systolic blood pressure, and diastolic blood pressure. FDR adjusted P values are shown in the table.

# Table S45. Associations of SES profiles with metabolites (N=174,175).^*^

|  | **Balanced-high SES subpopulation** | | **Low income-less educated subpopulation** | | | **Low income-NSES subpopulation** | | | | | **Low SES subpopulation** | |
| --- | --- | --- | --- | --- | --- | --- | --- | --- | --- | --- | --- | --- |
|  | **β (95% CI)** | **FDR** | **β (95% CI)** | **FDR** | | **β (95% CI)** | | | **FDR** | | **β (95% CI)** | **FDR** |
| Total cholesterol | Ref. | — | -0.01 (-0.02, 0.00) | 0.128 | | -0.01 (-0.02, 0.00) | | | 0.041 | | 0.00 (-0.02, 0.02) | 0.816 |
| Total cholesterol minus HDL-C | Ref. | — | -0.01 (-0.02, 0.00) | 0.215 | | -0.03 (-0.04, -0.02) | | | <0.001 | | -0.02 (-0.04, 0.00) | 0.100 |
| Remnant cholesterol (non-HDL, non-LDL -cholesterol) | Ref. | — | -0.01 (-0.02, 0.00) | 0.153 | | -0.03 (-0.05, -0.02) | | | <0.001 | | -0.01 (-0.03, 0.01) | 0.371 |
| VLDL cholesterol | Ref. | — | 0.01 (0.00, 0.02) | 0.078 | | -0.03 (-0.04, -0.02) | | | <0.001 | | 0.02 (0.00, 0.03) | 0.142 |
| Clinical LDL cholesterol | Ref. | — | -0.01 (-0.02, 0.00) | 0.115 | | -0.03 (-0.04, -0.01) | | | <0.001 | | -0.03 (-0.05, -0.01) | <0.001 |
| LDL cholesterol | Ref. | — | -0.01 (-0.02, 0.01) | 0.341 | | -0.02 (-0.04, -0.01) | | | <0.001 | | -0.02 (-0.04, 0.00) | 0.029 |
| HDL cholesterol | Ref. | — | -0.01 (-0.01, 0.00) | 0.280 | | 0.04 (0.03, 0.05) | | | <0.001 | | 0.03 (0.02, 0.05) | <0.001 |
| Total triglycerides | Ref. | — | 0.03 (0.02, 0.04) | <0.001 | | -0.01 (-0.02, 0.00) | | | 0.116 | | 0.05 (0.03, 0.07) | <0.001 |
| Triglycerides in VLDL | Ref. | — | 0.03 (0.02, 0.04) | <0.001 | | -0.01 (-0.02, 0.00) | | | 0.053 | | 0.04 (0.02, 0.06) | <0.001 |
| Triglycerides in LDL | Ref. | — | 0.02 (0.01, 0.03) | <0.001 | | 0.00 (-0.01, 0.01) | | | 0.741 | | 0.08 (0.06, 0.10) | <0.001 |
| Triglycerides in HDL | Ref. | — | 0.04 (0.03, 0.05) | <0.001 | | 0.01 (0.00, 0.02) | | | 0.183 | | 0.08 (0.06, 0.10) | <0.001 |
| Total phospholipids in lipoprotein particles | Ref. | — | 0.01 (0.00, 0.02) | 0.039 | | 0.01 (0.00, 0.02) | | | 0.199 | | 0.04 (0.03, 0.06) | <0.001 |
| Phospholipids in VLDL | Ref. | — | 0.02 (0.01, 0.03) | <0.001 | | -0.02 (-0.04, -0.01) | | | <0.001 | | 0.03 (0.01, 0.05) | 0.001 |
| Phospholipids in LDL | Ref. | — | 0.00 (-0.02, 0.01) | 0.463 | | -0.02 (-0.04, -0.01) | | | <0.001 | | -0.02 (-0.04, 0.00) | 0.026 |
|  | **Balanced-high SES subpopulation** | | **Low income-less educated subpopulation** | | | **Low income-NSES subpopulation** | | | | | **Low SES subpopulation** | |
|  | **β (95% CI)** | **FDR** | **β (95% CI)** | **FDR** | | **β (95% CI)** | | | **FDR** | | **β (95% CI)** | **FDR** |
| Phospholipids in HDL | Ref. | — | 0.01 (0.01, 0.02) | 0.004 | | 0.04 (0.03, 0.05) | | | <0.001 | | 0.06 (0.04, 0.08) | <0.001 |
| Total esterified cholesterol | Ref. | — | -0.01 (-0.02, 0.00) | 0.183 | | -0.01 (-0.02, 0.00) | | | 0.136 | | 0.00 (-0.02, 0.02) | 0.795 |
| Cholesteryl esters in VLDL | Ref. | — | 0.01 (-0.01, 0.02) | 0.356 | | -0.03 (-0.05, -0.02) | | | <0.001 | | 0.01 (-0.01, 0.03) | 0.413 |
| Cholesteryl esters in LDL | Ref. | — | 0.00 (-0.01, 0.01) | 0.881 | | -0.02 (-0.04, -0.01) | | | <0.001 | | -0.02 (-0.03, 0.00) | 0.144 |
| Cholesteryl esters in HDL | Ref. | — | 0.00 (-0.01, 0.01) | 0.680 | | 0.04 (0.03, 0.05) | | | <0.001 | | 0.03 (0.02, 0.05) | <0.001 |
| Total free cholesterol | Ref. | — | -0.01 (-0.02, 0.00) | 0.056 | | -0.02 (-0.03, -0.01) | | | <0.001 | | 0.00 (-0.02, 0.02) | 0.999 |
| Free cholesterol in VLDL | Ref. | — | 0.02 (0.01, 0.03) | 0.004 | | -0.03 (-0.04, -0.01) | | | <0.001 | | 0.03 (0.01, 0.05) | 0.009 |
| Free cholesterol in LDL | Ref. | — | -0.02 (-0.03, -0.01) | 0.003 | | -0.03 (-0.04, -0.01) | | | <0.001 | | -0.04 (-0.06, -0.02) | <0.001 |
| Free cholesterol in HDL | Ref. | — | -0.02 (-0.03, -0.01) | <0.001 | | 0.03 (0.01, 0.04) | | | <0.001 | | 0.04 (0.02, 0.06) | <0.001 |
| Total lipids in lipoprotein particles | Ref. | — | 0.01 (0.00, 0.02) | 0.138 | | -0.01 (-0.02, 0.00) | | | 0.218 | | 0.03 (0.01, 0.05) | 0.004 |
| Total lipids in VLDL | Ref. | — | 0.03 (0.02, 0.04) | <0.001 | | -0.02 (-0.03, -0.01) | | | <0.001 | | 0.03 (0.01, 0.05) | 0.002 |
| Total lipids in LDL | Ref. | — | 0.00 (-0.01, 0.01) | 0.514 | | -0.02 (-0.04, -0.01) | | | <0.001 | | -0.02 (-0.04, 0.00) | 0.102 |
| Total lipids in HDL | Ref. | — | 0.01 (0.00, 0.02) | 0.154 | | 0.04 (0.03, 0.05) | | | <0.001 | | 0.05 (0.03, 0.07) | <0.001 |
| Total concentration of lipoprotein particles | Ref. | — | 0.04 (0.03, 0.05) | <0.001 | | 0.04 (0.03, 0.06) | | | <0.001 | | 0.06 (0.04, 0.07) | <0.001 |
|  | **Balanced-high SES subpopulation** | | **Low income-less educated subpopulation** | | | **Low income-NSES subpopulation** | | | | | **Low SES subpopulation** | |
|  | **β (95% CI)** | **FDR** | **β (95% CI)** | **FDR** | | **β (95% CI)** | | | **FDR** | | **β (95% CI)** | **FDR** |
| Concentration of VLDL particles | Ref. | — | 0.01 (0.00, 0.02) | 0.145 | | -0.03 (-0.04, -0.02) | | | <0.001 | | 0.03 (0.01, 0.05) | 0.009 |
| Concentration of LDL particles | Ref. | — | -0.01 (-0.02, 0.00) | 0.066 | | -0.04 (-0.05, -0.02) | | | <0.001 | | -0.01 (-0.03, 0.01) | 0.223 |
| Concentration of HDL particles | Ref. | — | 0.05 (0.04, 0.06) | <0.001 | | 0.05 (0.04, 0.06) | | | <0.001 | | 0.06 (0.04, 0.08) | <0.001 |
| Average diameter for VLDL particles | Ref. | — | 0.05 (0.04, 0.06) | <0.001 | | 0.00 (-0.01, 0.01) | | | 0.653 | | 0.03 (0.01, 0.05) | 0.001 |
| Average diameter for LDL particles | Ref. | — | -0.01 (-0.02, 0.00) | 0.032 | | 0.00 (-0.01, 0.02) | | | 0.664 | | -0.01 (-0.03, 0.01) | 0.582 |
| Average diameter for HDL particles | Ref. | — | -0.06 (-0.07, -0.06) | <0.001 | | 0.00 (-0.01, 0.01) | | | 0.509 | | 0.01 (0.00, 0.03) | 0.102 |
| Phosphoglycerides | Ref. | — | 0.03 (0.01, 0.04) | <0.001 | | 0.02 (0.01, 0.04) | | | <0.001 | | 0.06 (0.04, 0.08) | <0.001 |
| Total cholines | Ref. | — | 0.02 (0.01, 0.03) | 0.001 | | 0.02 (0.01, 0.03) | | | <0.001 | | 0.06 (0.04, 0.07) | <0.001 |
| Phosphatidylcholines | Ref. | — | 0.01 (0.00, 0.02) | 0.197 | | 0.02 (0.01, 0.03) | | | 0.005 | | 0.05 (0.03, 0.06) | <0.001 |
| Sphingomyelins | Ref. | — | 0.01 (0.00, 0.02) | 0.247 | | 0.00 (-0.01, 0.02) | | | 0.557 | | 0.04 (0.02, 0.06) | <0.001 |
| Apolipoprotein B | Ref. | — | -0.01 (-0.02, 0.00) | 0.034 | | -0.04 (-0.05, -0.02) | | | <0.001 | | -0.02 (-0.03, 0.00) | 0.145 |
| Apolipoprotein A1 | Ref. | — | 0.03 (0.02, 0.04) | <0.001 | | 0.05 (0.04, 0.06) | | | <0.001 | | 0.06 (0.04, 0.08) | <0.001 |
| Total fatty acids | Ref. | — | 0.05 (0.03, 0.06) | <0.001 | | 0.02 (0.00, 0.03) | | | 0.012 | | 0.08 (0.06, 0.10) | <0.001 |
| Degree of unsaturation | Ref. | — | -0.08 (-0.09, -0.07) | <0.001 | | -0.07 (-0.08, -0.06) | | | <0.001 | | -0.12 (-0.14, -0.1) | <0.001 |
| Omega-3 fatty acids | Ref. | — | -0.07 (-0.08, -0.05) | <0.001 | | -0.09 (-0.11, -0.08) | | | <0.001 | | -0.06 (-0.08, -0.04) | <0.001 |
| Omega-6 fatty acids | Ref. | — | 0.02 (0.01, 0.04) | <0.001 | | 0.02 (0.01, 0.03) | | | <0.001 | | 0.04 (0.02, 0.06) | <0.001 |
| Polyunsaturated fatty acids | Ref. | — | 0.00 (-0.01, 0.01) | 0.579 | | 0.00 (-0.02, 0.01) | | | 0.532 | | 0.02 (0.00, 0.04) | 0.048 |
|  | **Balanced-high SES subpopulation** | | **Low income-less educated subpopulation** | | | **Low income-NSES subpopulation** | | | | | **Low SES subpopulation** | |
|  | **β (95% CI)** | **FDR** | **β (95% CI)** | **FDR** | | **β (95% CI)** | | | **FDR** | | **β (95% CI)** | **FDR** |
| Monounsaturated fatty acids | Ref. | — | 0.08 (0.07, 0.09) | <0.001 | | 0.04 (0.03, 0.05) | | | <0.001 | | 0.11 (0.09, 0.13) | <0.001 |
| Saturated fatty acids | Ref. | — | 0.05 (0.04, 0.06) | <0.001 | | 0.01 (0.00, 0.02) | | | 0.073 | | 0.08 (0.06, 0.10) | <0.001 |
| Linoleic acid | Ref. | — | 0.00 (-0.01, 0.01) | 0.725 | | 0.02 (0.01, 0.03) | | | 0.003 | | 0.03 (0.01, 0.05) | 0.011 |
| Docosahexaenoic acid | Ref. | — | -0.09 (-0.10, -0.08) | <0.001 | | -0.09 (-0.10, -0.08) | | | <0.001 | | -0.08 (-0.10, -0.06) | <0.001 |
| Alanine | Ref. | — | -0.06 (-0.07, -0.05) | <0.001 | | -0.05 (-0.06, -0.04) | | | <0.001 | | -0.08 (-0.10, -0.06) | <0.001 |
| Glutamine | Ref. | — | 0.03 (0.02, 0.04) | <0.001 | | 0.02 (0.00, 0.03) | | | 0.023 | | 0.01 (-0.01, 0.03) | 0.419 |
| Glycine | Ref. | — | -0.02 (-0.03, -0.01) | <0.001 | | 0.00 (-0.01, 0.02) | | | 0.625 | | 0.02 (0.00, 0.04) | 0.069 |
| Histidine | Ref. | — | -0.01 (-0.02, 0.01) | 0.424 | | -0.01 (-0.02, 0.00) | | | 0.184 | | -0.06 (-0.08, -0.04) | <0.001 |
| Total concentration of branched-chain amino acids (leucine + isoleucine + valine) | Ref. | — | -0.07 (-0.08, -0.06) | <0.001 | | -0.08 (-0.09, -0.06) | | | <0.001 | | -0.16 (-0.18, -0.14) | <0.001 |
| Isoleucine | Ref. | — | -0.05 (-0.06, -0.04) | <0.001 | | -0.05 (-0.06, -0.04) | | | <0.001 | | -0.13 (-0.15, -0.11) | <0.001 |
| Leucine | Ref. | — | -0.04 (-0.05, -0.03) | <0.001 | | -0.06 (-0.07, -0.05) | | | <0.001 | | -0.14 (-0.16, -0.12) | <0.001 |
| Valine | Ref. | — | -0.09 (-0.10, -0.08) | <0.001 | | -0.09 (-0.1, -0.07) | | | <0.001 | | -0.17 (-0.19, -0.15) | <0.001 |
| Phenylalanine | Ref. | — | 0.00 (-0.01, 0.02) | 0.538 | | -0.01 (-0.02, 0.00) | | | 0.245 | | -0.09 (-0.11, -0.07) | <0.001 |
| Tyrosine | Ref. | — | -0.03 (-0.04, -0.02) | <0.001 | | -0.04 (-0.05, -0.02) | | | <0.001 | | -0.10 (-0.12, -0.08) | <0.001 |
| Glucose | Ref. | — | 0.03 (0.02, 0.04) | <0.001 | | 0.05 (0.04, 0.06) | | | <0.001 | | 0.05 (0.04, 0.07) | <0.001 |
|  | **Balanced-high SES subpopulation** | | **Low income-less educated subpopulation** | | | **Low income-NSES subpopulation** | | | | | **Low SES subpopulation** | |
|  | **β (95% CI)** | **FDR** | **β (95% CI)** | **FDR** | | **β (95% CI)** | | | **FDR** | | **β (95% CI)** | **FDR** |
| Lactate | Ref. | — | 0.00 (-0.01, 0.01) | 0.939 | | -0.05 (-0.06, -0.04) | | | <0.001 | | 0.04 (0.02, 0.06) | <0.001 |
| Pyruvate | Ref. | — | -0.02 (-0.04, -0.01) | <0.001 | | -0.01 (-0.02, 0.01) | | | 0.486 | | 0.04 (0.02, 0.06) | 0.001 |
| Citrate | Ref. | — | 0.02 (0.00, 0.03) | 0.010 | | 0.02 (0.01, 0.04) | | | <0.001 | | 0.02 (0.00, 0.04) | 0.029 |
| 3-Hydroxybutyrate | Ref. | — | 0.04 (0.02, 0.05) | <0.001 | | 0.06 (0.04, 0.07) | | | <0.001 | | 0.14 (0.12, 0.16) | <0.001 |
| Acetate | Ref. | — | -0.04 (-0.06, -0.03) | <0.001 | | 0.02 (0.01, 0.04) | | | <0.001 | | -0.03 (-0.05, -0.01) | 0.005 |
| Acetoacetate | Ref. | — | 0.02 (0.01, 0.03) | <0.001 | | 0.08 (0.07, 0.09) | | | <0.001 | | 0.10 (0.08, 0.12) | <0.001 |
| Acetone | Ref. | — | -0.02 (-0.03, -0.01) | <0.001 | | 0.03 (0.01, 0.04) | | | <0.001 | | 0.07 (0.05, 0.09) | <0.001 |
| Creatinine | Ref. | — | 0.02 (0.01, 0.03) | <0.001 | | -0.03 (-0.04, -0.02) | | | <0.001 | | -0.03 (-0.05, -0.02) | <0.001 |
| Albumin | Ref. | — | -0.04 (-0.05, -0.03) | <0.001 | | -0.06 (-0.07, -0.04) | | | <0.001 | | -0.04 (-0.06, -0.02) | <0.001 |
| Glycoprotein acetyls | Ref. | — | 0.11 (0.10, 0.13) | <0.001 | | 0.06 (0.05, 0.07) | | | <0.001 | | 0.14 (0.12, 0.16) | <0.001 |
| Concentration of chylomicrons and extremely large VLDL particles | Ref. | — | 0.04 (0.03, 0.05) | <0.001 | | 0.01 (-0.01, 0.02) | | | 0.387 | | 0.07 (0.05, 0.08) | <0.001 |
| Total lipids in chylomicrons and extremely large VLDL | Ref. | — | 0.04 (0.03, 0.06) | <0.001 | | 0.00 (-0.01, 0.02) | | | 0.566 | | 0.06 (0.04, 0.08) | <0.001 |
| Phospholipids in chylomicrons and extremely large VLDL | Ref. | — | 0.05 (0.04, 0.06) | <0.001 | | 0.01 (0.000, 0.02) | | | 0.183 | | 0.07 (0.05, 0.09) | <0.001 |
|  | **Balanced-high SES subpopulation** | | **Low income-less educated subpopulation** | | | **Low income-NSES subpopulation** | | | | | **Low SES subpopulation** | |
|  | **β (95% CI)** | **FDR** | **β (95% CI)** | **FDR** | | **β (95% CI)** | | | **FDR** | | **β (95% CI)** | **FDR** |
| Cholesterol in chylomicrons and extremely large VLDL | Ref. | — | 0.06 (0.05, 0.07) | <0.001 | | 0.01 (0.00, 0.02) | | | 0.099 | | 0.08 (0.06, 0.10) | <0.001 |
| Cholesteryl esters in chylomicrons and extremely large VLDL | Ref. | — | 0.06 (0.05, 0.07) | <0.001 | | 0.01 (0.00, 0.02) | | | 0.045 | | 0.08 (0.06, 0.10) | <0.001 |
| Free cholesterol in chylomicrons and extremely large VLDL | Ref. | — | 0.05 (0.04, 0.06) | <0.001 | | 0.01 (0.00, 0.02) | | | 0.192 | | 0.07 (0.05, 0.09) | <0.001 |
| Triglycerides in chylomicrons and extremely large VLDL | Ref. | — | 0.04 (0.03, 0.05) | <0.001 | | 0.00 (-0.01, 0.01) | | | 0.813 | | 0.06 (0.04, 0.07) | <0.001 |
| Concentration of very large VLDL particles | Ref. | — | 0.04 (0.03, 0.05) | <0.001 | | -0.01 (-0.02, 0.00) | | | 0.164 | | 0.05 (0.03, 0.06) | <0.001 |
| Total lipids in very large VLDL | Ref. | — | 0.04 (0.03, 0.05) | <0.001 | | -0.01 (-0.02, 0.00) | | | 0.079 | | 0.04 (0.02, 0.06) | <0.001 |
| Phospholipids in very large VLDL | Ref. | — | 0.03 (0.02, 0.04) | <0.001 | | -0.01 (-0.02, 0.00) | | | 0.073 | | 0.04 (0.03, 0.06) | <0.001 |
| Cholesterol in very large VLDL | Ref. | — | 0.04 (0.03, 0.05) | <0.001 | | -0.02 (-0.03, 0.00) | | | 0.015 | | 0.04 (0.02, 0.06) | <0.001 |
| Cholesteryl esters in very large VLDL | Ref. | — | 0.04 (0.03, 0.05) | <0.001 | | -0.02 (-0.03, 0.00) | | | 0.009 | | 0.03 (0.01, 0.05) | 0.002 |
| Free cholesterol in very large VLDL | Ref. | — | 0.03 (0.02, 0.04) | <0.001 | | -0.01 (-0.03, 0.00) | | | 0.036 | | 0.04 (0.03, 0.06) | <0.001 |
|  | **Balanced-high SES subpopulation** | | **Low income-less educated subpopulation** | | | **Low income-NSES subpopulation** | | | | | **Low SES subpopulation** | |
|  | **β (95% CI)** | **FDR** | **β (95% CI)** | **FDR** | | **β (95% CI)** | | | **FDR** | | **β (95% CI)** | **FDR** |
| Triglycerides in very large VLDL | Ref. | — | 0.04 (0.03, 0.05) | <0.001 | | -0.01 (-0.02, 0.00) | | | 0.220 | | 0.04 (0.03, 0.06) | <0.001 |
| Concentration of large VLDL particles | Ref. | — | 0.03 (0.02, 0.04) | <0.001 | | -0.01 (-0.03, 0.00) | | | 0.020 | | 0.04 (0.02, 0.06) | <0.001 |
| Total lipids in large VLDL | Ref. | — | 0.03 (0.02, 0.04) | <0.001 | | -0.02 (-0.03, -0.01) | | | 0.002 | | 0.03 (0.01, 0.05) | 0.003 |
| Phospholipids in large VLDL | Ref. | — | 0.03 (0.02, 0.04) | <0.001 | | -0.01 (-0.03, 0.00) | | | 0.021 | | 0.04 (0.02, 0.06) | <0.001 |
| Cholesterol in large VLDL | Ref. | — | 0.03 (0.02, 0.04) | <0.001 | | -0.02 (-0.04, -0.01) | | | <0.001 | | 0.03 (0.01, 0.05) | 0.004 |
| Cholesteryl esters in large VLDL | Ref. | — | 0.02 (0.01, 0.03) | <0.001 | | -0.03 (-0.04, -0.02) | | | <0.001 | | 0.02 (0.00, 0.04) | 0.032 |
| Free cholesterol in large VLDL | Ref. | — | 0.03 (0.02, 0.04) | <0.001 | | -0.02 (-0.03, 0.00) | | | 0.010 | | 0.04 (0.02, 0.05) | <0.001 |
| Triglycerides in large VLDL | Ref. | — | 0.03 (0.02, 0.04) | <0.001 | | -0.02 (-0.03, 0.00) | | | 0.008 | | 0.03 (0.01, 0.05) | 0.005 |
| Concentration of medium VLDL particles | Ref. | — | 0.01 (0.00, 0.02) | 0.154 | | -0.03 (-0.04, -0.02) | | | <0.001 | | 0.01 (-0.01, 0.03) | 0.302 |
| Total lipids in medium VLDL | Ref. | — | 0.01 (0.00, 0.02) | 0.050 | | -0.03 (-0.04, -0.02) | | | <0.001 | | 0.01 (-0.01, 0.03) | 0.288 |
| Phospholipids in medium VLDL | Ref. | — | 0.00 (-0.01, 0.01) | 0.525 | | -0.03 (-0.05, -0.02) | | | <0.001 | | 0.01 (-0.01, 0.03) | 0.584 |
| Cholesterol in medium VLDL | Ref. | — | -0.02 (-0.03, 0.00) | 0.009 | | -0.04 (-0.05, -0.03) | | | <0.001 | | -0.02 (-0.04, 0.00) | 0.063 |
| Cholesteryl esters in medium VLDL | Ref. | — | -0.02 (-0.03, -0.01) | <0.001 | | -0.04 (-0.05, -0.03) | | | <0.001 | | -0.03 (-0.05, -0.01) | 0.002 |
| Free cholesterol in medium VLDL | Ref. | — | 0.00 (-0.01, 0.01) | 0.608 | | -0.04 (-0.05, -0.03) | | | <0.001 | | 0.00 (-0.02, 0.02) | 0.868 |
|  | **Balanced-high SES subpopulation** | | **Low income-less educated subpopulation** | | | **Low income-NSES subpopulation** | | | | | **Low SES subpopulation** | |
|  | **β (95% CI)** | **FDR** | **β (95% CI)** | **FDR** | | **β (95% CI)** | | | **FDR** | | **β (95% CI)** | **FDR** |
| Triglycerides in medium VLDL | Ref. | — | 0.03 (0.02, 0.04) | <0.001 | | -0.02 (-0.03, -0.01) | | | 0.007 | | 0.03 (0.01, 0.05) | 0.003 |
| Concentration of small VLDL particles | Ref. | — | 0.01 (0.00, 0.03) | 0.015 | | -0.02 (-0.04, -0.01) | | | <0.001 | | 0.03 (0.01, 0.05) | 0.001 |
| Total lipids in small VLDL | Ref. | — | 0.02 (0.01, 0.03) | 0.006 | | -0.03 (-0.04, -0.01) | | | <0.001 | | 0.03 (0.01, 0.05) | 0.006 |
| Phospholipids in small VLDL | Ref. | — | 0.01 (0.00, 0.02) | 0.278 | | -0.03 (-0.04, -0.02) | | | <0.001 | | 0.01 (-0.01, 0.03) | 0.235 |
| Cholesterol in small VLDL | Ref. | — | 0.01 (-0.01, 0.02) | 0.361 | | -0.03 (-0.05, -0.02) | | | <0.001 | | 0.01 (-0.01, 0.03) | 0.497 |
| Cholesteryl esters in small VLDL | Ref. | — | 0.01 (0.00, 0.02) | 0.091 | | -0.03 (-0.05, -0.02) | | | <0.001 | | 0.01 (-0.01, 0.03) | 0.212 |
| Free cholesterol in small VLDL | Ref. | — | 0.00 (-0.01, 0.01) | 0.665 | | -0.04 (-0.05, -0.02) | | | <0.001 | | 0.00 (-0.02, 0.02) | 0.855 |
| Triglycerides in small VLDL | Ref. | — | 0.03 (0.02, 0.04) | <0.001 | | -0.01 (-0.02, 0.00) | | | 0.120 | | 0.05 (0.03, 0.07) | <0.001 |
| Concentration of very small VLDL particles | Ref. | — | -0.02 (-0.03, 0.00) | 0.007 | | -0.04 (-0.05, -0.02) | | | <0.001 | | 0.02 (0.00, 0.04) | 0.113 |
| Total lipids in very small VLDL | Ref. | — | -0.01 (-0.03, 0.00) | 0.016 | | -0.04 (-0.05, -0.02) | | | <0.001 | | 0.02 (0.00, 0.04) | 0.034 |
| Phospholipids in very small VLDL | Ref. | — | -0.01 (-0.02, 0.00) | 0.047 | | -0.03 (-0.05, -0.02) | | | <0.001 | | 0.03 (0.01, 0.05) | 0.002 |
| Cholesterol in very small VLDL | Ref. | — | -0.03 (-0.04, -0.02) | <0.001 | | -0.04 (-0.05, -0.03) | | | <0.001 | | -0.01 (-0.03, 0.01) | 0.481 |
| Cholesteryl esters in very small VLDL | Ref. | — | -0.03 (-0.04, -0.02) | <0.001 | | -0.04 (-0.05, -0.03) | | | <0.001 | | -0.02 (-0.04, 0.00) | 0.108 |
|  | **Balanced-high SES subpopulation** | | **Low income-less educated subpopulation** | | | **Low income-NSES subpopulation** | | | | | **Low SES subpopulation** | |
|  | **β (95% CI)** | **FDR** | **β (95% CI)** | **FDR** | | **β (95% CI)** | | | **FDR** | | **β (95% CI)** | **FDR** |
| Triglycerides in very small VLDL | Ref. | — | 0.02 (0.01, 0.03) | 0.001 | | -0.01 (-0.02, 0.01) | | | 0.352 | | 0.07 (0.05, 0.09) | <0.001 |
| Concentration of IDL particles | Ref. | — | -0.02 (-0.04, -0.01) | <0.001 | | -0.04 (-0.05, -0.03) | | | <0.001 | | -0.03 (-0.05, -0.01) | 0.001 |
| Total lipids in IDL | Ref. | — | -0.03 (-0.04, -0.01) | <0.001 | | -0.03 (-0.04, -0.02) | | | <0.001 | | -0.02 (-0.04, -0.01) | 0.015 |
| Phospholipids in IDL | Ref. | — | -0.03 (-0.04, -0.02) | <0.001 | | -0.03 (-0.04, -0.02) | | | <0.001 | | -0.02 (-0.03, 0.00) | 0.117 |
| Cholesterol in IDL | Ref. | — | -0.03 (-0.04, -0.02) | <0.001 | | -0.03 (-0.04, -0.02) | | | <0.001 | | -0.04 (-0.05, -0.02) | <0.001 |
| Cholesteryl esters in IDL | Ref. | — | -0.03 (-0.04, -0.02) | <0.001 | | -0.03 (-0.04, -0.02) | | | <0.001 | | -0.04 (-0.06, -0.02) | <0.001 |
| Free cholesterol in IDL | Ref. | — | -0.03 (-0.04, -0.02) | <0.001 | | -0.03 (-0.04, -0.02) | | | <0.001 | | -0.03 (-0.05, -0.01) | 0.004 |
| Triglycerides in IDL | Ref. | — | 0.01 (0.00, 0.03) | 0.015 | | 0.00 (-0.02, 0.01) | | | 0.646 | | 0.07 (0.06, 0.09) | <0.001 |
| Concentration of large LDL particles | Ref. | — | -0.01 (-0.03, 0.00) | 0.014 | | -0.03 (-0.05, -0.02) | | | <0.001 | | -0.01 (-0.03, 0.01) | 0.221 |
| Total lipids in large LDL | Ref. | — | -0.01 (-0.02, 0.00) | 0.150 | | -0.02 (-0.03, -0.01) | | | <0.001 | | -0.02 (-0.04, 0.00) | 0.071 |
| Phospholipids in large LDL | Ref. | — | 0.00 (-0.01, 0.01) | 0.600 | | -0.02 (-0.03, -0.01) | | | <0.001 | | -0.02 (-0.04, 0.00) | 0.028 |
| Cholesterol in large LDL | Ref. | — | -0.01 (-0.02, 0.00) | 0.043 | | -0.02 (-0.04, -0.01) | | | <0.001 | | -0.02 (-0.04, -0.01) | 0.016 |
| Cholesteryl esters in large LDL | Ref. | — | -0.01 (-0.02, 0.00) | 0.133 | | -0.02 (-0.04, -0.01) | | | <0.001 | | -0.02 (-0.04, 0.00) | 0.049 |
| Free cholesterol in large LDL | Ref. | — | -0.02 (-0.03, -0.01) | 0.001 | | -0.02 (-0.04, -0.01) | | | <0.001 | | -0.03 (-0.05, -0.01) | 0.001 |
| Triglycerides in large LDL | Ref. | — | 0.02 (0.01, 0.03) | 0.001 | | 0.00 (-0.01, 0.02) | | | 0.664 | | 0.08 (0.06, 0.10) | <0.001 |
|  | **Balanced-high SES subpopulation** | | **Low income-less educated subpopulation** | | | **Low income-NSES subpopulation** | | | | | **Low SES subpopulation** | |
|  | **β (95% CI)** | **FDR** | **β (95% CI)** | **FDR** | | **β (95% CI)** | | | **FDR** | | **β (95% CI)** | **FDR** |
| Concentration of medium LDL particles | Ref. | — | 0.00 (-0.01, 0.01) | 0.982 | | -0.03 (-0.04, -0.02) | | | <0.001 | | -0.01 (-0.03, 0.01) | 0.285 |
| Total lipids in medium LDL | Ref. | — | 0.01 (0.00, 0.02) | 0.071 | | -0.02 (-0.03, -0.01) | | | 0.002 | | -0.01 (-0.03, 0.01) | 0.407 |
| Phospholipids in medium LDL | Ref. | — | 0.01 (0.00, 0.02) | 0.157 | | -0.02 (-0.03, -0.01) | | | 0.003 | | -0.01 (-0.03, 0.01) | 0.198 |
| Cholesterol in medium LDL | Ref. | — | 0.01 (0.00, 0.02) | 0.086 | | -0.02 (-0.03, -0.01) | | | <0.001 | | -0.01 (-0.03, 0.01) | 0.203 |
| Cholesteryl esters in medium LDL | Ref. | — | 0.02 (0.01, 0.03) | 0.004 | | -0.02 (-0.03, -0.01) | | | 0.002 | | 0.00 (-0.02, 0.02) | 0.740 |
| Free cholesterol in medium LDL | Ref. | — | -0.01 (-0.02, 0.00) | 0.245 | | -0.02 (-0.04, -0.01) | | | <0.001 | | -0.04 (-0.06, -0.02) | <0.001 |
| Triglycerides in medium LDL | Ref. | — | 0.02 (0.01, 0.03) | <0.001 | | 0.00 (-0.01, 0.02) | | | 0.619 | | 0.08 (0.06, 0.09) | <0.001 |
| Concentration of small LDL particles | Ref. | — | -0.01 (-0.03, 0.00) | 0.013 | | -0.04 (-0.05, -0.03) | | | <0.001 | | -0.01 (-0.03, 0.01) | 0.219 |
| Total lipids in small LDL | Ref. | — | -0.01 (-0.02, 0.00) | 0.084 | | -0.03 (-0.04, -0.02) | | | <0.001 | | -0.02 (-0.04, 0.00) | 0.095 |
| Phospholipids in small LDL | Ref. | — | -0.04 (-0.05, -0.03) | <0.001 | | -0.04 (-0.05, -0.03) | | | <0.001 | | -0.04 (-0.06, -0.02) | <0.001 |
| Cholesterol in small LDL | Ref. | — | 0.00 (-0.01, 0.01) | 0.651 | | -0.03 (-0.04, -0.02) | | | <0.001 | | -0.02 (-0.04, 0.00) | 0.084 |
| Cholesteryl esters in small LDL | Ref. | — | 0.010 (0.00, 0.02) | 0.203 | | -0.02 (-0.03, -0.01) | | | <0.001 | | 0.00 (-0.02, 0.02) | 0.891 |
| Free cholesterol in small LDL | Ref. | — | -0.03 (-0.04, -0.02) | <0.001 | | -0.04 (-0.05, -0.03) | | | <0.001 | | -0.06 (-0.08, -0.04) | <0.001 |
| Triglycerides in small LDL | Ref. | — | 0.02 (0.01, 0.04) | <0.001 | | 0.00 (-0.01, 0.01) | | | 0.881 | | 0.07 (0.05, 0.09) | <0.001 |
|  | **Balanced-high SES subpopulation** | | **Low income-less educated subpopulation** | | | **Low income-NSES subpopulation** | | | | | **Low SES subpopulation** | |
|  | **β (95% CI)** | **FDR** | **β (95% CI)** | **FDR** | | **β (95% CI)** | | | **FDR** | | **β (95% CI)** | **FDR** |
| Concentration of very large HDL particles | Ref. | — | -0.09 (-0.10, -0.08) | <0.001 | | -0.01 (-0.02, 0.00) | | | 0.066 | | 0.01 (-0.01, 0.02) | 0.474 |
| Total lipids in very large HDL | Ref. | — | -0.09 (-0.10, -0.08) | <0.001 | | -0.01 (-0.02, 0.00) | | | 0.052 | | 0.00 (-0.02, 0.02) | 0.972 |
| Phospholipids in very large HDL | Ref. | — | -0.09 (-0.10, -0.08) | <0.001 | | -0.01 (-0.02, 0.00) | | | 0.130 | | 0.00 (-0.01, 0.02) | 0.738 |
| Cholesterol in very large HDL | Ref. | — | -0.09 (-0.10, -0.08) | <0.001 | | -0.01 (-0.02, 0.00) | | | 0.041 | | -0.01 (-0.03, 0.01) | 0.397 |
| Cholesteryl esters in very large HDL | Ref. | — | -0.08 (-0.09, -0.07) | <0.001 | | -0.01 (-0.02, 0.00) | | | 0.167 | | 0.00 (-0.02, 0.01) | 0.653 |
| Free cholesterol in very large HDL | Ref. | — | -0.10 (-0.11, -0.09) | <0.001 | | -0.03 (-0.04, -0.01) | | | <0.001 | | -0.02 (-0.04, 0.00) | 0.042 |
| Triglycerides in very large HDL | Ref. | — | -0.02 (-0.03, -0.01) | <0.001 | | -0.01 (-0.02, 0.00) | | | 0.097 | | 0.07 (0.05, 0.09) | <0.001 |
| Concentration of large HDL particles | Ref. | — | -0.05 (-0.06, -0.04) | | <0.001 | | 0.01 (0.00, 0.02) | 0.016 | | 0.02 (0.01, 0.04) | | 0.010 |
| Total lipids in large HDL | Ref. | — | -0.05 (-0.06, -0.04) | | <0.001 | | 0.01 (0.00, 0.02) | 0.010 | | 0.02 (0.00, 0.04) | | 0.020 |
| Phospholipids in large HDL | Ref. | — | -0.04 (-0.05, -0.03) | | <0.001 | | 0.02 (0.01, 0.03) | <0.001 | | 0.03 (0.01, 0.04) | | 0.003 |
| Cholesterol in large HDL | Ref. | — | -0.05 (-0.06, -0.04) | | <0.001 | | 0.01 (0.00, 0.02) | 0.041 | | 0.01 (0.00, 0.03) | | 0.183 |
| Cholesteryl esters in large HDL | Ref. | — | -0.05 (-0.06, -0.04) | | <0.001 | | 0.01 (0.00, 0.02) | 0.024 | | 0.01 (0.00, 0.03) | | 0.188 |
| Free cholesterol in large HDL | Ref. | — | -0.06 (-0.07, -0.05) | | <0.001 | | 0.01 (0.00, 0.02) | 0.130 | | 0.02 (0.00, 0.03) | | 0.088 |
| Triglycerides in large HDL | Ref. | — | -0.01 (-0.02, 0.00) | | 0.266 | | 0.00 (-0.01, 0.02) | 0.486 | | 0.07 (0.05, 0.09) | | <0.001 |
|  | **Balanced-high SES subpopulation** | | **Low income-less educated subpopulation** | | | | **Low income-NSES subpopulation** | | | **Low SES subpopulation** | | |
|  | **β (95% CI)** | **FDR** | **β (95% CI)** | | **FDR** | | **β (95% CI)** | **FDR** | | **β (95% CI)** | | **FDR** |
| Concentration of medium HDL particles | Ref. | — | 0.04 (0.03, 0.05) | | <0.001 | | 0.05 (0.04, 0.07) | <0.001 | | 0.07 (0.05, 0.08) | | <0.001 |
| Total lipids in medium HDL | Ref. | — | 0.04 (0.03, 0.05) | | <0.001 | | 0.06 (0.04, 0.07) | <0.001 | | 0.07 (0.05, 0.09) | | <0.001 |
| Phospholipids in medium HDL | Ref. | — | 0.05 (0.04, 0.06) | | <0.001 | | 0.06 (0.04, 0.07) | <0.001 | | 0.07 (0.06, 0.09) | | <0.001 |
| Cholesterol in medium HDL | Ref. | — | 0.03 (0.02, 0.04) | | <0.001 | | 0.05 (0.04, 0.07) | <0.001 | | 0.05 (0.04, 0.07) | | <0.001 |
| Cholesteryl esters in medium HDL | Ref. | — | 0.03 (0.03, 0.04) | | <0.001 | | 0.06 (0.05, 0.07) | <0.001 | | 0.05 (0.04, 0.07) | | <0.001 |
| Free cholesterol in medium HDL | Ref. | — | 0.02 (0.01, 0.03) | | <0.001 | | 0.04 (0.03, 0.06) | <0.001 | | 0.06 (0.04, 0.07) | | <0.001 |
| Triglycerides in medium HDL | Ref. | — | 0.05 (0.04, 0.07) | | <0.001 | | 0.02 (0.00, 0.03) | 0.013 | | 0.08 (0.06, 0.10) | | <0.001 |
| Concentration of small HDL particles | Ref. | — | 0.10 (0.08, 0.11) | | <0.001 | | 0.05 (0.04, 0.06) | <0.001 | | 0.05 (0.03, 0.07) | | <0.001 |
| Total lipids in small HDL | Ref. | — | 0.10 (0.09, 0.11) | | <0.001 | | 0.06 (0.04, 0.07) | <0.001 | | 0.07 (0.05, 0.09) | | <0.001 |
| Phospholipids in small HDL | Ref. | — | 0.10 (0.09, 0.11) | | <0.001 | | 0.06 (0.05, 0.07) | <0.001 | | 0.08 (0.06, 0.10) | | <0.001 |
| Cholesterol in small HDL | Ref. | — | 0.09 (0.08, 0.10) | | <0.001 | | 0.05 (0.04, 0.07) | <0.001 | | 0.05 (0.03, 0.07) | | <0.001 |
| Cholesteryl esters in small HDL | Ref. | — | 0.10 (0.09, 0.11) | | <0.001 | | 0.06 (0.04, 0.07) | <0.001 | | 0.04 (0.02, 0.06) | | <0.001 |
| Free cholesterol in small HDL | Ref. | — | 0.06 (0.05, 0.07) | | <0.001 | | 0.04 (0.03, 0.05) | <0.001 | | 0.06 (0.04, 0.08) | | <0.001 |
| Triglycerides in small HDL | Ref. | — | 0.06 (0.05, 0.07) | | <0.001 | | 0.01 (0.00, 0.02) | 0.258 | | 0.07 (0.05, 0.09) | | <0.001 |

Abbreviations: SES, socioeconomic status; CI, confidence interval; FDR, false discovery rate; NSES, neighborhood socioeconomic status; HDL, high-density lipoprotein; LDL, low-density lipoprotein; VLDL, very low-density lipoprotein.

^*^ The models were adjusted for age, gender, ethnicity, family history of Alzheimer's disease, apolipoprotein E genotypes, smoking status, alcohol consumption, healthy diet, regular exercise, sleep duration, body mass index, waist circumference, systolic blood pressure, and diastolic blood pressure. FDR adjusted P values are shown in the table.

# Table S46. Associations of inflammatory markers with the risk of dementia (N=303,981).^*^

| **Inflammation markers** | **HR (95% CI)** | **FDR** |
| --- | --- | --- |
| Leukocyte count | 1.05 (1.02, 1.09) | 0.008 |
| Neutrophil count | 1.10 (1.06, 1.14) | <0.001 |
| Neutrophil percentage | 1.13 (1.09, 1.18) | <0.001 |
| Monocyte count | 0.99 (0.95, 1.02) | 0.501 |
| Monocyte percentage | 0.95 (0.92, 0.98) | 0.003 |
| Lymphocyte count | 0.93 (0.90, 0.96) | <0.001 |
| Lymphocyte percentage | 0.89 (0.86, 0.92) | <0.001 |
| C-reactive protein | 1.00 (0.96, 1.04) | 0.909 |
| Platelet count | 1.01 (0.97, 1.04) | 0.684 |
| SII | 1.11 (1.08, 1.15) | <0.001 |
| NLR | 1.13 (1.09, 1.17) | <0.001 |
| PLR | 1.07 (1.03, 1.10) | <0.001 |
| LMR | 0.96 (0.92, 0.99) | 0.015 |

Abbreviations: HR, hazard ratio; CI, confidence interval; FDR, false discovery rate; SII, systemic immune-inflammation index; NLR, neutrophil-to-lymphocyte ratio; PLR, platelet-to-lymphocyte ratio; LMR, lymphocyte-to-monocyte ratio.

^*^ The models were adjusted for age, gender, ethnicity, family history of Alzheimer's disease, apolipoprotein E genotypes, smoking status, alcohol consumption, healthy diet, regular exercise, sleep duration, body mass index, waist circumference, systolic blood pressure, and diastolic blood pressure. FDR adjusted P values are shown in the table.

# Table S47. Associations of metabolites with the risk of dementia (N=174,175).^*^

| **Metabolites** | **HR (95% CI)** | **FDR** |
| --- | --- | --- |
| Total cholesterol | 0.97 (0.93, 1.02) | 0.349 |
| Total cholesterol minus HDL-C | 0.95 (0.91, 1.00) | 0.063 |
| Remnant cholesterol (non-HDL, non-LDL -cholesterol) | 0.96 (0.92, 1.00) | 0.122 |
| VLDL cholesterol | 0.95 (0.90, 0.99) | 0.035 |
| Clinical LDL cholesterol | 0.95 (0.91, 1.00) | 0.075 |
| LDL cholesterol | 0.95 (0.91, 0.99) | 0.049 |
| HDL cholesterol | 1.07 (1.02, 1.13) | 0.02 |
| Total triglycerides | 0.93 (0.89, 0.97) | 0.011 |
| Triglycerides in VLDL | 0.92 (0.88, 0.96) | 0.004 |
| Triglycerides in LDL | 0.98 (0.94, 1.03) | 0.469 |
| Triglycerides in HDL | 0.98 (0.94, 1.03) | 0.541 |
| Total phospholipids in lipoprotein particles | 0.99 (0.95, 1.04) | 0.808 |
| Phospholipids in VLDL | 0.94 (0.89, 0.98) | 0.016 |
| Phospholipids in LDL | 0.95 (0.91, 1.00) | 0.062 |
| Phospholipids in HDL | 1.07 (1.01, 1.12) | 0.033 |
| Total esterified cholesterol | 0.98 (0.93, 1.02) | 0.412 |
| Cholesteryl esters in VLDL | 0.95 (0.91, 1.00) | 0.072 |
| Cholesteryl esters in LDL | 0.95 (0.90, 0.99) | 0.035 |
| Cholesteryl esters in HDL | 1.07 (1.02, 1.13) | 0.020 |
| Total free cholesterol | 0.97 (0.93, 1.02) | 0.262 |
| Free cholesterol in VLDL | 0.94 (0.90, 0.98) | 0.019 |
| Free cholesterol in LDL | 0.96 (0.92, 1.01) | 0.136 |
| Free cholesterol in HDL | 1.08 (1.02, 1.13) | 0.016 |
| Total lipids in lipoprotein particles | 0.96 (0.92, 1.01) | 0.151 |
| Total lipids in VLDL | 0.92 (0.88, 0.97) | 0.005 |
| Total lipids in LDL | 0.95 (0.91, 0.99) | 0.050 |
| Total lipids in HDL | 1.07 (1.01, 1.13) | 0.030 |
| Total concentration of lipoprotein particles | 1.01 (0.97, 1.06) | 0.656 |
| Concentration of VLDL particles | 0.95 (0.90, 0.99) | 0.036 |
| Concentration of LDL particles | 0.95 (0.91, 1.00) | 0.063 |
| Concentration of HDL particles | 1.02 (0.97, 1.08) | 0.421 |
| Average diameter for VLDL particles | 0.9 (0.85, 0.94) | <0.001 |
| Average diameter for LDL particles | 1.03 (0.98, 1.08) | 0.294 |
| Average diameter for HDL particles | 1.12 (1.06, 1.18) | <0.001 |
| Phosphoglycerides | 1.00 (0.95, 1.05) | 0.897 |
| Total cholines | 1.00 (0.95, 1.05) | 0.990 |
| Phosphatidylcholines | 0.99 (0.94, 1.04) | 0.734 |
| Sphingomyelins | 1.03 (0.98, 1.08) | 0.359 |
| Apolipoprotein B | 0.95 (0.91, 1.00) | 0.061 |
| Apolipoprotein A1 | 1.05 (1.00, 1.11) | 0.085 |
| Total fatty acids | 0.96 (0.92, 1.01) | 0.134 |
| Degree of unsaturation | 0.94 (0.89, 0.98) | 0.017 |
| Omega-3 fatty acids | 0.92 (0.88, 0.96) | 0.002 |
| Omega-6 fatty acids | 0.95 (0.91, 1.00) | 0.061 |
| Polyunsaturated fatty acids | 0.93 (0.89, 0.98) | 0.016 |
| Monounsaturated fatty acids | 0.97 (0.93, 1.02) | 0.265 |
| Saturated fatty acids | 0.98 (0.94, 1.03) | 0.553 |
| Linoleic acid | 0.95 (0.91, 0.99) | 0.046 |
| Docosahexaenoic acid | 0.93 (0.89, 0.98) | 0.012 |
| **Metabolites** | **HR (95% CI)** | **FDR** |
| Alanine | 0.92 (0.88, 0.96) | 0.002 |
| Glutamine | 1.01 (0.96, 1.05) | 0.791 |
| Glycine | 1.03 (0.98, 1.08) | 0.359 |
| Histidine | 0.96 (0.92, 1.01) | 0.136 |
| Total concentration of branched-chain amino acids (leucine + isoleucine + valine) | 0.86 (0.82, 0.90) | <0.001 |
| Isoleucine | 0.90 (0.86, 0.94) | <0.001 |
| Leucine | 0.87 (0.83, 0.92) | <0.001 |
| Valine | 0.84 (0.80, 0.89) | <0.001 |
| Phenylalanine | 0.94 (0.90, 0.98) | 0.017 |
| Tyrosine | 0.93 (0.89, 0.98) | 0.013 |
| Glucose | 1.06 (1.00, 1.12) | 0.063 |
| Lactate | 0.96 (0.92, 1.00) | 0.122 |
| Pyruvate | 0.97 (0.93, 1.01) | 0.245 |
| Citrate | 0.99 (0.95, 1.03) | 0.693 |
| 3-Hydroxybutyrate | 1.07 (1.03, 1.11) | 0.005 |
| Acetate | 1.00 (0.96, 1.03) | 0.897 |
| Acetoacetate | 1.06 (1.02, 1.1) | 0.016 |
| Acetone | 1.03 (0.99, 1.08) | 0.158 |
| Creatinine | 0.96 (0.91, 1.01) | 0.134 |
| Albumin | 1.00 (0.95, 1.04) | 0.897 |
| Glycoprotein acetyls | 1.00 (0.96, 1.05) | 0.897 |
| Concentration of chylomicrons and extremely large VLDL particles | 0.96 (0.91, 1.00) | 0.122 |
| Total lipids in chylomicrons and extremely large VLDL | 0.95 (0.91, 1.00) | 0.077 |
| Phospholipids in chylomicrons and extremely large VLDL | 0.96 (0.91, 1.01) | 0.125 |
| Cholesterol in chylomicrons and extremely large VLDL | 0.96 (0.92, 1.01) | 0.131 |
| Cholesteryl esters in chylomicrons and extremely large VLDL | 0.96 (0.92, 1.01) | 0.158 |
| Free cholesterol in chylomicrons and extremely large VLDL | 0.96 (0.91, 1.01) | 0.125 |
| Triglycerides in chylomicrons and extremely large VLDL | 0.95 (0.91, 1.00) | 0.089 |
| Concentration of very large VLDL particles | 0.93 (0.89, 0.98) | 0.015 |
| Total lipids in very large VLDL | 0.93 (0.88, 0.97) | 0.009 |
| Phospholipids in very large VLDL | 0.93 (0.89, 0.98) | 0.016 |
| Cholesterol in very large VLDL | 0.93 (0.89, 0.98) | 0.015 |
| Cholesteryl esters in very large VLDL | 0.93 (0.89, 0.98) | 0.016 |
| Free cholesterol in very large VLDL | 0.93 (0.89, 0.98) | 0.016 |
| Triglycerides in very large VLDL | 0.93 (0.88, 0.97) | 0.012 |
| Concentration of large VLDL particles | 0.92 (0.88, 0.97) | 0.006 |
| Total lipids in large VLDL | 0.91 (0.87, 0.96) | 0.002 |
| Phospholipids in large VLDL | 0.92 (0.88, 0.97) | 0.005 |
| Cholesterol in large VLDL | 0.92 (0.88, 0.97) | 0.005 |
| Cholesteryl esters in large VLDL | 0.93 (0.89, 0.97) | 0.009 |
| Free cholesterol in large VLDL | 0.92 (0.88, 0.97) | 0.005 |
| Triglycerides in large VLDL | 0.91 (0.87, 0.96) | 0.002 |
| **Metabolites** | **HR (95% CI)** | **FDR** |
| Concentration of medium VLDL particles | 0.94 (0.90, 0.98) | 0.016 |
| Total lipids in medium VLDL | 0.92 (0.88, 0.96) | 0.003 |
| Phospholipids in medium VLDL | 0.94 (0.90, 0.98) | 0.016 |
| Cholesterol in medium VLDL | 0.96 (0.91, 1.00) | 0.073 |
| Cholesteryl esters in medium VLDL | 0.97 (0.93, 1.01) | 0.194 |
| Free cholesterol in medium VLDL | 0.94 (0.90, 0.99) | 0.027 |
| Triglycerides in medium VLDL | 0.91 (0.87, 0.96) | 0.002 |
| Concentration of small VLDL particles | 0.94 (0.90, 0.98) | 0.017 |
| Total lipids in small VLDL | 0.93 (0.89, 0.98) | 0.012 |
| Phospholipids in small VLDL | 0.94 (0.90, 0.98) | 0.020 |
| Cholesterol in small VLDL | 0.95 (0.91, 0.99) | 0.045 |
| Cholesteryl esters in small VLDL | 0.95 (0.91, 0.99) | 0.055 |
| Free cholesterol in small VLDL | 0.95 (0.91, 0.99) | 0.038 |
| Triglycerides in small VLDL | 0.93 (0.89, 0.98) | 0.013 |
| Concentration of very small VLDL particles | 0.98 (0.94, 1.03) | 0.541 |
| Total lipids in very small VLDL | 0.99 (0.94, 1.03) | 0.610 |
| Phospholipids in very small VLDL | 0.99 (0.95, 1.04) | 0.734 |
| Cholesterol in very small VLDL | 0.99 (0.95, 1.04) | 0.824 |
| Cholesteryl esters in very small VLDL | 1.00 (0.95, 1.04) | 0.897 |
| Free cholesterol in very small VLDL | 0.99 (0.95, 1.03) | 0.697 |
| Triglycerides in very small VLDL | 0.97 (0.93, 1.02) | 0.275 |
| Concentration of IDL particles | 0.98 (0.93, 1.02) | 0.349 |
| Total lipids in IDL | 0.98 (0.94, 1.03) | 0.563 |
| Phospholipids in IDL | 0.99 (0.94, 1.03) | 0.674 |
| Cholesterol in IDL | 0.98 (0.94, 1.03) | 0.570 |
| Cholesteryl esters in IDL | 0.98 (0.94, 1.03) | 0.530 |
| Free cholesterol in IDL | 0.99 (0.95, 1.04) | 0.827 |
| Triglycerides in IDL | 0.99 (0.95, 1.04) | 0.734 |
| Concentration of large LDL particles | 0.96 (0.92, 1.00) | 0.114 |
| Total lipids in large LDL | 0.96 (0.92, 1.00) | 0.096 |
| Phospholipids in large LDL | 0.96 (0.92, 1.01) | 0.128 |
| Cholesterol in large LDL | 0.96 (0.91, 1.00) | 0.089 |
| Cholesteryl esters in large LDL | 0.95 (0.91, 1.00) | 0.063 |
| Free cholesterol in large LDL | 0.97 (0.93, 1.02) | 0.282 |
| Triglycerides in large LDL | 0.99 (0.95, 1.04) | 0.723 |
| Concentration of medium LDL particles | 0.94 (0.90, 0.99) | 0.030 |
| Total lipids in medium LDL | 0.94 (0.90, 0.98) | 0.018 |
| Phospholipids in medium LDL | 0.94 (0.90, 0.98) | 0.023 |
| Cholesterol in medium LDL | 0.94 (0.90, 0.98) | 0.018 |
| Cholesteryl esters in medium LDL | 0.94 (0.90, 0.98) | 0.016 |
| Free cholesterol in medium LDL | 0.95 (0.91, 0.99) | 0.053 |
| Triglycerides in medium LDL | 0.97 (0.93, 1.02) | 0.296 |
| Concentration of small LDL particles | 0.95 (0.91, 0.99) | 0.038 |
| Total lipids in small LDL | 0.94 (0.90, 0.99) | 0.030 |
| Phospholipids in small LDL | 0.95 (0.91, 0.99) | 0.053 |
| Cholesterol in small LDL | 0.94 (0.90, 0.99) | 0.030 |
| Cholesteryl esters in small LDL | 0.94 (0.90, 0.99) | 0.030 |
| Free cholesterol in small LDL | 0.95 (0.91, 0.99) | 0.053 |
| **Metabolites** | **HR (95% CI)** | **FDR** |
| Triglycerides in small LDL | 0.96 (0.91, 1.00) | 0.12 |
| Concentration of very large HDL particles | 1.09 (1.04, 1.15) | 0.003 |
| Total lipids in very large HDL | 1.10 (1.05, 1.16) | 0.002 |
| Phospholipids in very large HDL | 1.11 (1.05, 1.16) | 0.001 |
| Cholesterol in very large HDL | 1.10 (1.04, 1.15) | 0.002 |
| Cholesteryl esters in very large HDL | 1.10 (1.04, 1.15) | 0.002 |
| Free cholesterol in very large HDL | 1.09 (1.04, 1.14) | 0.002 |
| Triglycerides in very large HDL | 1.00 (0.96, 1.05) | 0.971 |
| Concentration of large HDL particles | 1.10 (1.05, 1.16) | 0.002 |
| Total lipids in large HDL | 1.11 (1.05, 1.17) | 0.002 |
| Phospholipids in large HDL | 1.10 (1.05, 1.16) | 0.002 |
| Cholesterol in large HDL | 1.10 (1.05, 1.16) | 0.002 |
| Cholesteryl esters in large HDL | 1.10 (1.05, 1.16) | 0.002 |
| Free cholesterol in large HDL | 1.10 (1.05, 1.16) | 0.002 |
| Triglycerides in large HDL | 1.03 (0.98, 1.08) | 0.269 |
| Concentration of medium HDL particles | 1.06 (1.00, 1.11) | 0.063 |
| Total lipids in medium HDL | 1.05 (1.00, 1.10) | 0.113 |
| Phospholipids in medium HDL | 1.04 (0.99, 1.10) | 0.125 |
| Cholesterol in medium HDL | 1.06 (1.00, 1.11) | 0.062 |
| Cholesteryl esters in medium HDL | 1.06 (1.01, 1.11) | 0.061 |
| Free cholesterol in medium HDL | 1.06 (1.00, 1.11) | 0.061 |
| Triglycerides in medium HDL | 0.99 (0.94, 1.03) | 0.587 |
| Concentration of small HDL particles | 0.96 (0.92, 1.00) | 0.116 |
| Total lipids in small HDL | 0.97 (0.92, 1.01) | 0.192 |
| Phospholipids in small HDL | 0.98 (0.93, 1.02) | 0.411 |
| Cholesterol in small HDL | 0.96 (0.92, 1.01) | 0.158 |
| Cholesteryl esters in small HDL | 0.96 (0.92, 1.01) | 0.134 |
| Free cholesterol in small HDL | 0.98 (0.93, 1.03) | 0.433 |
| Triglycerides in small HDL | 0.95 (0.90, 1.00) | 0.061 |

Abbreviations: HR, hazard ratio; CI, confidence interval; FDR, false discovery rate; FDR, false discovery rate; HDL, high-density lipoprotein; LDL, low-density lipoprotein; VLDL, very low-density lipoprotein.

^*^ The models were adjusted for age, gender, ethnicity, family history of Alzheimer's disease, apolipoprotein E genotypes, smoking status, alcohol consumption, healthy diet, regular exercise, sleep duration, body mass index, waist circumference, systolic blood pressure, and diastolic blood pressure. FDR adjusted P values are shown in the table.

# Table S48. Mediation proportion of SES profiles in dementia attributed to blood inflammatory markers (N=303,981).^*^

|  | **Balanced-high SES subpopulation** | | **Low income-less educated subpopulation** | | **Low income-NSES subpopulation** | | **Low SES subpopulation** | |
| --- | --- | --- | --- | --- | --- | --- | --- | --- |
|  | **Mediation proportion (%) (95% CI)** | **FDR** | **Mediation proportion (%) (95% CI)** | **FDR** | **Mediation proportion (%) (95% CI)** | **FDR** | **Mediation proportion (%) (95% CI)** | **FDR** |
| Leukocyte count | Ref. | — | 1.3 (0.3, 2.1) | <0.001 | 0.6 (0.1, 1.0) | 0.040 | 0.5 (0.2, 0.9) | <0.001 |
| Neutrophil count | Ref. | — | 3.0 (1.5, 4.4) | <0.001 | 1.4 (0.8, 2.3) | <0.001 | 1.4 (0.7, 2.0) | <0.001 |
| Neutrophil percentage | Ref. | — | 3.1 (2.1, 4.9) | <0.001 | 1.7 (1.1, 3.0) | <0.001 | 1.8 (1.0, 2.7) | <0.001 |
| Monocyte percentage | Ref. | — | 0.4 (0.1, 1.0) | 0.021 | 0.2 (0.1, 0.5) | <0.001 | 0.5 (0.2, 0.8) | <0.001 |
| Lymphocyte count | Ref. | — | 0.4 (0.2, 0.8) | <0.001 | 0.6 (0.3, 1.0) | <0.001 | 0.5 (0.3, 0.8) | <0.001 |
| Lymphocyte percentage | Ref. | — | 3.6 (2.1, 6.0) | <0.001 | 2.4 (1.5, 3.6) | <0.001 | 2.1 (1.5, 2.9) | <0.001 |
| SII | Ref. | — | 3.3 (2.2, 5.2) | <0.001 | 1.9 (1.3, 2.8) | <0.001 | 1.8 (1.1, 2.3) | <0.001 |
| NLR | Ref. | — | 3.7 (2.6, 5.9) | <0.001 | 2.3 (1.6, 3.5) | <0.001 | 2.1 (1.5, 2.8) | <0.001 |
| PLR | Ref. | — | 1.0 (0.4, 1.7) | <0.001 | 0.7 (0.3, 1.2) | <0.001 | 0.6 (0.3, 0.9) | <0.001 |
| LMR | Ref. | — | 0.6 (0.1, 1.2) | 0.021 | 0.5 (0.1, 1.0) | <0.001 | 0.2 (0.0, 0.4) | <0.001 |

Abbreviations: SES, socioeconomic status; CI, confidence interval; FDR, false discovery rate; ISES, individual socioeconomic status; SII, systemic immune-inflammation index; NLR, neutrophil-to-lymphocyte ratio; PLR, platelet-to-lymphocyte ratio; LMR, lymphocyte-to-monocyte ratio.

^*^ The models were adjusted for age, gender, ethnicity, family history of Alzheimer's disease, apolipoprotein E genotypes, smoking status, alcohol consumption, healthy diet, regular exercise, sleep duration, body mass index, waist circumference, systolic blood pressure, and diastolic blood pressure. FDR adjusted P values are shown in the table.

# Table S49. Mediation proportion of SES profiles in dementia attributed to metabolites (N=174,175).^*^

|  | **Balanced-high SES subpopulation** | | **Low income-less educated subpopulation** | | **Low income-NSES subpopulation** | | **Low SES subpopulation** | |
| --- | --- | --- | --- | --- | --- | --- | --- | --- |
|  | **Mediation proportion (%) (95% CI)** | **FDR** | **Mediation proportion (%) (95% CI)** | **FDR** | **Mediation proportion (%) (95% CI)** | **FDR** | **Mediation proportion (%) (95% CI)** | **FDR** |
| LDL cholesterol | Ref. | — | 0.1 (-0.1, 0.6) | 0.294 | 0.3 (0.0, 0.8) | 0.13 | 0.1 (0.0, 0.3) | 0.069 |
| HDL cholesterol | Ref. | — | -0.1 (-0.6, 0.1) | 0.278 | 0.7 (0.2, 1.7) | <0.001 | 0.3 (0.0, 0.5) | 0.047 |
| Phospholipids in HDL | Ref. | — | 0.4 (0.1, 1.0) | <0.001 | 0.9 (0.3, 2.0) | <0.001 | 0.4 (0.1, 0.8) | 0.024 |
| Cholesteryl esters in HDL | Ref. | — | -0.1 (-0.4, 0.3) | 0.700 | 0.8 (0.2, 1.8) | <0.001 | 0.2 (0.1, 0.5) | <0.001 |
| Free cholesterol in HDL | Ref. | — | -0.6 (-1.4, -0.2) | <0.001 | 0.5 (0.1, 1.2) | 0.024 | 0.3 (0.1, 0.6) | <0.001 |
| Total lipids in HDL | Ref. | — | 0.2 (0.0, 0.6) | 0.088 | 0.7 (0.0, 1.7) | 0.088 | 0.4 (0.1, 0.8) | 0.024 |
| Degree of unsaturation | Ref. | — | 2.0 (0.2, 4.5) | <0.001 | 1.3 (0.3, 3.4) | <0.001 | 0.8 (0.2, 1.5) | <0.001 |
| Omega-3 fatty acids | Ref. | — | 2.3 (0.9, 5.0) | <0.001 | 2.4 (0.9, 4.8) | <0.001 | 0.5 (0.3, 1.0) | <0.001 |
| Docosahexaenoic acid | Ref. | — | 2.4 (0.5, 5.1) | 0.024 | 1.9 (0.5, 4.1) | <0.001 | 0.6 (0.2, 1.1) | <0.001 |
| Alanine | Ref. | — | 2.2 (0.9, 3.5) | <0.001 | 1.3 (0.5, 3.2) | <0.001 | 0.7 (0.3, 1.2) | <0.001 |
| Total concentration of branched-chain amino acids (leucine + isoleucine + valine) | Ref. | — | 4.5 (2.5, 8.7) | <0.001 | 3.6 (2.1, 6.7) | <0.001 | 2.6 (1.6, 3.8) | <0.001 |
| Isoleucine | Ref. | — | 2.2 (0.9, 4.2) | <0.001 | 1.5 (0.9, 2.5) | <0.001 | 1.5 (0.8, 2.3) | <0.001 |
| Leucine | Ref. | — | 2.3 (1.3, 4.4) | <0.001 | 2.5 (1.4, 4.7) | <0.001 | 2.1 (1.2, 3.3) | <0.001 |
| Valine | Ref. | — | 6.4 (3.9, 10.6) | <0.001 | 4.1 (2.6, 8.3) | <0.001 | 3.0 (2.0, 4.5) | <0.001 |
| Phenylalanine | Ref. | — | -0.1 (-0.4, 0.2) | 0.467 | 0.1 (-0.1, 0.5) | 0.372 | 0.7 (0.2, 1.3) | <0.001 |
| Tyrosine | Ref. | — | 0.9 (0.2, 1.9) | 0.024 | 0.7 (0.3, 1.6) | <0.001 | 0.7 (0.2, 1.2) | 0.024 |
| 3-Hydroxybutyrate | Ref. | — | 0.9 (0.3, 2.1) | <0.001 | 1.1 (0.4, 2.2) | <0.001 | 1.0 (0.4, 1.6) | 0.024 |
| Acetoacetate | Ref. | — | 0.4 (0.1, 1.0) | <0.001 | 1.3 (0.4, 3.0) | <0.001 | 0.6 (0.2, 1.1) | <0.001 |
| Concentration of large HDL particles | Ref. | — | -2.3 (-4.5, -1.0) | <0.001 | 0.4 (0.1, 0.9) | <0.001 | 0.2 (0.1, 0.5) | <0.001 |
| Total lipids in large HDL | Ref. | — | -1.8 (-4.0, -0.9) | <0.001 | 0.5 (0.1, 1.1) | <0.001 | 0.2 (0.0, 0.5) | <0.001 |
| Phospholipids in large HDL | Ref. | — | -1.6 (-3.6, -0.6) | <0.001 | 0.6 (0.2, 1.4) | <0.001 | 0.3 (0.1, 0.6) | <0.001 |

Abbreviations: SES, socioeconomic status; CI, confidence interval; FDR, false discovery rate; HDL, high-density lipoprotein; LDL, low-density lipoprotein.

^*^ The models were adjusted for age, gender, ethnicity, family history of Alzheimer's disease, apolipoprotein E genotypes, smoking status, alcohol consumption, healthy diet, regular exercise, sleep duration, body mass index, waist circumference, systolic blood pressure, and diastolic blood pressure. FDR adjusted P values are shown in the table.

**Supplementary References:**

[1] Spiegelman D, Hertzmark E, Wand HC. Point and interval estimates of partial population attributable risks in cohort studies: examples and software. *Cancer Causes Control*. 2007;18:571-9. <https://doi.org/10.1007/s10552-006-0090-y>
